# Tick Haller’s Organ, a New Paradigm for Arthropod Olfaction: How Ticks Differ from Insects

**DOI:** 10.3390/ijms18071563

**Published:** 2017-07-18

**Authors:** Ann L. Carr, Robert D. Mitchell III, Anirudh Dhammi, Brooke W. Bissinger, Daniel E. Sonenshine, R. Michael Roe

**Affiliations:** 1 Department of Entomology and Plant Pathology, North Carolina State University, Raleigh, NC 27695, USA; alcarr2@ncsu.edu (A.L.C.); rdmitche@ncsu.edu (R.D.M.); adhammi@ncsu.edu (A.D.); 2 AgBiome, Research Triangle Park, NC 27709, USA; bbissinger@agbiome.com; 3 Department of Biological Sciences, Old Dominion University, Norfolk, VA 23529, USA; dsonensh@odu.edu

**Keywords:** American dog tick, *Dermacentor variabilis*, tick, Haller’s organ, olfaction, gustation, chemoreception, odorant binding proteins, GPCR, DEET, transcriptome

## Abstract

Ticks are the vector of many human and animal diseases; and host detection is critical to this process. Ticks have a unique sensory structure located exclusively on the 1st pairs of legs; the fore-tarsal Haller’s organ, not found in any other animals, presumed to function like the insect antennae in chemosensation but morphologically very different. The mechanism of tick chemoreception is unknown. Utilizing next-generation sequencing and comparative transcriptomics between the 1st and 4th legs (the latter without the Haller’s organ), we characterized 1st leg specific and putative Haller’s organ specific transcripts from adult American dog ticks, *Dermacentor variabilis*. The analysis suggested that the Haller’s organ is involved in olfaction, not gustation. No known odorant binding proteins like those found in insects, chemosensory lipocalins or typical insect olfactory mechanisms were identified; with the transcriptomic data only supporting a possible olfactory G-protein coupled receptor (GPCR) signal cascade unique to the Haller’s organ. Each component of the olfactory GPCR signal cascade was identified and characterized. The expression of GPCR, G_αo_ and β-arrestin transcripts identified exclusively in the 1st leg transcriptome, and putatively Haller’s organ specific, were examined in unfed and blood-fed adult female and male *D. variabilis*. Blood feeding to repletion in adult females down-regulated the expression of all three chemosensory transcripts in females but not in males; consistent with differences in post-feeding tick behavior between sexes and an expected reduced chemosensory function in females as they leave the host. Data are presented for the first time of the potential hormonal regulation of tick chemosensation; behavioral assays confirmed the role of the Haller’s organ in *N*,*N*-diethyl-meta-toluamide (DEET) repellency but showed no role for the Haller’s organ in host attachment. Further research is needed to understand the potential role of the GPCR cascade in olfaction.

## 1. Introduction

Ticks are blood feeding ectoparasites that may cause direct harm to humans and animals. They inflict painful wounds during feeding, as well as vector microbial agents that can cause debilitating diseases. In the US, the American dog tick, *Dermacentor variabilis* (Acari: Ixodidae), is the most prolific member of the genus and the primary vector of *Rickettsia rickettsii*, the causative agent of Rocky Mountain spotted fever [1,2]. Tick development requires blood feeding for molting, metamorphosis, sexual maturation and reproduction; blood feeding provides the means for pathogen transmission between the tick and its host [3,4].

Successful blood feeding is dependent on the efficient detection of hosts within the proximate environment of the tick. Ticks rely heavily on chemosensation to identify and locate hosts. Chemosensation is the main function ascribed to the Haller’s organ, a unique structure found on the foretarsus of the first pair of legs. The Haller’s organ is not found in any other animals. In addition to detecting host kairomones, Haller’s organ chemoreceptors are also involved in the detection of pheromones, aggregation chemicals, and environmental cues needed for life off the host [5]. Despite the pivotal role of the Haller’s organ as the primary component of the tick peripheral sensory system, and its presence only in Acari, little is known about its genetic structure or molecular function. The Haller’s organ is essentially a molecular black box with no information on its mechanism of chemosensation.

Through next-generation sequencing and comparative transcriptomics between the 1st and 4th pair of legs (the latter without the Haller’s organ) in *D. variabilis*, we generated a 1st leg specific transcriptome, putatively containing Haller’s organ specific transcripts. These transcripts were then compared to those associated with chemosensation in insects and nematodes to identify putative odorant binding proteins (OBPs), odorant receptors (ORs), gustatory receptors (GRs), odorant receptor kinases (ORKs) and odorant degrading enzymes (ODEs), and to determine the mechanism of chemoreception in ticks [6,7]. The protein functions of putative tick chemosensory transcripts were characterized through sequence alignments, phylogenetic analyses, identification of conserved functional domains, and gene expression. The findings in this study are consistent with the role of the Haller’s organ in olfaction but not gustation, despite well documented morphological evidence of gustatory-like sensilla [1]. There was no evidence that ticks detected odorants using the same mechanism described for insects. We found a G-protein coupled receptor (GPCR) signal cascade that lacks the OBPs described in insects. Gene expression of olfactory transcripts before and after blood feeding suggest links between olfaction, host attraction, and tick endocrinology, providing new leads for the development of tick acaricides and repellents*.* Behavioral bioassays provided a new understanding of the role of the Haller’s organ in tick repellency and in host seeking versus blood feeding.

## 2. Results and Discussion

### 2.1. Sequencing and Transcriptomic Assembly

Two transcriptome datasets were generated using normalized 1st and 4th leg cDNA libraries and Illumina Hiseq technology (Illumina, San Diego, CA, USA). In total, 106 million reads were obtained for the 1st legs, assembled into 88,289 contigs establishing the Illumina 1st leg transcriptome; 180 million reads were obtained for the 4th legs, assembled into 105,827 contigs establishing the Illumina 4th leg transcriptome. Using Blast2GO (BioBam, Valencia, Spain) and the GenBank non-redundant database, at least one putative function with an expect value (*e*-value) of <10 was identified for 71,114 of the Illumina 1st leg contigs and 83,647 of the Illumina 4th leg contigs. For information on gene ontology (GO) mapping of the 1st and 4th leg contigs see Supplementary Materials (Figure S1). Due to the exclusive location of the Haller’s organ on the 1st pair of legs, an in silico subtraction was performed between the Illumina 1st and 4th leg contigs for only those contigs with putative functions. Removal of Illumina 1st leg contigs with identical counterparts, based on function and accession number, in the Illumina 4th leg transcriptome resulted in the identification of 38,087 contigs exclusive to the 1st legs, and what will be referenced in this paper as the Haller’s organ spf (specific) transcriptome. Permission was also obtained to include a combined unfed virgin adult female and male *D. variabilis* 1st leg transcriptome, generated using 454 pyrosequencing, in Basic Local Alignment Search Tool (BLAST) searches for chemosensory transcripts. The 454 1st leg transcriptome fasta file contained 33,981 contigs with at least one putative function with an *e*-value of <10 identified for 22,151 of the contigs. For information on GO mapping of the 454 1st leg contigs see Supplementary Materials (Figure S1). The nomenclature for the four transcriptomes used throughout this manuscript will be as follows: the 454 1st leg transcriptome, the Haller’s organ spf transcriptome, the Illumina 1st leg transcriptome, and the Illumina 4th leg transcriptome.

### 2.2. Top 50 Most Abundant Transcripts in the Illumina 1st and 4th Leg Transcriptomes

Table 1 and Table 2 list the top 50 most abundant transcripts identified in the Illumina 1st and 4th leg transcriptomes, respectively. All contigs had sequence similarity to genes in the Uniprot knowledgebase, though several contigs listed in Table 1 and Table 2 were homologous to genes of unknown function, including the foremost abundant contigs in both transcriptomes. Numerous contigs sequenced with high frequency were homologous to components of striated muscle. Since oblique leg muscles insert in tick legs, it is expected that the Illumina 1st and 4th leg transcriptomes contain components of striated (skeletal) muscle [8]. Tick striated muscle myofibrils are composed of a series of contractile units called sarcomeres that extend from one Z-line to the successive Z-line. Sarcomeres are composed of interspersed actin and myosin filaments responsible for muscle contraction [9]. One 1st leg transcript (contig 73) and one 4th leg transcript (contig 146) both had high homology (100%) to *Rhipicephalus microplus* actin filament (AAP79880) and were sequenced 278,198 and 146,196 times, respectively (Table 1 and Table 2, Figure S2). Seven 1st leg transcripts (contigs 27862, 15573, 452, 430, 233, 36, and 60) and three 4th leg transcripts (contigs 86, 468, and 79) with high homology (95%) to *Amblyomma aureolatum* myosin filament (JAT93369.1) were each sequenced several times (Table 1 and Table 2, Figure S3).

Troponin is a protein complex that is vital in regulating muscle contraction. Troponin is present on actin filaments and acts on the coiled coil protein tropomyosin. When inactive, the troponin-tropomyosin complex blocks myosin binding sites. Calcium ions activate troponin and cause a conformational change of the troponin-tropomyosin complex that exposes the myosin binding sites for muscle contraction [9]. The troponin protein complex consists of three subunits: the tropomyosin-binding subunit (troponin T), the inhibitory subunit (troponin I), and the calcium-binding subunit (troponin C) [10]. Only transcripts homologous to the troponin T and I subunits were identified among the top 50 most abundant transcripts of the Illumina 1st and 4th leg transcriptomes. One 1st leg transcript (contig 22) and one 4th leg transcript (contig 42) both had high sequence homology (93%) to *R. appendiculatus* troponin T (JAP88178.1), and were sequenced 410,660 and 316,355 times, respectively (Table 1 and Table 2, Figure S4). One 1st leg transcript (contig 33) and two 4th leg transcripts (contigs 194 and 51752) were sequenced in high frequency with significant homology to troponin I and the wings’ up A protein (Table 1 and Table 2). Contigs homologous to the coiled coil protein tropomyosin were also identified in the top 50 most abundant transcripts of the Illumina 1st and 4th leg transcriptomes. One transcript (contig 17) in the Illumina 1st leg transcriptome and one transcript (contig 239) in the Illumina 4th leg transcriptome both had high sequence homology (100%) to *R. microplus* tropomyosin (AMB19056.1) and were sequenced 72,240 and 89,239 times, respectively (Table 1 and Table 2, Figure S5). Titin is a giant elastic protein found in striated muscle sarcomeres that connects myosin to the Z-line. Titin allows for muscle cell elasticity and is essential for ensuring the mechanical stability of muscle fibers [9]. Three 1st leg transcripts (contigs 4832, 1517, and 696) and two 4th leg transcripts (contigs 6805 and 680) had sequence homology to the *Ixodes scapularis* myofibril scaffold component titin (EEC05627.1) and were each sequenced several times (Table 1 and Table 2, Figure S6).

One transcript in the Illumina 1st transcriptome and one transcript in the 4th leg transcriptome were each sequenced in high frequency with significant homology to an *I. scapularis* cuticle protein (EEC04237.1; Table 1 and Table 2). The integument of ticks consists of an epidermis and its secreted cuticle [11]. Since tick legs are surrounded externally by cuticle, it is not surprising to identify cuticle proteins among the top 50 most abundant transcripts of the Illumina 1st and 4th leg transcriptomes. Remaining contigs in the top 50 most abundant transcripts of the Illumina 1st and 4th leg transcriptomes were putative housekeeping genes including, cytoskeletal proteins, endocytosis proteins, heat shock proteins, transcription factors, and proteins involved in cellular metabolism and adenosine triphosphate (ATP) production (Table 1 and Table 2) [11,12].

### 2.3. No Odorant Binding Proteins Found in Ticks

BLASTx (Basic Local Alignment Search Tool, translated nucleotide to protein) and BLASTn (Basic Local Alignment Search Tool, nucleotide to nucleotide) searches of the 454 1st leg, Haller’s organ spf, Illumina 1st leg, and Illumina 4th leg transcriptomes did not identify any transcripts putatively encoding odorant binding proteins (OBP) or pheromone binding proteins (PBP; *e*-value ≤ 1.0). To further validate these findings, the NCBI (National Center for Biotechnology Information, Bethesda, MD, USA) BLAST+ toolkit and “makeBLASTdb” UNIX coding were used to create a BLASTable Illumina 1st leg BLAST database from the Illumina 1st leg transcriptome fasta file and a BLASTable Illumina 4th leg BLAST database from the Illumina 4th leg transcriptome fasta file. Both the Illumina 1st and 4th leg BLAST databases were uploaded into the program Geneious (Biomatters, Auckland, New Zealand); tBLASTn (Basic Local Alignment Search Tool, protein to translated nucleotide) searches of the Illumina 1st leg BLAST database were conducted for OBPs and PBPs (*e*-value ≤ 1.0). OBPs and PBPs have been well characterized in the Dipteran species *Aedes aegypti*, *Anopheles gambiae* and *Drosophila melanogaster.* All the OBPs and PBPs for these Dipteran species reviewed and verified by Uniprot and present in the Uniprot-Swissprot knowledgebase (Appendix A) were used in tBLASTn searches of the Illumina 1st leg BLAST database and no putative OBPs or PBPs identified (*e*-value ≤ 1.0). BLASTn and BLASTx searches of all the tick and mite sequence data in GenBank using the same OBPs and PBPs described above also did not identify any putative OBPs or PBPs (*e*-value ≤ 1.0). OBPs and PBPs were not present in any of our four transcriptomes and were also not present in any of the tick or mite sequence data in GenBank or in the *Ixodes scapularis* genome [13]. Renthal et al. [14] reported the identification of two OBP-like expressed sequence tag coded proteins (EST; JZ183505.1 and JZ172282.1) in the foretarsus proteome of the lone star tick *Amblyomma americanum*. Unfortunately, tBLASTn searches of our Illumina 1st and 4th leg BLAST databases determined that the putative OBP-like ESTs were not exclusive to the 1st pair of legs. Numerous transcripts homologous to the OBP-like EST JZ183505.1 were identified in both the 1st and 4th legs. The percent identity between one such 4th leg transcript (contig 343) and the OBP-like EST JZ183505.1 was calculated to be 73%, with both transcripts having significant homology to the same BLASTx GenBank hit, a putative *A. americanum* secreted protein (JAG92140.1; Figure S7). Interestingly, only one transcript homologous to the OBP-like EST JZ172282.1 was identified, and in only the 4th legs. tBLASTn searches of the Illumina 1st leg BLAST database for the OBP-like EST JZ172282.1 did not identify any matches. The percent identity between the 4th leg transcript (contig 114) and the OBP-like EST JZ172282.1 was calculated to be 89%, with both transcripts having high homology (100%) to the same BLASTx GenBank hit, a putative *A. americanum* secreted protein (JAG92350.1; Figure S8). Additional EST coded proteins theorized to function as OBPs in ticks, such as the dust-mite antigen, neto-like protein, Niemann-Pick C2, and microplusin were either identified in the Illumina 4th leg transcriptome and not exclusive to the 1st legs or missing from all transcriptome datasets completely [14].

**Figure 1:**
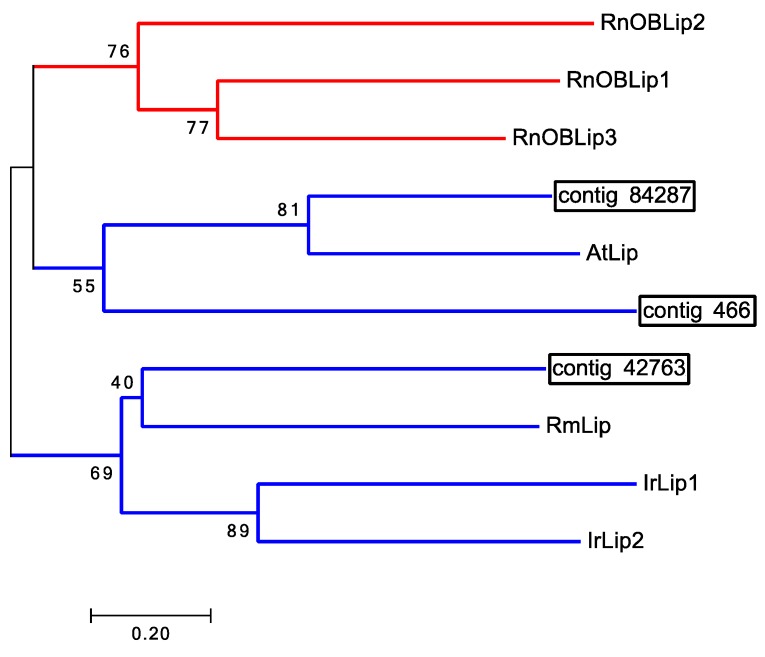
Phylogenetic relationship of transcripts putatively encoding lipocalins (Lip) identified in the Haller’s organ spf (contig 84287) and the Illumina 1st and 4th leg transcriptomes (contigs 42763, 1st legs; contig 466, 4th legs) of unfed, virgin adult male *Dermacentor variabilis* with lipocalins from *Amblyomma triste, Ixodes ricinus, Rhipicephalus microplus* and *Rattus norvegicus*. The phylogenetic tree shows the branch relation of chemosensory lipocalins (red branch) with non-chemosensory lipocalins. Tick putative lipocalins are highlighted with a blue branch color. Acronyms are as follows: first letter of the genus and species (*Amblyomma triste*, At; *Ixodes ricinus*, Ir; *Rattus norvegicus*, Rn; *Rhipicephalus microplus*, Rm) followed by the protein name (OBlip or Lip). Putative lipocalin transcripts are boxed. The tree was constructed using Maximum likelihood phylogenetic analysis and bootstrapping set to 500 iterations. Branch values listed are bootstrap percentages (percent confidence), scale set to 20%. A comprehensive list of acronyms and associated GenBank accession numbers are listed in Appendix A.

In vertebrates, the OBPs are lipocalins with no sequence homology with insect OBPs [15]. Vertebrate chemosensory lipocalins are identified exclusively in chemosensory tissues, and are structurally different from non-chemosensory lipocalins. Since there were no insect-like OBPs or PBPs identified in any of our transcriptomes or the other tick databases examined, we searched for possible tick lipocalins associated with chemosensation in the 1st legs. One transcript (contig 84287) encoding a lipocalin was identified exclusively in the Haller’s organ spf transcriptome (Table 3). Two additional unique transcripts were found in the Illumina 1st leg transcriptome (contigs 466 and 42763) which were homologous to two transcripts in the Illumina 4th leg transcriptome (contigs 39297 and 24762), respectively. Contigs 39297 and 466 were homologs to the putative chemosensory lipocalin EST JZ171538.1 identified by Renthal et al. [14] (Figure S9) in the foretarsus proteome of the lone star tick, *A. americanum*. Since homologous transcripts were found in both the front and back legs, and there are no known chemosensory organs on the latter, this argues that the *A. americanum* lipocalin EST JZ171538.1 is not acting as an OBP. BLASTx analysis of the identified putative *D. variabilis* Haller’s organ spf lipocalin (contig 84287) determined the top GenBank hit (lowest *e*-value) to be an *A. triste* lipocalin (JAC30054.1). An alignment between the Haller’s organ spf lipocalin (contig 84287) and the *A. triste* lipocalin (JAC30054.1) showed a significant number of conserved residues despite the short nature of the Haller’s organ spf lipocalin (Figure S10). Phylogenetic analysis of the putative Haller’s organ spf lipocalin (contig 84287) suggested it was not related to vertebrate chemosensory lipocalins (Figure 1). The putative lipocalin did not cluster in the same node as the vertebrate chemosensory lipocalins. Phylogenetic analysis also showed that the Haller’s organ spf lipocalin (contig 84287) clustered in the same node as the 4th leg lipocalin, contig 466, homologous to contig 39297 of the 1st legs (Figure 1). The 4th leg lipocalin, contig 466, was used in phylogenetic analyses instead of the homologous 1st leg contig 39297 due to its longer sequence length. With no lipocalins clearly identified exclusively in the Haller’s organ spf transcriptome and that were related to vertebrate chemosensory lipocalins, it is unlikely that tick lipocalins function as chemosensory lipocalins. To further verify these findings, BLASTn and BLASTx searches of all the tick and mite sequence data in GenBank were performed searching for vertebrate chemosensory lipocalins with no putative lipocalins (*e*-value ≤ 10) found.

In summary, there is no evidence in our multiple transcriptomes, in current tick and mite sequences in GenBank, or in the *I. scapularis* genome that ticks use OBPs, PBPs or chemosensory lipocalins for chemosensation. These same proteins also are not involved in chemosensation in nematodes [6] suggesting ticks are more like nematodes than insects, they have a novel, unknown class of binding proteins, and/or they use other methods of sensilla lymph solubilization not yet described to deliver odorant molecules from the environment to chemoreceptors.

### 2.4. Haller’s Organ Not Involved in Gustation

The multipore and tip-pore sensilla of the Haller’s organ suggest an olfactory and gustatory function, respectively. Interestingly, BLASTx searches of the 454 1st leg, Illumina 1st leg, Illumina 4th leg, and Haller’s organ spf transcriptomes did not find any gustatory receptors (GR; *e*-value ≤ 1). Fifty-six GRs were identified in the *I. scapularis* genome; annotation of the *I. scapularis* GRs determined that they were GPCRs, as well as members of the 7-transmembrane chemosensory receptor family [13,16]. tBLASTn searches of the Illumina 1st leg BLAST database for the 56 *I. scapularis* GRs identified three putative uncharacterized 7-transmembrane receptor family transcripts. Unfortunately, homologs to these three transcripts were also identified in the 4th legs, thus not exclusive to the 1st legs or Haller’s organ making it unlikely that these transcripts function in chemosensation. The lack of 1st leg specific GRs suggests that the primary role of the Haller’s organ is olfaction, and that there is a secondary chemosensory organ in ticks, most likely on the pedipalps, associated with gustation. These findings are consistent with results obtained from bioassays that determined the Haller’s organ is not required for host biting or feeding, and only functions in host seeking and repellent detection, discussed later in further detail.

### 2.5. Ionotropic Glutamate Receptors Not Involved in Haller’s Organ Olfaction

Current studies of olfaction in higher order Diptera has led to the discovery of a newly classified family of olfactory receptors, ionotropic receptors (IR); IRs are an olfactory type of ionotropic glutamate receptor (iGluR) [17]. BLASTx searches of the 454 1st leg, Haller’s organ spf, Illumina 1st leg, and Illumina 4th leg transcriptomes identified 17 iGluR transcripts. tBLASTn searches of the Illumina 1st and 4th leg BLAST databases for olfactory IRs were also conducted to ensure a thorough screen for putative IRs in our transcriptomic datasets. IRs in *D. melanogaster* have been well characterized. All the IRs for *D. melanogaster* reviewed and verified by Uniprot and present in the Uniprot-Swissprot knowledgebase (Appendix A) were used in tBLASTn searches of the Illumina 1st and 4th leg BLAST databases. Due to the small number of reviewed and verified *D. melanogaster* IRs, additional tBLASTn searches of the Illumina 1st and 4th leg BLAST databases were conducted including unreviewed IRs in *A. gambiae* (Appendix A) [17]. tBLASTn searches identified an additional 10 iGluR transcripts, and two IR25a homologs (contig 69992, 1st legs; contig 3407, 4th legs). No other IR homologs were identified in any of the BLAST searches of the transcriptome datasets. Unfortunately, none of the identified iGluR transcripts, including the two IR25a homologs, were found exclusively in the 1st legs. All of the identified transcripts were common to both the 1st and 4th legs. The lack of specificity of the identified IRs and iGluRs transcripts to the forelegs suggests that IRs and iGluRs are not the olfactory receptors in the Haller’s organ. As mentioned before, there is no evidence of olfactory organs on the hind legs in ticks.

The identification of IR25a transcripts in both the 1st and 4th legs can be explained by a recent discovery linking IR25a to circadian rhythms. It has been determined that IR25a present in *Drosophila* leg stretch receptor neurons is required for temperature synchronization of the circadian clock [18]. IR25a detects small changes in temperature, distinguishing between day and nighttime temperatures and helps to reset the circadian clock at the end of the ~24 h cycle. It is possible that the IR25a transcripts identified in the 1st and 4th legs of *D. variabilis* are present throughout the peripheral nervous system of ticks and are responsible for resetting the circadian clock. More research is needed to understand the function of the IRs found in tick legs in our studies.

### 2.6. Transient Receptor Potential Channels Not Involved in Haller’s Organ Olfaction

Extensive research and characterization of transient receptor potential channels (TRP) in insects has led to the identification of antennal specific TRP splice variants in the “A” subfamily (TRPA) that putatively function in olfaction [19,20,21]. BLASTx and BLASTn searches of the 454 1st leg, Haller’s organ spf, Illumina 1st leg, and Illumina 4th leg transcriptomes did not identify any transcripts putatively encoding TRPAs (*e*-value ≤ 1.0). tBLASTn searches of the Illumina 1st and 4th leg BLAST databases for *I. scapularis* TRPA (EEC13968.1) homologs identified one putative TRPA, that was common to both the Illumina 1st and 4th leg transcriptomes (contig 8166, 1st legs; contig 4943, 4th legs). With the identified Illumina 1st and 4th leg TRPA transcripts having such low sequence homology (34%) to the *I. scapularis* TRPA (EEC13968.1), it is questionable if the identified TRPA transcripts are indeed members of the “A” subfamily, and not another TRP subfamily. The Illumina 1st and 4th leg TRPA transcripts (contig 8166, 1st legs; contig 4943, 4th legs) also had very low sequence homology to insect olfactory TRPA splice variants resulting in poor alignments; conserved residues were only found in the copies of Ankyrin repeats (Appendix A; Figure S11). With a lack of supporting evidence validating the identification of the putative TRPA transcripts, and its presence in both the 1st and 4th legs, it is unlikely TRPAs are used in an olfactory capacity in the Haller’s organ.

### 2.7. G-Protein Coupled Receptors Associated with Chemoreception in Ticks

Since gustatory receptors in ticks are 7-transmembrane receptor family G-protein coupled receptors (GPCR), a reasonable hypothesis might be that tick olfactory receptors are also 7-transmembrane receptor family GPCRs. A total of 28 putative GPCRs were identified in the 454 1st leg, Illumina 1st leg and Illumina 4th leg transcriptomes. One putative chemosensory GPCR (contig 72702) and one photosensory GPCR (contig 83622) were identified exclusively in the Haller’s organ spf transcriptome (Figures S12 and S13, Table 3). In insects, GPCRs can be divided into 4 clades, clade A (rhodopsin), B (secretin), C (metabotropic glutamate) and D (atypical) [22]. All GPCR chemoreceptors in insects and *C. elegans* belong to either clade A or D, with expression exclusively in chemosensory organs [6,23,24]. Gene ontology (GO) annotation and phylogenetic analyses of the putative Haller’s organ spf GPCR transcripts determined that both transcripts were putative clade A, rhodopsin-like GPCRs showing GPCR and photoreceptor activity (GO term identification no. GO:0009881, GO:0004930; Figure 2). With such few olfactory GPCRs identified in the Haller’s organ spf transcriptome, and the identified transcripts short in nature, additional BLAST searches were performed in attempts to identify more olfactory GPCRs or additional olfactory receptors of a variant type. tBLASTn searches of the Illumina 1st leg BLAST database were performed looking for analogues to the *C. elegans* chemosensory GPCRs str-2 and odr-3, the insect OR co-receptor OR83b also known as Orco and several randomly selected odorant receptors from *D. melanogaster* with no new GPCRs or ORs found (Appendix A; *e*-value ≤ 1.0). tBLASTn searches of the Illumina 1st and 4th leg BLAST databases were also conducted looking for putative chemosensory GPCRs identified by Munoz et al. in the *Rhipicephalus australis* 1st leg transcriptome [25]. Unfortunately, tBLASTn searches of our Illumina 1st and 4th leg BLAST databases determined that the putative *R. australis* chemosensory GPCRs were not exclusive to the 1st pair of legs. Numerous transcripts homologous to the *R. australis* chemosensory GPCRs were identified in both the 1st and 4th legs, with the percent identity ranging from 92–97%. It can be argued to some degree that the identified putative rhodopsin-like GPCR transcripts, exclusive to the Haller’s organ spf transcriptome, are olfactory/photosensory receptors. In *Drosophila* and *Anopheles*, there has been documentation of bimodal expression of olfactory and photosensory signal transduction proteins in strictly olfactory tissues. Arrestin for example, originally thought to function strictly in photoreception has been not only identified in olfactory tissues, but is required for proper olfactory function [26]. It is possible that there are some shared elements between tick olfaction and photoreception, and the identified rhodopsin GPCR transcripts act as receptors for either function in the Haller’s organ. The short sequences in our transcriptomes and the limitations in tick genomic data in general has made the assignment of function challenging and more work will be needed. It is also probable that tick olfactory receptors represent a completely novel type of 7-transmembrane receptor family proteins that have yet to be identified.

### 2.8. G-Proteins Associated with Odorant Reception

G-proteins are the intracellular components of GPCRs that initiate intracellular signaling cascades in response to extracellular stimuli that bind GPCRs. G-proteins consist of 3 subunits G_α_, G_β_, and G_γ_ [27]. The binding of a ligand to the GPCR results in a conformational change that activates the G_α_ subunit and prompts the dissociation of all G-protein subunits (see illustration in Table 3). G_α_ subunits function to activate secondary messengers such as adenylate/guanylate cyclase, whereas G_β_ and G_γ_ subunits form a dimer and function as signal modulators of G_α_ [27]. In the Haller’s organ spf transcriptome, two transcripts were identified putatively encoding chemosensory-specific G_α_ and G_β_ subunits (contig 13937, G_α_; contig 24477, G_β_; Table 3). In *C. elegans* and insect chemosensory organs, particular clades of the G_α_, G_β_, and G_γ_ subunits are exclusively expressed in chemosensory neurons while others are distributed throughout the whole organism [6,28].

**Figure 3:**
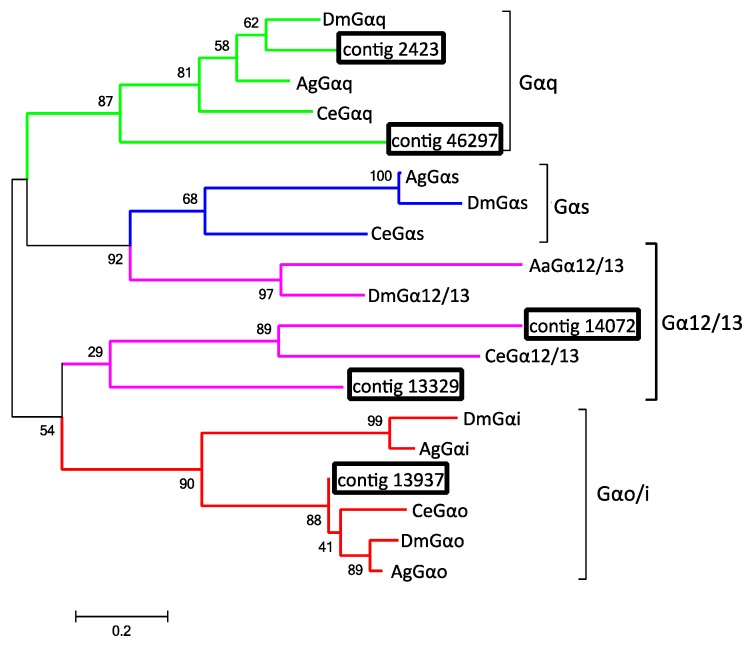
Phylogenetic relationship of transcripts putatively encoding G-protein α subunits (Gα), one transcript identified in the Haller’s organ spf transcriptome (contig 13937) and transcripts found common in the Illumina 1st and Illumina 4th leg transcriptomes (contigs 14072 and 46297, 1st legs; contigs 2423 and 13329, 4th legs) of unfed, virgin adult male *Dermacentor variabilis* with Gα subunits of known clade annotation from *Caenorhabditis elegans* and insects. The phylogenetic tree shows four clades, each represented by the following branch colors: red = Gαi/o clade; green = Gαq clade; purple = Gαq12/13 clade; blue = Gαs clade. Acronyms are as follows: first letter of the genus and species (*Anopheles aquasalis*, Aa; *Anopheles gambiae*, Ag; *Drosophila melanogaster*, Dm; *Caenorhabditis elegans*, Ce) followed by the protein name (Gα) and the letter/number of the associated clade. Putative Gα subunit transcripts are boxed. The tree was constructed using Maximum likelihood phylogenetic analysis and bootstrapping set to 500 iterations. Branch values listed are bootstrap percentages (percent confidence), scale set to 20%. A comprehensive list of acronyms and associated GenBank accession numbers are listed in Appendix A.

In addition to the G_α_ and G_β_ subunits identified in the Haller’s organ spf transcriptome, additional transcripts putatively encoding two G_α_ subunits and one G_β_ subunit were found, common, in the Illumina 1st leg, and Illumina 4th leg transcriptomes, and included in phylogenetic analyses. The G_α_ subunits can be classified into four clades: G_αi_/G_αo_, G_αq_, G_αs_, and G_12/13_ [29]. Alignment and phylogenetic analysis of all the identified putative G_α_ subunit transcripts determined that the Haller’s organ spf transcript (contig 13937) encoded a putative G_αo_ subunit, while the transcripts common to the Illumina 1st and Illumina 4th leg transcriptomes (contigs 14072 and 46297, 1st legs; contigs 2423 and 13329, 4th legs) encoded G_αq_ and G_α12/13_ subunits (Figure 3 and Figure S14). GO annotation and pathway identification of the putative G_α12/13_, G_αo_ and G_αq_ subunits revealed functional roles in the GPCR signal pathway and signal transduction (GO:0007186, GO:0007165). G_α12/13_ subunits are primarily associated with cell proliferation, cytoskeleton remolding and calcium signaling and are not of interest as possible chemosensory G-proteins [30,31]. In *C. elegans* and insects, G_αo_ and G_αq_ subunits are involved in chemosensation with evidence of G_α_ protein compartmentalism within chemosensory neurons; G_αq_ subunits are localized to the dendrites, whereas only one G_αo_ subunit is exclusively located along the chemosensory neuron axon [28,32]. The G_αo_ subunit is required for potentiating signals initiated by G_αq_ subunits [32]. It is possible that ticks exhibit the same G_α_ protein compartmentalism as *C. elegans* and insects, establishing a two-step chemosensory signal transduction system. A two-step chemosensory signal transduction system would establish a quorum number of chemoreceptors that must be activated in order for signal transduction. It would also allow for multiple chemoreceptors, responding to either the same or variant stimuli, to simultaneously build an action potential, allowing for the integration of multiple chemoreceptor inputs into neuron signaling to the tick brain.

G_β_ subunits are important chemoreceptor signal modulators that can function as a dimer with G_γ_ subunits to regulate G_α_ subunits [28,33]. G_γ_ subunits are not required for the proper function of G_β_. In invertebrates, G_β_ subunits can be divided in three clades: β1, β2, and β5 [29]. Alignment and phylogenetic analysis of the putative G_β_ transcripts determined that both the Haller’s organ spf transcript (contig 24477) and the Illumina 1st leg transcript (contig 57459) encoded a divergent clade of G_β_ subunits (Figures S15 and S16); the Illumina 1st leg G_β_ transcript (contig 57459) was homologous to a G_β_ transcript identified in the Illumina 4th leg transcriptome (contig 36459). GO annotation and pathway identification of the putative Haller’s organ spf and Illumina 1st leg G_β_ transcripts revealed functional roles in GTPase regulation (GO:0043547). The GTPase activity of G_β_ subunits deactivates G_α_ subunits and instigates the re-association of all G-protein subunits with the GPCR [24,33]. G_β_ subunits are not well documented in Acari, and their roles in sensory signal modulation remain unclear. In *C. elegans* and *D. melanogaster*, two G_β_ subunits, β2 and β5 like, are expressed in chemosensory neurons and can function in part with or without G_γ_ subunits as negative regulators of G_α_ subunits [28,33].

BLASTx and BLASTn searches of the Illumina 1st leg and Haller’s organ specific transcriptomes did not find any G_γ_ subunits (*e-*value ≤ 1). BLASTx and BLASTn searches of the Illumina 4th leg and 454 1st leg transcriptomes did identify two putative G_γ_ transcripts. Furthermore, tBLASTn searches of the Illumina 1st and Illumina 4th leg BLAST databases did not clearly identify any additional G_γ_ subunit transcripts. *C. elegans* and insects have two G_γ_ subunits, a chemosensory type 1 and a non-chemosensory type 2 [33]. Phylogenetic analysis of the Illumina 4th leg (contig 3088) and 454 1st leg G_γ_ transcripts determined that neither were chemosensory (Figures S17 and S18). Since G_β_ subunits can form functional dimers with any protein that contains functional domains similar to the G_γ_ domain, the novel type of G_β_ subunit expressed exclusively in the Haller’s organ may function independently of G_γ_ subunits or with an unknown protein regulator, explaining their absence in the Haller’s organ, Illumina 1st leg transcriptome.

### 2.9. Secondary Messenger Proteins

Adenylate/Guanylate cyclases (AGCs) are enzymes that catalyze the formation of secondary messenger proteins, i.e., cyclic nucleotides (cNMPs). All chemosensory AGCs in insects and nematodes are transmembrane AGCs, though their classification into different subtypes has not been studied [6,27]. Two putative transcripts encoding AGCs were identified exclusively in the Haller’s organ spf transcriptome (contigs 37845 and 77721; Table 3). Alignments and phylogenetic analysis of the two putative AGCs transcripts (contigs 37845 and 77721) determined that both transcripts encoded transmembrane AGCs. GO annotation and pathway identification of the putative AGC transcripts (contigs 37845 and 77721) revealed functional roles in cNMP biosynthesis and signal transduction (GO:0006182, GO:0035556; Figures S19 and S20). Since G_αo_ subunits are associated with guanylate cyclase and G_αq_ subunits with adenylate cyclase, it is reasonable to assume that chemosensory AGCs also exhibit the same neuron compartmentalism as seen with G_α_ subunits [31]. The identification of both adenylate and guanylate cyclase exclusively in the Haller’s organ specific transcriptome further supports the presence of a two-step chemosensory signal transduction system in ticks.

### 2.10. Odorant Ion Channels

Cyclic nucleotide-gated ion channels (CNGs) control the cellular influx of Na^+^ and Ca^2+^ ions that leads to neuron depolarization and signal transduction. They are the ultimate targets of the cNMPs generated by AGCs in the GPCR signaling pathway [34]. CNGs are thought to function as hetero-oligomers, consisting of various combinations of α- and β-subunits [35]. One putative transcript encoding a CNG was identified exclusively in the Haller’s organ spf transcriptome (contig 82720; Table 3). A similar putative CNG transcript was also identified in the 454 1st leg transcriptome. Alignments and phylogenetic analysis of the putative CNG transcript (contig 82720) determined that it encoded an α-subunit, and GO annotation and pathway identification revealed functional roles in ion transmembrane transport (GO:0034220; Figure S21). No β-subunits were identified. In *C. elegans* and *D. melanogaster* a single type of CNG α-subunit is expressed in chemosensory neurons and is required for chemosensation. *C. elegans* also express a CNG β-subunit in their chemosensory neurons, but a β-subunit has yet to be identified in *D. melanogaster* antennae [34,36]. tBLASTn searches of the Illumina 1st and Illumina 4th leg BLAST databases did not identify any homologs to the *C. elegans* CNG β-subunit. The percent identities between the Haller’s organ spf CNG α-subunit (contig 82720) and the α-subunits of *C. elegans* and *D. melanogaster* were calculated to be 61% and 66%, respectively, having homologous cNMP binding functional domains (Figure S22). The percent identity between the *C. elegans* and *D. melanogaster* CNG α-subunits was calculated to be 30%. Additionally, OrthoDB determined that the Haller’s organ spf CNG α-subunit (contig 82720) and the *C. elegans* CNG α-subunit were transcribed from orthologous genes. Maybe ticks utilize one CNG to depolarize chemosensory neurons, and the identified α-subunit is the sole component of that CNG ion channel.

### 2.11. Putative Proteins Involved in Chemoreceptor Modulation

**Figure 4:**
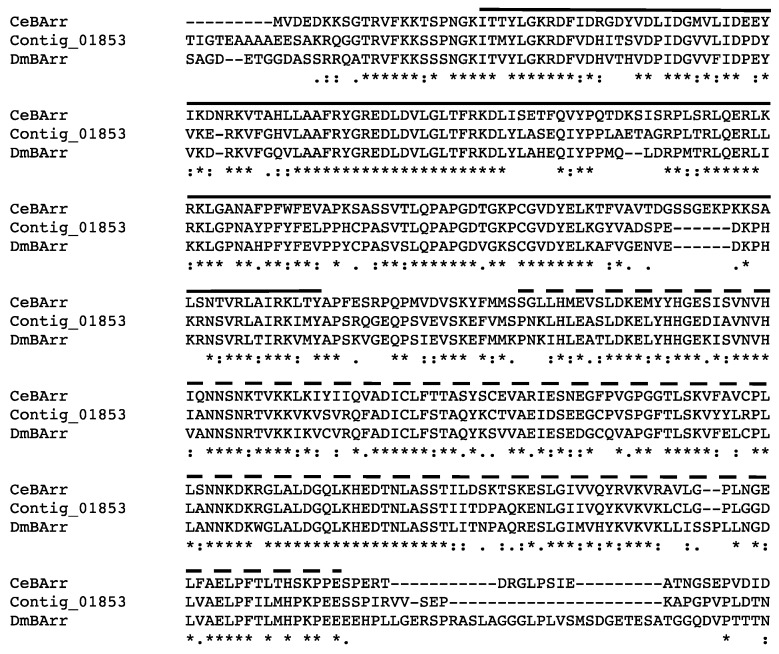
Multiple sequence alignment (Clustal Ω) of the deduced amino acid sequence for the putative β-arrestin (contig 1853) identified exclusively in the Haller’s organ spf transcriptome of unfed virgin adult male *Dermacentor variabilis* versus the *Caenorhabditis elegans* and *Drosophila melanogaster* β-arrestins (accession no. CCD67242.1 and AAF32365.1, respectively). Asterisks (*) denote conserved residues, colons (:) indicate conservation between groups of strongly similar properties scoring >0.5 in the Gonnet PAM 250 matrix, and periods (.) indicate conservation between groups of weakly similar properties scoring ≤0.5 on the Gonnet PAM 250 matrix. The solid black bar shows the arrestin amino terminal domain, and the dashed black bar shows the arrestin carboxyl terminal domain. The acronym consists of the first letter of genus and species (*Caenorhabditis elegans*, Ce; *Drosophila melanogaster*, Dm) followed by the protein name (BArr).

The Haller’s organ spf transcriptome was examined for transcripts encoding proteins involved in chemoreceptor signal termination and stimuli adaptation. One transcript encoding a putative arrestin was identified exclusively in the Haller’s organ spf transcriptome (contig 1853; Table 3). The same putative arrestin (contig 1853) was also identified in the 454 1st leg transcriptome. Arrestins are important protein modulators of GPCR signaling that bind activated GPCRs and sterically inhibit the signal cascade (illustration in Table 3). Arrestins can be classified into two clades, visual and non-visual otherwise known as β-arrestins [37,38]. Alignment and phylogenetic analysis determined that the Haller’s organ spf arrestin (contig 1853) encoded a putative cytosolic β-arrestin (Figure S23). GO annotation and pathway identification of the putative β-arrestin (contig 1853) revealed a functional role in environmental information processing and signal transduction (GO:007165). In *C. elegans* and *D. melanogaster*, a single cytosolic β-arrestin has been identified in chemosensory neurons that is required for the maintenance of GPCR sensitivity. The *C. elegans* and *D. melanogaster* β-arrestins promote the internalization of GPCRs, resulting in signal termination and adaptation to persistent stimuli [37,38]. The percent identity between the putative Haller’s organ spf β-arrestin (contig 1853) and the β-arrestins of *C. elegans* and *D. melanogaster* were calculated to be 61% and 68%, respectively, having homologous amino and carboxyl β-arrestin functional domains (Figure 4). The percent identity between the *C. elegans* and *D. melanogaster* β-arrestins was calculated to be 58%. Additionally, OrthoDB determined these three β-arrestins to be transcribed from orthologous genes. It is probable that ticks only possess one β-arrestin that functions to desensitize chemoreceptors and maintain GPCR sensitivity, and the identified transcript encodes that β-arrestin.

BLASTx and BLASTn searches of the Illumina 1st leg transcriptome identified several transcripts involved in chemoreceptor signal termination and chemosensory neuron recovery, unfortunately homologous transcripts were also present in the Illumina 4th leg transcriptome. This included a putative calmodulin transcript (contig 1632, 1st legs) and putative cNMP phosphodiesterase transcripts (contigs 1856, 4376, 4511, 20766, 1st legs). Similar calmodulin and cNMP phosphodiesterase transcripts were also identified in the 454 1st leg transcriptome. BLASTx and BLASTn searches of the Illumina 1st leg, Illumina 4th leg, and 454 transcriptomes did not identify any G-protein kinases (GPKs; *e-*value ≤ 1). GPKs are a specialized family of serine threonine kinases (STK), and several transcripts encoding putative STKs were present in all three transcriptomes. GPCRs are involved in a variety of signal transduction pathways in ticks, which may account for the lack of exclusivity of these enzyme modulators. In *C. elegans*, several confirmed chemoreceptor signal modulator enzymes are distributed throughout the animal’s nervous system, exhibiting similar functions in various types of neurons [6,37]. In ticks and *C. elegans*, there may be a limited diversity of GPCR modulator enzymes available, with the function of each enzyme determined by its location rather than its protein identity.

### 2.12. Odorant Degrading Enzymes

BLASTx and BLASTn searches of the 454 1st leg, Illumina 1st leg, Illumina 4th leg, and Haller’s organ spf transcriptomes identified several putative odorant degrading enzymes (ODEs) that were exclusive to the Haller’s organ spf transcriptome, including four transcripts for cytochrome P450 (P450 and CYP; contigs 1691, 6898, 14383 and 69591), two transcripts for glutathione S-transferase (GST; contigs 4931 and 12057), and one transcript for superoxide dismutase (SOD; contig 83534; Figures S24–S30, Table 4). Several putative epoxide hydrolases, esterases and methyltransferases were identified in the Illumina 1st leg transcriptome, but were also found in the Illumina 4th leg transcriptome and thus not specific to the 1st legs or Haller’s organ.

P450s are heme-thiolate membrane proteins that catalyze the oxidation of lipophilic molecules into reactive oxygen species (ROS) that are then degraded by GSTs or SODs [39,40]. GSTs and SODs metabolize ROSs into readily excretable hydrophilic products [40]. This two-phase odorant degradation metabolism has been detected specifically in insect antennal chemosensory sensilla [41], and this is the first documentation of the system associated with the forelegs and Haller’s organ of ticks.

**Figure 5:**
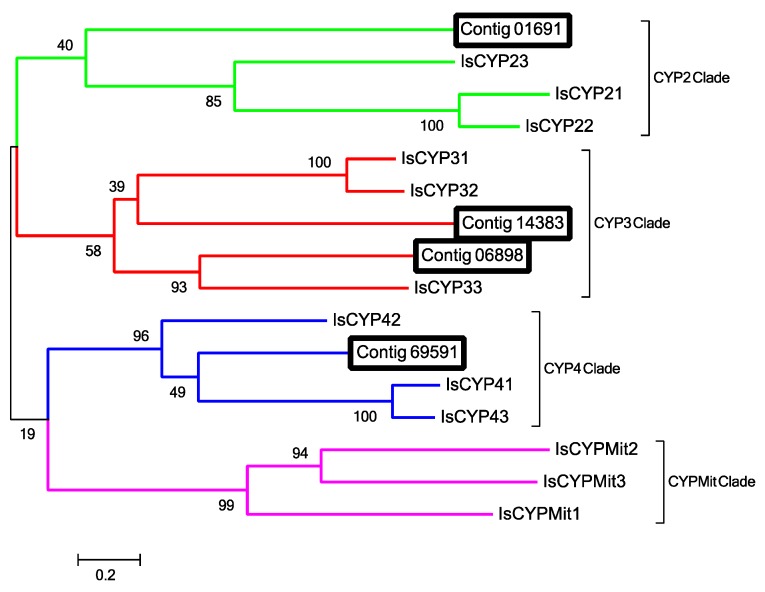
Phylogenetic relationship of transcripts putatively encoding cytochrome P450s (CYP or P450; contigs 1691, 6898, 14383 and 69591) identified in the Haller’s organ spf transcriptome of unfed, virgin adult male *Dermacentor variabilis* with P450s of known clan annotation from *Ixodes scapularis* (Is). The phylogenetic tree shows four P450 clades, each represented by a branch color as follows: green = CYP2 clade; red = CYP3 clade; blue = CYP4 clade; purple = mitochondrial CYP or CYPmit clade. Acronyms consist of the first letter of the genus and species (Is) followed by the protein name (CYP) and the number or abbreviation (mit = mitochondrial) of the associated clade. Putative P450s transcripts are boxed. The tree was constructed using Maximum likelihood phylogenetic analysis and bootstrapping set to 500 iterations. Branch values listed are bootstrap percentages (percent confidence), scale set to 20%. A comprehensive list of acronyms and associated GenBank accession numbers are listed in Appendix A.

P450s can be classified into four clades: CYP2, CYP3, CYP4 and mitochondrial CYP. Alignment and phylogenetic analysis of the 4 putative Haller’s organ spf P450 transcripts (contigs 1691, 6898, 14383 and 69591) determined that one transcript belongs to the CYP2 clade (contig 1691), two to CYP3 (contigs 14383 and 6898), and one to CYP4 (contig 69591; Figure 5). GO annotation and pathway identification of the Haller’s organ spf CYP transcripts (contigs 1691, 6898, 14383 and 69591 revealed functional roles in oxidation-reduction processes (GO:0055114). The majority of chemosensory specific P450s identified in insects belong to CYP2, CYP3 and CYP4 [42]. CYP2s, CYP3s and CYP4s are also commonly found in the gut and fat bodies of insects, though this does not preclude these enzymes from having chemosensory specific functions [43]. In insects, chemosensory CYP4s are primarily associated with the metabolism of odorants and pheromones, whereas CYP3s are linked to the metabolism of toxic and/or harmful odorant molecules [41]. CYP2s are orphan enzymes of unknown function. There is evidence of certain CYP2s being tissue specific in vertebrates, but this has yet to be examined in invertebrates [43]. The exclusive presence of these putative P450/CYP transcripts in the Haller’s organ spf transcriptome, as well as their functional annotations, suggests these enzymes function as ODEs in the foreleg of ticks, and possibly the Haller’s organ.

**Figure 6:**
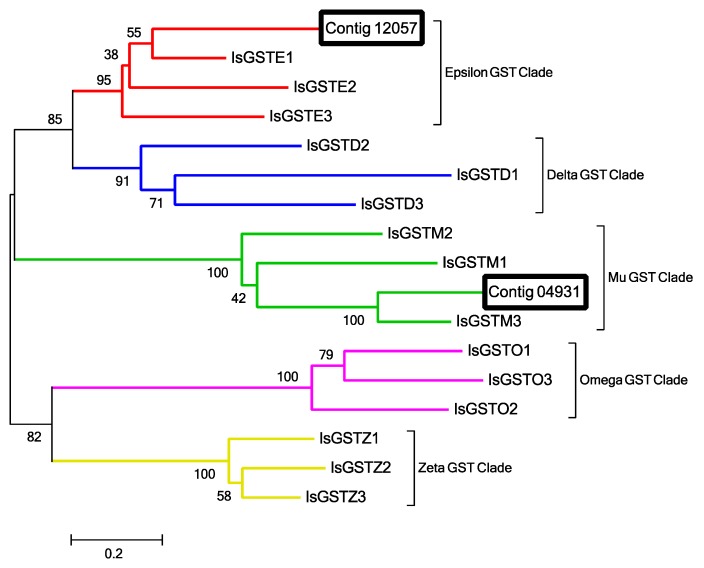
Phylogenetic relationship of transcripts putatively encoding glutathione S-transferases (GST; contigs 4931 and 12057) identified in the Haller’s organ spf transcriptome of unfed, virgin adult male *Dermacentor variabilis* with GSTs of known clade annotation from *Ixodes scapularis* (Is)*.* The phylogenetic tree shows 5 GST clades, each represented by a different branch color as follows: red = epsilon GST clade; blue = delta GST clade; green = mu GST clade; purple = omega GST clade; yellow = zeta GST clade. Acronyms consist of the first letter of the genus and species (Is), followed by protein name (GST) and the first letter of the associated clade (delta, D; epsilon, E; omega, O; mu, M; zeta, Z); acronyms are numbered 1, 2 and 3 to differentiate distinct sequences. Putative GST transcripts are boxed. The tree was constructed using Maximum likelihood phylogenetic analysis and bootstrapping set to 500 iterations. Branch values listed are bootstrap percentages (percent confidence), scale set to 20%. A comprehensive list of acronyms and associated GenBank accession numbers are listed in Appendix A.

GSTs are important antioxidant enzymes that degrade ROSs created in response to the CYP inactivation of pheromones and harmful chemical molecules [44]. GSTs can be classified into 7 clades: delta, epsilon, mu, omega, sigma, theta and zeta [45,46]. All GST clades except for sigma and theta have been documented in ticks [47]. Alignment and phylogenetic analysis of the two Haller’s organ spf putative GST transcripts (contigs 4931 and 12057) determined that one transcript encoded a cytosolic epsilon GST (contig 12057) and the second a cytosolic mu GST (contig 4931; Figure 6). GO annotation and pathway identification of the two Haller’s organ spf GST transcripts (contigs 4931 and 12057) revealed functional roles in the metabolism of xenobiotics following CYP oxidation (GO:008152). In insects, epsilon GSTs are highly expressed in antennal chemosensory sensilla and are associated with the degradation of pheromones and harmful odorant molecules [42,47,48]. Mu GSTs were previously recognized as vertebrate-specific, and associated with odorant degradation in nasal mucosa [49]. Several mu GSTs have been identified in multiple Acari species, though their roles in odorant degradation and general xenobiotic metabolism are still being studied [45].

SODs function in a similar manner to GSTs to prevent cellular oxidative damage from ROSs. Eukaryotic SODs can be classified into three clades, cytosolic, extracellular, and mitochondrial [50]. Alignment and phylogenetic analysis of the Haller’s organ spf SOD transcript (contig 83534) determined that it encoded a Cu/Zn binding cytosolic SOD (Figure S30). GO annotation of the putative SOD transcript (contig 83534) revealed a functional role in the removal of superoxide radicals (GO:0019430). Currently there are discrepancies in the reported functions of cytosolic SODs in insects and Acari, but it clear that SODs play a role in protecting chemosensory cells from ROSs damage [50].

ODEs in chemosensory sensilla protect chemosensory neurons from xenobiotic damage and odor desensitization. Efficient metabolism of odorant molecules, toxic or benign, may limit the duration of odorant activity and neural stimulation allowing for apt behavioral responses [44]. ODEs warrant further investigation as putative targets of novel tick control strategies.

### 2.13. Potential Developmental/Hormonal Regulation of Chemoreception in the Haller’s Organ

**Figure 7:**
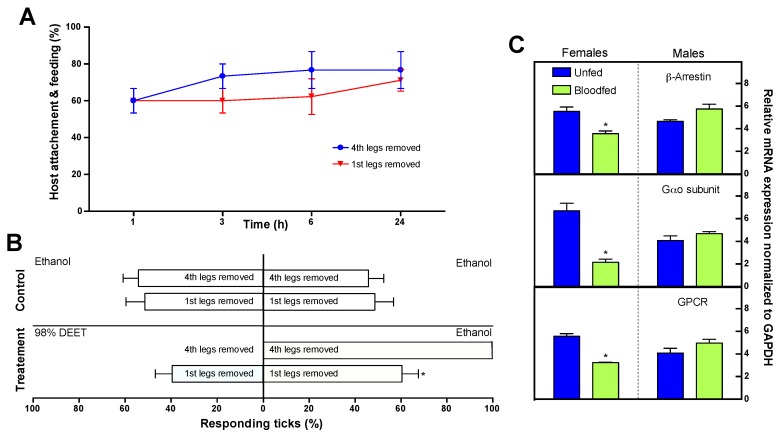
Observed behavior in response to the removal of either the 1st or 4th pairs of legs in unfed, virgin adult *D. variabilis*. (**A**) Time to rabbit (*Oryctolagus cuniculus*) and attachment by tick. Replicates of 4, with a mixed population of 15 female and 15 male ticks; (**B**) Petri dish assay two choice repellency assay. Replicates of 3 and 6 were conducted for the control and treatment, respectively, with a mixed population of 4 female and 4 male ticks; (**C**) Relative mRNA expression (Δ*C*t) of the chemosensory proteins β-arrestin, G-protein α_o_ subunit (Gαo) and G-protein coupled receptor (GPCR) in unfed versus fully blood fed adult female and male *D. variabilis* after normalization to expression levels of glyceraldehyde 3-phosphate dehydrogenase (GAPDH). Replicates of 5, using 2ng cDNA, 10 μL reactions. Statistical analyses were performed using an ANOVA (analysis of variance statistical model) and a Sidak’s multiple comparison test (* *p* < 0.05; error bars = ±1 SEM, standard error of the mean).

In male *D. variabilis,* blood feeding had no statistically significant effect on transcript levels in the 1st pairs of legs for GPCR (contig 83622), G_αo_ (contig 13937), or β-arrestin (contig 1853; Figure 7). This is not surprising since blood-fed males remain on the host, and are attracted to part-fed females for copulation. In females, the situation was different; mating and blood feeding to repletion resulted in a dramatic down-regulation of these same three chemosensory transcripts when compared to unfed virgin females. The GPCR expression decreased 5.0 fold (*t* = 5.677, dF = 12, *p* = 0.0001); G_αo_ expression decreased 10.0 fold (*t* = 7.598, dF = 12, *p* < 0.0001); and β-arrestin expression decreased 4.2 fold (*t* = 4.324, dF = 12, *p* = 0.0010). In *D. variabilis*, blood feeding to repletion is initiated in part-fed virgin females by insemination via the spermatophore. Mating and blood feeding also initiates the synthesis of the hormone 20-hydroxyecdysone; this hormone starts the process of egg development [1].

Blood feeding has been shown to impact chemosensory genes in other arthropods. For example, in adult female *A. gambiae* blood feeding resulted in the down-regulation of most antennal chemosensory gene transcripts with the exception of a subset of odorant receptors (AgORs) that were significantly up-regulated. These changes in chemosensory gene expression resulted in observable changes in odorant sensitivity and responsiveness. Blood-fed *A. gambiae* females were less receptive to host-associated attractants and more receptive to oviposition attractants [51]. In *D. variabilis* and other metastriate ticks, replete females lose their host seeking behavior, detach and drop from the host into the leaf litter where they oviposit their eggs and subsequently die [52]. It is not surprising that there is a down-regulation of chemosensory function at this time since all of the female tick’s energy is aimed at egg production and oviposition. Since increases in ecdysteroids triggered by blood feeding are responsible for host drop-off, initiation of egg develop and now associated with reduced gene expression of putative olfactory transcripts in the first leg containing the Haller’s organ, this might provide a new practical mechanism for repelling ticks and to reduce host seeking and biting using hormone mimics. Ecdysteroid mimics could potentially be used to turn off host detection in unfed females.

### 2.14. Role of the Haller’s Organ in Tick Repellency Versus Host Attachment

It has been well documented that the Haller’s organ is involved in host-seeking and mating behaviors; the general assumption has been that the Haller’s organ is also important in tick repellency and host biting and attachment, though this is not well documented [1]. In Petri dish bioassays, removal of the 1st pairs of legs, which includes the Haller’s organ, prevented both female and male unfed virgin adult *D. variabilis* from detecting the presence of a N,N-diethyl-meta-toluamide (DEET) treated surface (Figure 7). Without the 1st pairs of legs, DEET repellency was greatly reduced, and ticks were found on both the treated and control surfaces (control surfaces were treated with absolute ethanol; *F* = 22.430, dF = 34, *p* < 0.0001). In comparison, ticks were repelled from the DEET treated surface when the 4th pairs of legs were removed (*F* = 143.042, dF = 34, *p* < 0.0001). Since there was no evidence of GRs in the Haller’s organ (discussed earlier), the mechanism of this repellency in the Haller’s organ must be spatial (not contact) and involve olfaction. More bioassay work is needed to further validate this hypothesis. This discovery is significant in showing that the development of new tick repellents, must target the Haller’s organ and the odorant receptor system in this organ.

Also surprising, removal of the 1st pair of legs, which includes the Haller’s organ, had no significant impact on host biting or attachment when compared to ticks that had their 4th pair of legs removed at any of the time points examined, 1 h (*t* = 0.0, dF = 10, *p* = 1.00), 3 h (*t* = 1.112, dF = 10, *p* = 0.293), 6 h (*t* = 1.320, dF = 10, *p* = 0.216) or 24 h (*t* = 0.508, dF = 10, *p* = 0.623; Figure 7). We can only hypothesize that the pedipalps are used to regulate host biting and attachment, and that the Haller’s organ is required for host seeking, mating, and possibly strategic positioning on the host body. Our preliminary studies have shown that removal of the pedipalps prevents tick attachment in *D. variabilis.* The discovery of an organ, other than the Haller’s organ, that is critical in host attachment and feeding is exciting because it suggests a novel mechanism for the development of tick repellents that function to prevent tick biting and attachment. Future repellents could potentially be a mixture of compounds, which repel ticks from the host and also prevent host attachment.

## 3. Materials and Methods

### 3.1. Ticks

Adult *D. variabilis* were obtained from a highly-inbred laboratory colony of Dr. Daniel E. Sonenshine at Old Dominion University (Norfolk, VA, USA). The colony was started with field collected *D. variabilis* obtained from a single site near Richmond, VA, and reared for at least 30 generations by feeding larval and nymph stages on Norway rats, *Rattus norvegicus,* and adult stages on New Zealand white rabbits, *Oryctolagus cuniculus.* When not feeding, *D. variabilis* larvae, nymphs, adult females, and adult males were maintained in different containers at 23 ± 1° C, 94% humidity, and with a photoperiod of 16-hour light: 8-hour dark (dusk and dawn periods of 1 h each at the beginning and ending of the scotophase).

### 3.2. Ethics Statement

All applicable international, national, and/or institutional guidelines for the care and use of animals were followed to minimize pain and discomfort. All use of animals in this study was done under the protocol (#15-010, 20 February 2017) approved by the Old Dominion University Institutional Animal Care and Use Committee (Animal Welfare Assurance Number: A3172-01). This protocol is on file at the Office of Research, Old Dominion University, Norfolk, VA, USA.

### 3.3. RNA Extraction

Total RNAs extracted from the 1st and 4th legs of *D. variabilis* ticks were used in Illumina Hiseq sequencing and quantitative PCR (qPCR) studies. Ten batches of 100 1st legs and ten batches of 100 4th legs were dissected from unfed virgin adult males for use only in Illumina Hiseq sequencing. Five batches of 60 1st legs and five batches of 60 4th legs were dissected, separately, for each sex and blood feeding stage, unfed virgin adult males, unfed virgin adult females, fully blood-fed virgin adult males, and fully (replete) blood-fed mated adult females, for use in qPCR studies. In total, 20 batches of 60 1st legs and 20 batches of 60 4th legs were dissected for qPCR studies. Unfed virgin adult *D. variabilis* females and males used for dissections were 3–4 months post-molt, with females and males stored separately after molting. Fully blood-fed virgin adult males were fed on rabbit hosts (*O. cuniculus*) for 4–5 days and forcibly detached. Fully (replete) blood-fed mated adult females were 1–2 days post-drop off from the rabbit host (*O. cuniculus*). Dissections of each sex (female and male) and feeding stage (unfed and blood-fed) were performed on separate days to prevent cross-contamination. Additionally, all ticks were handled using gloves, and all dissection equipment washed thoroughly with glassware detergent and autoclaved between dissections. One tick specimen allotted two 1st legs and two 4th legs, removed at the femur. To maintain tissue viability, 1st and 4th leg dissections were conducted congruently. Leg dissections required two persons, one to dissect the 1st legs and one to dissect the 4th legs, with each person assigned a distinct set of sterile dissection tools. The 4th legs were dissected first to prevent cross-contamination of the hemolymph secreted from dissection sites. Dissections were performed during the day between 1100 and 1300 h under ambient (fluorescent) lighting at RT (24 ± 1 °C) and a relative humidity of 40%. Dissected 1st and 4th legs were collected into two distinct mortars each containing 3 mL liquid nitrogen (LN2; Airgas, Radnor, PA, USA), and two separate pestles used to grind the legs into fine particles. Once the LN2 evaporated, 350 µL of beta-mercaptoethanol in RLT lysis buffer (10 µL/1 mL; Qiagen, Valencia, CA, USA) was added to each mortar and the appropriate pestle used to homogenize the leg particles and lysis buffer. The mixtures were thawed, and an additional 350 µL of beta-mercaptoethanol in RLT lysis buffer (10 µL/1 mL; Qiagen, Valencia, CA, USA) was added to each mortar and homogenized using the appropriate pestle. All 700 µL of the lysis buffer, containing either the 1st or 4th leg particles, was transferred into a labeled 1.5 mL centrifuge tube and frozen overnight at −70 °C. Total RNA was extracted from the 1st and 4th leg particles in lysis buffer according to the manufacturer’s protocol using the RNeasy mini kit (Qiagen, Valencia, CA, USA). This process was repeated for each dissection batch, and the extracted RNAs kept separate to allow for biological replicates pertinent to qPCR studies. Total RNA concentrations and purities were measured using RNA pico chips in combination with the Agilent 2100 Bioanalyzer (Agilent Tech., Santa Clara, CA, USA). The decision for our research to focus on set part of the tick foreleg, confidently containing the Haller’s organ, that could be easily removed and processed for quality RNA in a reasonable amount of time, employing a reasonable amount of resources was determined based on the following dilemmas: the resulting small size of dissected leg parts, the small size of ticks in general, the inability to differentiate tick sex until the adult stage, the difficulty of rearing ticks to the adult stage because each stage requires feeding on a live animal host which can only be used once or twice per IACUC guidelines, the need for care to not cross contaminate tissue from the 1st legs with the 4th legs, heavy sclerotization of the 1st leg, foreleg where the Haller’s organ is located, lack of procedures to exclusively dissect the Haller’s organ from the leg, the lack of procedures to exclusively ablate the Haller’s organ, the requirement of a large amount of quality (not degraded) RNA to construct each transcriptome and provide biological replicates for qPCR developmental studies, and a desire to not overly stress the *D. variabilis* lab colony of Dr. Daniel E. Sonenshine which could result in decreased colony output, or total colony loss. Additionally,, our knowledge of chemoreception in the Haller’s organ is highly limited which would make it difficult to exclusively dissect in total the Haller’s organ from the tick foreleg, an area of the leg which is small and heavily sclerotized. Finally, the leg contains a great deal of muscle and apodemes since it is used for walking further making a specific Haller’s organ dissection challenging.

### 3.4. Illumina Hiseq Paired-End Sequencing

Deep sequencing utilizing Illumina RNA-Seq technology (Illumina, San Diego, CA, USA) was conducted at the University of North Carolina High Throughput Sequencing Facility. HiSeq paired-end sequencing was conducted using mRNAs extracted from the 1st and 4th legs of unfed virgin adult male *D. variabilis*. Poly(A) mRNAs were isolated, separately, from 2 µg of total RNA collected from the 1st legs, and 2 µg of total RNA collected from the 4th legs for library preparation and barcoding. The mRNAs were fragmented and cDNA synthesized, amplified, digested, and purified utilizing Illumina TruSeq chemistry protocols (Illumina, San Diego, CA, USA). The purified cDNA fragments generated were ligated with adaptor constructs creating the 1st and 4th leg cDNA libraries. In preparation for sequencing, the 1st and 4th leg cDNA libraries were barcoded with a 6 bp barcode (GCCAAT or CTTGTA) to distinguish the sequencing data generated from each library. The barcoded 1st and 4th leg cDNA libraries were hybridized onto one Illumina Hiseq flow cell for cBOT (Illumina, San Diego, CA, USA) cluster generation and sequencing (paired-end, 2 × 100 bp; total 200 cycles). Two raw data sets of sequencing outputs, one per library, were generated using the Illumina software assembler (Illumina, San Diego, CA, USA).

### 3.5. Illumina Bioinformatics

The 1st and 4th leg Illumina Hiseq data sets generated were cleaned using and quality trimmed (Q15) using the FASTX-Toolkit (http://hannonlab.cshl.edu/fastx_toolkit), and assembled de novo using the CLC pipeline assembler and scaffolder (Qiagen, Valencia, CA, USA) with k-mer set to “N”, minimum overlap set to 100 bases, seed length set to 5 kbp, and npairs set to 5. Putative functions and gene ontology (GO) annotations of contigs were predicted using the program Blast2GO (BioBam, Valencia, Spain) and the GenBank non-redundant protein database with an expect value (*e-*value) cut-off of <10. An in silico subtraction was then performed between the Illumina 1st and 4th leg contigs, for only those contigs with putative functions. Removal of the Illumina 1st leg contigs with identical counterparts, based on function and accession number, in the Illumina 4th leg transcriptome resulted in the identification of contigs exclusive to the 1st legs, creating the Haller’s organ spf transcriptome. The assumption is that since the Haller’s organ is exclusive to the 1st legs, contigs exclusive to the Illumina 1st leg transcriptome are associated with the Haller’s organ and chemosensation. BLASTx and BLASTn searches of the Illumina 1st leg, Illumina 4th leg and Haller’s organ spf transcriptome were conducted to identify chemosensory transcripts. BLASTx and BLASTp searches of all the tick sequence data contained in GenBank were also performed to identify chemosensory transcripts in other Ixodid species. Lastly, using the NCBI BLAST+ toolkit and the “makeBLASTdb” UNIX coding, the Illumina 1st leg transcriptome fasta file was used to create a BLASTable database. This Illumina 1st leg BLAST database was uploaded into the program Geneious (Biomatters, Auckland, New Zealand), and tBLASTn searches for tick, insect and nematode chemosensory messages (coding sequences) conducted to identify contig matches in the Illumina 1st leg BLAST database. The same procedure was used to create a BLASTable Illumina 4th leg BLAST database to rule out redundant messages. The functions and GO annotations of identified chemosensory transcripts were verified against the Uniprot knowledgebase using BLAST (EBI: European Bioinformatics Institute, Cambridgeshire, UK) and Argot^2^ (Annotation Retrieval of Genel Ontology Terms; FEM-IASMA: The Edmund Mach Research and Innovation Center at the Istituto Agrario di San Michele all’Adige, Trento, Italy) [53]. Protein families and domains were identified using the Pfam program and database (EBI, Cambridgeshire, UK). Alignments and trees were constructed using Clustal Omega (EBI, Cambridge, UK) and MAFFT (Multiple Alignment using Fast Fourier Transform) [54] with default E-INS settings, and visualized using Jalview v. 2.8.2 [55] and the Molecular Evolutionary Genetics Analysis program v.5.2.2 (MEGA; Biodesign Institute, Tempe, AZ, USA). Orthologous genes to *D. variabilis* chemosensory transcripts were predicted using OrthoDB [56].

### 3.6. 454 1st Leg Transcriptome

In addition to analyzing the Illumina 1st leg, Illumina 4th leg, and Haller’s organ spf transcriptomes to elucidate the chemosensory mechanism of ticks, permission was granted to conduct BLASTx searches for putative chemosensory transcripts of a combined female and male *D. variabilis* 1st leg transcriptome generated using 454 pyrosequencing. The 454 1st leg transcriptome was made using 1st legs dissected at the femur from unfed virgin adult female and male *D. variabilis* 3–4 months post-molt. Tissue processing, RNA extraction and 454 pyrosequencing were performed as described by Donohue et al. [57]. Dissected 1st legs were homogenized in TRI reagent (Sigma-Aldrich, St. Louis, MO, USA) and the total RNAs precipitated into a pellet that was rehydrated in 100 mM aurintricarboxylic acid to prevent dehydration. mRNA isolation from the precipitated total RNAs was performed using the Oligotex mRNA isolation kit (Qiagen, Valencia, CA, USA) according to the manufacturer’s protocol. A cDNA library was synthesized from the isolated mRNAs using the SMART (Switching Mechanism at 5’ End of RNA Template) cDNA library construction kit and protocol (Clontech, Mountain View, CA, USA). Purification of the cDNA library was performed using a PCR purification kit (Qiagen, Valencia, CA, USA) according to the manufacturer’s protocol. The purified cDNA library was prepared for pyrosequencing on the GS-FLX sequencer using GS-FLX Titanium library preparation and adaptors kits (Roche, Indianapolis, IN, USA). The generated raw sequencing dataset was assembled using the GS Assembler (Roche, Indianapolis, IN, USA) with default settings. Permission was granted to use the fasta file containing all the contigs for the 454 1st leg transcriptome. Putative functions and gene ontology (GO) annotations of contigs were predicted using the program Blast2Go (BioBam, Valencia, Spain) and the GenBank non-redundant protein (nr) database with an expect value (*e*-value) cut-off of <10. The functions and GO annotations of identified putative chemosensory transcripts were verified against the Uniprot knowledgebase using BLAST (BLAST; EBI, Cambridge, UK) and Argot^2^ (FEM-IASMA, Trento, Italy) [53]. BLASTx and BLASTn searches of the 454 1st leg transcriptome were conducted to identify putative chemosensory transcripts.

### 3.7. Quantitative Analysis of Putative Chemosensory Transcript Levels

Quantitative PCR (qPCR) experiments were conducted to determine the levels of putative chemosensory transcripts in unfed versus blood-fed adult female and male *D. variabilis.* qPCR experiments used total RNAs extracted from dissected 1st legs of unfed virgin adult females, unfed virgin adult males, fully blood-fed virgin adult males, and fully (replete) blood-fed mated females (see above, RNA Extraction). Total RNA was reverse transcribed into cDNA using the SuperScript^®^ III First-Strand Synthesis System (Life Technologies, Carlsbad, CA, USA) according to the manufacturer’s protocol. qPCR was performed using the C1000 Touch™ Thermal Cycler (Bio-Rad, Hercules, CA, USA) in combination with SsoFast™ EvaGreen^®^ Supermix technology and protocol (Bio-Rad, Hercules, CA, USA). Primer3Plus [58] was used to design primer pairs for the following messages: β-arrestin (contig 01853), G_αo_ subunit (contig 13937), and GPCR (contig 83622). Primer pairs were also designed for glyceraldehyde 3-phosphate dehydrogenase (GAPDH), used as a reference housekeeping message. The primer pairs are as follows; β-arrestin: forward (Fw) 5′-CTTCCAGTTCTGCCTTTTTGC-3′; reverse (Rv) 5′-TGCAAGACCATATCGCTGAG-3′; G_αo_ subunit: Fw 5′-AATACACAGGTGCCCAGGAG-3′; Rv 5′-CAAACTGGATGTTGGTCGTG-3′; GPCR: Fw 5′-TTCGGAAGACGTTCAAGGAT-3′; Rv 5′-CTCTCCGGTTACATCGAGGA-3′; GAPDH: Fw 5′-TGTCGGCAGCTTAGGTTATTCTT-3′; Rv 5′-GCCGATCTTCACGCTCATGT. Primers were validated in PCR studies and the subsequent products sequenced (Eton Bioscience Inc., Research Triangle Park, NC, USA) to confirm target identity prior to use in qPCR studies. qPCR experiments were conducted with 5 biological replicates for each sex and feeding stage. Each biological replicate was repeated twice for a total of 10 replicates for each sex and feeding stage. Expression data generated by qPCR experiments was normalized against GAPDH and analyzed using ANOVA (analysis of variance statistical model) and a Sidak’s multiple comparison test using Prism™ (GraphPad, La Jolla, CA, USA).

### 3.8. Repellency Bioassays

Petri dish assays were conducted to determine the relationship between the Haller’s organ and chemical avoidance behavior in *D. variabilis.* Unfed virgin adult male and female *D. variabilis* were tested in Petri dish assays against DEET following the removal of either the 1st or 4th legs. Petri dish assays were performed using black, glass Petri dishes (148 mm diam × 20 mm) and lids (150 mm diam × 20 mm) with filter paper used as the means of chemical delivery, testing 98% DEET (Sawyer, Safety Harbour, FL, USA) and absolute ethanol (control). Tests were conducted during the day between 1100 h and 1500 h at RT and a relative humidity of 40%. The use of black Petri dishes eliminated light contamination from ambient (fluorescent) lighting and created a dark testing environment. Adult ticks used in Petri dish assays were 3–4 months post-molt with females and males stored separately after molting. All ticks were handled using gloves and sterile, soft-tipped forceps cleaned with absolute ethanol. Additionally, all dissection equipment was washed thoroughly with glassware detergent and autoclaved between dissections to prevent contamination. The 1st or 4th legs of unfed virgin adult female and male *D. variabilis* were dissected at the femur under the same experimental conditions previously described. Ticks were permitted a recovery period of 24 h after leg dissections to confirm mobility and viability before use in assays. During the recovery period ticks were housed in an insectary maintained at 23 ± 1° C, 97% humidity and with a photoperiod of 16-hour light: 8-hour dark (dusk and dawn periods of 1 h each at the beginning and ending of the scotophase). In preparation for Petri dish assays, a piece of circular filter paper (6 cm^2^) was cut into two halves (3 cm^2^), and each half treated with 100 μL of either DEET or absolute ethanol (control). To allow for thorough chemical dispersal on the filter paper and solvent evaporation, all filter papers were chemically treated at least 30 min prior to use in assays. Control experiments employed two half pieces of filter paper (3 cm^2^) both treated with absolute ethanol. Treatment experiments employed two half pieces of filter paper (3 cm^2^) with one treated with DEET and the second with absolute ethanol. Sterile forceps were used to place the chemically treated filter papers into the bottom of the Petri dish. Filter papers treated with absolute ethanol were always handled first to prevent cross-contamination. After placement of the chemically treated filter papers into the Petri dish, sterile soft tipped forceps were used to introduce the test subjects to the experimental arena (Petri dish). Test subjects, 8 unfed virgin adult females and males (50:50) with either the 1st or 4th legs removed, were allowed 30 min to acclimate to experimental conditions prior to being placed on top of the chemically treated filter papers within the Petri dish. Each Petri dish assay was conducted for 30 min, with the location of the test subjects in reference to the chemically treated surfaces documented at 5, 10, 15, 20, 25 and 30 min. Due to the use of black, glass Petri dishes in experiments, the lids were removed briefly to catalog the location of ticks at each time point. Control experiments were conducted to document the normal behavior of test subjects in Petri dish assays, and to verify that there was no experimental bias associated with the Petri dish assay design or experimental conditions. Treatment experiments were conducted to observe the behavior of test subjects when exposed to DEET in Petri dish assays. In treatment experiments subjects arresting on the absolute ethanol treated (control) surface were recorded as exhibiting chemical avoidance behavior, while subjects arresting on the DEET treated surface were recorded as not exhibiting chemical avoidance behavior. *D. variabilis* unfed virgin adults with the 1st legs removed were tested in control experiments a total of 3 replicates, and in treatment experiments a total of 6 replicates. The same number of replicates were performed using *D. variabilis* unfed virgin adults with the 4th legs removed. Response data was analyzed by SAS (SAS, Cary, NC, USA) GLM (General Linear Models) and the Mixed-Procedure as well as ANOVA and a Sidak’s multiple comparison test using Prism™ (GraphPad, La Jolla, CA, USA).

### 3.9. Host Attachment/Feeding Bioassays

Host attachment/feeding assays were conducted to determine the relationship between the Haller’s organ and host biting, attachment and feeding behaviors in *D. variabilis.* Unfed virgin adult female and male *D. variabilis* were tested in attachment/feeding assays on live rabbit hosts (*O. cuniculus*) following the removal of either the 1st or 4th legs. Attachment/feeding assays were initiated at 900 h and run for 24 h at 20 ± 1° C and a relative humidity of 40%. The rabbit hosts were maintained at the Old Dominion University Animal Facility with a photoperiod of 14-h light: 10-h dark with dusk and dawn periods of 1 h each at the beginning and ending of each scotophase. Adult ticks used in attachment/feeding assays were 3–4 months post-molt with females and males stored separately after molting. All ticks were handled using gloves and sterile, soft-tipped forceps cleaned with absolute ethanol. Additionally, all dissection equipment was washed thoroughly with glassware detergent and autoclaved between dissections to prevent contamination. The 1st or 4th legs of unfed virgin adult female and male *D. variabilis* were dissected at the femur as previously described and under the same experimental conditions. Ticks were permitted a recovery period of 24 h after leg dissections to confirm mobility and viability before use in assays. During the recovery period, ticks were housed in an insectary maintained at 23 ± 1° C, 97% humidity and with a photoperiod of 16-hour light: 8-hour dark (dusk and dawn periods of 1 h each at the beginning and ending of the scotophase). Prior to conducting the attachment/feeding assays, ticks were allowed 30 min to acclimate to the testing environment. Test subjects, 30 unfed virgin adult females and males (50:50) with either the 1st or 4th legs removed, were placed into a plastic, dome-shaped feeding chamber (148 mm diam × 76 mm) that was adhered to the shaved flank of an animal using Vetbond (Santa Cruz Animal Health, Dallas, TX, USA), a veterinary topical adhesive. To protect the feeding chamber from animal manipulation, the animal was placed into a protective collar and a medical pet shirt (Medical Pet Shirts, Zuid-Holland, The Netherlands). The number of test subjects attaching and/or feeding on the animal host was documented at 1, 3, 6 and 24 h. In addition to the experimental time points, animals were also monitored twice daily by veterinary personal and had continuous access to food and water. During the attachment/feeding assays there were no indications that analgesics or anesthetics were needed to relieve animals of discomfort or stress. Assays were replicated 4 times, twice testing subjects with their 1st legs removed and twice testing subjects with their 4th legs removed. Two different animal hosts were used, and each allowed a recovery period of 3–4 weeks in between assays. Response data was collected and analyzed using ANOVA and a Sidak’s multiple comparison test using Prism™ (GraphPad, La Jolla, CA, USA).

## 4. Conclusions

Utilizing next-generation sequencing, we have generated the first Haller’s organ specific transcriptome by comparing new *D. variabilis* 1st (containing the Haller’s organ) versus 4th (where the Haller’s organ is absent) leg transcriptomes. Analyses suggest there are no known insect-like odorant binding proteins or vertebrate-like chemosensory lipocalins in ticks. Additionally, it appears that the Haller’s organ is only involved in olfaction and not gustation despite morphological evidence of the latter. The bioinformatic functional analysis of our transcriptomes were unsuccessful in supporting a mechanism of chemoreception like that in insects; instead, we found a GPCR signal cascade associated with the first pair of legs and the Haller’s organ. Each component of a putative olfactory GPCR signal cascade was identified using alignments, annotation and phylogenetic analyses. Additionally, the expressions of GPCR, G_αo_ and β-arrestin transcripts identified in the Haller’s organ specific transcriptome were documented by qPCR in unfed and blood-fed adult female and male *D. variabilis*. Blood feeding to repletion in adult females down-regulated the expression of all three chemosensory transcripts, consistent with what would be expected for olfactory elements in insects after blood feeding and a switch over from host attraction to tick drop off from the host in *D. variabilis.* This down-regulation did not occur in males which retain their attraction to the host. Also, our work represents the first documentation of the potential hormonal regulation of the chemosensory system in the Acari. Behavioral assays confirmed the role of the Haller’s organ in chemical avoidance, in addition to its known role in chemical attraction. Although, the Haller’s organ is essential for host-seeking, mating, and possibly strategic orientation on the host body, once ticks are present on the host, chemoreceptors present on the pedipalps are likely responsible for attachment and feeding.

The evidence presented suggests that ticks and maybe other acariforms utilize a chemosensory mechanism that is different from insects. This is a plausible conclusion given the drastic differences in sensilla types and number in the tick Haller’s organ versus that of the insect antennae [59] and the ancient divergence of ticks from insects. Similar chemosensory sensilla to ticks have been identified on the distal tips of the antenniform legs of whiptail scorpions, though this unique organization of chemosensilla appears to be exclusive to the arachnids. Recent evidence suggests the importance of the antenniform legs and olfaction in whiptail navigation [60]. Given, the dark nature of the ticks’ natural habitat, it is a reasonable conclusion that the Haller’s organ is ultimately a navigation organ functioning in either the presence/absence of a host or potential mate. The function of the Haller’s organ in tick spatial perception may also explain the conserved nature of the Haller’s organ sensilla, in both type and number; sensory input from too many stimuli could possibly impair tick spatial orientation, coordinately resulting in unsuccessful host-seeking and mating. These functional differences would explain the observed mechanistic differences of the tick Haller’s organ olfactory system in comparison to insects. Finally, the work presented here is just the first step in the development of an understanding of how chelicerates smell. In comparison to the amount of research conducted on insects, research on olfaction in ticks is in its infancy. What is apparent from this first study, we cannot assume tick are the same as insects. However, the work was limited since the transcriptomes did not contain the complete sequence of all transcripts, and by the current tick genomic data in general and lack of genomic sequencing in the American dog tick.

## Figures and Tables

**Figure 2:**
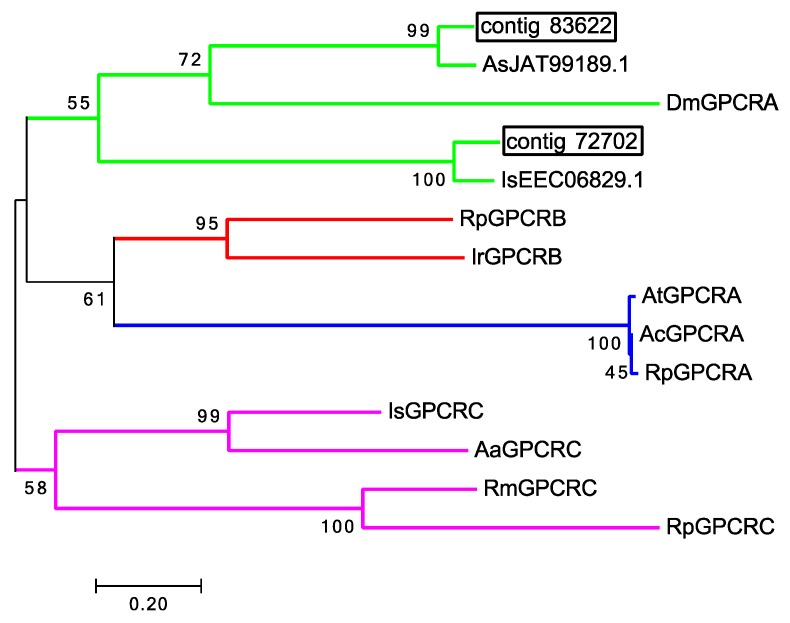
Phylogenetic relationship of transcripts putatively encoding G-protein coupled receptors (GPCR) identified exclusively in the Haller’s organ spf transcriptome (contigs 72702, 83622) of unfed, virgin adult male *Dermacentor variabilis* with their top GenBank BLAST hits (lowest expect value; Table 3) and GPCRs of known clade annotation from *Amblyomma americanum*, *Amblyomma cajennense*, *Amblyomma triste*, *Drosophila melanogaster*, *Ixodes ricinus*, *Ixodes scapularis*, *Rhipicephalus microplus*, and *Rhipicephalus pulchellus*. The phylogenetic tree shows four clades, each represented by the following branch colors: blue = clade A; purple = clade C; green = clade D; red = clade B. Acronyms are as follows: first letter of the genus and species (*Amblyomma americanum*, Aa; *Amblyomma cajennense*, Ac; *Amblyomma sculptum*, As; *Amblyomma triste*, At; *Drosophila* melanogaster, Dm; Ixodes *ricinus*, Ir; *Ixodes scapularis*, Is; *Rhipicephalus microplus*, Rm; *Rhipicephalus pulchellus*, Rp) followed by the protein name (GPCR) and the letter of the associated clade (A, B, and C) or GenBank accession number (JAT99,189.1, EEC06829.1). Putative GPCR transcripts are boxed. The tree was constructed using Maximum likelihood phylogenetic analysis and bootstrapping set to 500 iterations. Branch values listed are bootstrap percentages (percent confidence), scale set to 20%. A comprehensive list of acronyms and associated GenBank accession numbers are listed in Appendix A.

**Table 1:** The 50 most abundant transcripts from the unfed, virgin adult male *Dermacentor variabilis* Illumina 1st leg transcriptome.

Contig	Unique Reads	Accession No.	Best Match (Lowest *e*-Value) to UniprotKB Database	Organism	*e*-Value	Conserved Domain(s) ^a^	Putative Function
77381	1709234	EAW18142	Hypothetical protein	*Neosartorya fischeri*	6E+00	None	Unknown
04832	439971	EEC05627	Putative titin	*Ixodes scapularis*	00E+00	I-set	Myofibril scaffold
00022	410660	JAC21803	Putative troponin T skeletal muscle	*Rhipicephalus appendiculatus*	00E+00	Troponin	Skeletal muscle contraction
00073	278198	AAP79880	Actin	*Rhipicephalus microplus*	00E+00	None	Skeletal muscle contraction
00084	181667	AGH19694	Cytochrome oxidase subunit 1	*Dermacentor nitens*	00E+00	None	Aerobic metabolism
03069	167176	BAK26392	Ryanodine receptor	*Tetranychus urticae*	00E+00	Ins145_P3_rec, MIR	Intracellular cation transport
00151	137793	JAC30240	Uncharacterized protein	*Amblyomma triste*	00E+00	PDZ	Unknown
00696	137289	JAC22297	Putative titin	*Amblyomma cajennense*	00E+00	I-set, fn3	Myofibril scaffold
13661	118729	AAD23988	Beta-actin	*Tupaia belangeri*	2E-79	None	Cytoskeleton
00033	112728	ABB89211	Troponin I	*Rhipicephalus haemaphysaloides*	8E-49	None	Skeletal muscle contraction
00545	109190	XP_003739692	Uncharacterized protein	*Metaseiulus occidentalis*	6E-17	None	Unknown
18048	94602	CCW16442	Xanthine dehydrogenase	*Sphingobium japonicum*	1.2E+00	None	Amino acid metabolism
15573	94585	JAC34970	Putative myosin class II heavy chain	*Amblyomma triste*	7E-27	Myosin_head	Skeletal muscle contraction
04953	85346	JAA60593	Putative 24 kDa family member	*Rhipicephalus pulchellus*	4E-38	None	Unknown
27862	82021	JAB83083	Putative myosin class I heavy chain	*Ixodes ricinus*	3E-33	Myosin_head	Skeletal muscle contraction
00452	80428	ECC00524	Myosin heavy chain, skeletal muscle or cardiac muscle	*Ixodes scapularis*	3E-26	Myosin_N	Skeletal muscle contraction
00140	79270	JAA57343	Calcium transporting ATPase	*Rhipicephalus pulchellus*	00E+00	Cation ATPase_N	Organelle cation transporter
00619	78855	JAC31893	Putative actin binding cytoskeleton protein filamin	*Amblyomma triste*	00E+00	CH, filamin	Cytoskeleton
51899	78108	ETM32796	Uncharacterized protein	*Phytophthora parasitica*	8E+00	None	Unknown
08708	77869	ELU14970	Uncharacterized protein	*Capitella teleta*	1E+00	None	Unknown
02127	75107	JAC34859	Putative mitogen inducible protein product	*Amblyomma triste*	00E+00	FERM_M	Unknown
00589	74222	JAA62349	Uncharacterized protein	*Rhipicephalus pulchellus*	1E-112	None	Unknown
00036	72607	JAT93369	Putative myosin class I heavy chain	*Amblyomma aureolatum*	00E+00	None	Skeletal muscle contraction
00430	72504	JAC31335	Putative myosin regulatory light chain	*Amblyomma triste*	1E-133	EF-hand_6	Skeletal muscle contraction
00017	72240	AAD17324	Tropomyosin	*Rhipicephalus microplus*	00E+00	None	Skeletal muscle contraction
00471	66789	JAC94084	Putative endocytosis/signaling protein	*Ixodes ricinus*	00E+00	None	Endocytosis
73407	64871	XP_657842	Uncharacterized protein	*Emericella nidulans*	2E-10	None	Unknown
00449	62738	AEO34581	Uncharacterized protein	*Amblyomma maculatum*	00E+00	None	Unknown
00975	62079	JAB81899	Nucleolar GTP-binding protein	*Ixodes ricinus*	00E+00	NOGCT	Biogenesis of 60 s
00477	61091	JAC34490	Putative alanine-glyoxylate aminotransferase agt2	*Amblyomma triste*	00E+00	Aminotran_3	Amino acid metabolism
28761	60162	EEC00415	Putative ornithine decarboxylase	*Ixodes scapularis*	9E-126	Orn_Arg_deC_N, Orn_DAP_Arg_deC	Polyamine synthesis
07560	60258	JAC93558	Uncharacterized protein	*Ixodes ricinus*	1E-6	None	Unknown
00055	60152	JAC22069	Elongation factor 1-alpha	*Amblyomma cajennense*	00E+00	GTP_EFTU, GTP_EFTU_D2, GTP_EFTU_D3	Elongation and nuclear export
10926	60100	JAC29751	Putative alpha crystallins	*Amblyomma triste*	5E-65	HSP20	Stress response
01517	58463	EEC19998	Putative titin	*Ixodes scapularis*	00E+00	I-set, fn3	Myofibril scaffold
00690	55986	EEC04237	Putative cuticular protein	*Ixodes scapularis*	3E-69	CBM_14	Chitin metabolism
00310	55692	JAA60289	Eukaryotic translation initiation factor 4a2	*Rhipicephalus pulchellus*	00E+00	DEAD	Translation initiation
00960	55150	EEC14479	Putative stearoyl-CoA desaturase	*Ixodes scapularis*	00E+00	FA_desaturase	Iron binding/fatty acid metabolism
17549	54635	AAF81900	Beta-actin	*Aspergillus terreus*	2E-121	None	Cytoskeleton
00233	54395	JAC22444	Putative myosin class II heavy chain	*Amblyomma cajennense*	00E+00	None	Skeletal muscle contraction
00080	53133	JAC30913	Putative secreted protein	*Amblyomma triste*	4E-17	None	Unknown
00060	52729	JAC34970	Putative myosin class II heavy chain	*Amblyomma triste*	00E+00	Myosin_head	Skeletal muscle contraction
00355	52661	JAA61741	Putative eukaryotic translation initiation factor 4g2	*Rhipicephalus pulchellus*	00E+00	MIF4G	Translation initiation
43456	51562	EKN64830	Uncharacterized protein	*Bacillus azotoformans*	2E-1	None	Unknown
03024	50391	JAC22994	Putative secreted protein	*Amblyomma cajennense*	3E-9	None	Unknown
00517	50298	JAA60286	Putative amb caj-77 translation factor	*Rhipicephalus pulchellus*	00E+00	None	Translation
37644	50066	JAA56007	Putative der and -36 heat shock-related protein	*Rhipicephalus pulchellus*	9E-75	None	Stress response
00954	49578	JAC26141	Putative enolase	*Amblyomma parvum*	00E+00	Enolase_C, enolase_N	Glycolysis
02045	49068	JAA60432	Putative tick thioester protein	*Rhipicephalus pulchellus*	00E+00	A2M_comp, thiol-ester_cl	Fatty acid metabolism
02199	48695	JAA59973	Putative ATP	*Rhipicephalus pulchellus*	00E+00	ABC_tran, ABC_tran_2	ATPase activity

^a^ A2M_comp, A-macroglobulin complement component; ABC_tran, ATP-binding cassette transporter; ABC_tran_2, ATP-binding cassette transporter 2; Aminotran_3, aminotransferase class III; Cation ATPase_N, cation transporter/ATPase, amino-terminus; CBM_14, chitin binding peritrophin-A domain; CH, calponin homology domain; COX2, cytochrome C oxidase subunit II periplasmic domain; DEAD, DEAD/DEAH box helicase; EF-hand_6, EF-hand domain; Encolase_C, enolase carboxyl-terminus TIM barrel domain; Enolose_N, enolase amino-terminus; FA_desaturase, fatty acid desaturase; FERM_M, 4.1 protein, ezrin, radixin, moesin central domain; Filamin, filamin repeat domain; Fn3, fibronectin type III; GTP_EFTU, elongation factor Tu GTP binding; GTP_EFTU_D2, elongation factor Tu domain 2; GTP_EFTU_D3, elongation factor Tu carboxyl-terminus; HSP20, heat shock protein 20/alpha crystallin family; I-set, immunoglobulin intermediate-set; Ins145_P3_rec, inositol 1,4,5-triphosphate/ryanodine receptor; MIF4G, middle domain of eukaryotic initiation factor 4G; MIR, protein mannosyltransferase; Myosin_head, myosin head motor domain; Myosin_N, myosin amino-terminus; NOGCT, nucleolar GTP-binding protein carboxyl-terminus; Orn_Arg_deC_N, pyridoxal-dependent decarboxylase, pyridoxal binding domain amino-terminus; Orn_DAP_Arg_deC, pyridoxal-dependent decarboxylase carboxyl-terminus sheet domain; PDZ, post synaptic density protein; Thiol-ester_cl, alpha-macro-globulin thiol-ester bond forming region; Troponin, troponin.

**Table 2:** The 50 most abundant transcripts from the unfed, virgin adult male *Dermacentor variabilis* Illumina 4th leg transcriptome.

Contig	Unique Reads	Accession No.	Best Match (Lowest *e*-Value) to UniprotKB Database	Organism	*e*-Value	Conserved Domain(s) ^a^	Putative Function
20107	3060443	EEH50655	Uncharacterized protein	*Paracoccidioides brasiliensis*	6E-5	None	Unknown
03297	2068585	EDW75348	GK19734	*Drosophila willistoni*	5.9E+00	None	Unknown
29927	1571479	ABQ96857	Uncharacterized protein	*Haemaphysalis qinghaiensis*	5E-5	None	Unknown
06805	425907	EEC05627	Putative titin	*Ixodes scapularis*	00E+00	I-set	Myofibril scaffold
00086	344474	JAT93369	Putative myosin class I heavy chain	*Amblyomma aureolatum*	00E+00	None	Skeletal muscle contraction
00042	316355	JAC21803	Putative troponin T skeletal muscle	*Rhipicephalus appendiculatus*	00E+00	Troponin	Skeletal muscle contraction
13801	259741	EAU34989	TATA-box binding protein	*Aspergillus terreus*	6E-72	TBP	DNA binding/transcription
02472	200303	Not available	Uncharacterized protein	*Tetranychus urticae*	00E+00	Ins145_P3_rec, MIR	Intracellular cation transport
03579	194590	EEC12543	Uncharacterized protein	*Ixodes scapularis*	1E-28	None	Unknown
89009	165386	JAA56423	Uncharacterized protein	*Rhipicephalus pulchellus*	6E-58	None	Unknown
0194	150153	ABB89211	Putative troponin I	*Rhipicephalus haemaphysaloides*	2E-24	None	Skeletal muscle contraction
00250	149220	EPQ15604	Actin, cytoplasmic 1	*Myotis brandtii*	2E-120	None	Cytoskeleton component
00146	146196	AAP79880	Actin	*Rhipicephalus microplus*	00E+00	None	Skeletal muscle contraction
00082	133507	JAA57343	Calcium-transporting ATPase	*Rhipicephalus pulchellus*	00E+00	Cation ATPase_N	Organelle cation transporter
27571	132321	Unknown	Unknown	Unknown		None	Unknown
10067	131184	JAC31684	Arginyl tRNA protein transferase 1	*Amblyomma triste*	8E-162	None	Amino acid metabolism
00824	130575	JAC34993	Putative neural cell adhesion molecule L1	*Amblyomma triste*	00E+00	I-set, fn3	Cytoskeleton component
00023	126693	AGH19694	Cytochrome C oxidase	*Dermacentor nitens*	00E+00	None	Aerobic metabolism
00680	126367	JAB71798	Putative titin	*Ixodes ricinus*	3E-14	I-set	Myofibril scaffold
00424	124358	AGH19696	Cytochrome C oxidase subunit 2	*Dermacentor nitens*	6E-109	COX2	Aerobic metabolism
51752	122349	EEC14950	Uncharacterized protein	*Ixodes scapularis*	5E-14	None	Unknown
00375	118647	JAC94084	Putative endocytosis/signaling protein	*Ixodes ricinus*	00E+00	None	Endocytosis
00468	117492	JAC34970	Putative myosin class II heavy chain	*Amblyomma triste*	2E-175	Myosin_head	Skeletal muscle contraction
01684	112255	JAC29985	Uncharacterized protein	*Amblyomma triste*	1E-81	None	Unknown
02213	109709	JAC26409	Uncharacterized protein	*Amblyomma parvum*	00E+00	A2M_comp, thiol_ester_d	Unknown
17461	109466	EFG04290	Threonine dehydrogenase	*Streptomyces clavuligerus*	7.2E+00	None	Amino acid metabolism
01153	108934	Not available	Uncharacterized protein	*Xenopus tropicalis*	1.8E-1	None	Unknown
03566	108685	AAK73728	Uncharacterized protein	*Oryza* sp.	4.2+00	None	Unknown
00808	107982	EEC14479	Putative stearoyl-CoA desaturase	*Ixodes scapularis*	00E+00	FA_desaturase	Iron binding/fatty acid metabolism
15365	107585	JAB79130	Putative mitochondrial enolase	*Ixodes ricinus*	00E+00	MR_MLE, MR_MLE_C MR_MLE_N	Glycolysis
00182	105261	JAA59820	Uncharacterized protein	*Rhipicephalus pulchellus*	00E+00	Orn_Arg_deC_N, Orn_DAP_Arg_deC	Unknown
00543	105012	JAA64874	Uncharacterized protein	*Rhipicephalus pulchellus*	00E+00	NOGCT	Unknown
00574	103671	JAC34490	Putative alanine-glyoxylate aminotransferase agt2	*Amblyomma triste*	00E+00	Aminotran_3	Amino acid metabolism
00516	103502	JAC34859	Putative mitogen inducible protein product	*Amblyomma triste*	00E+00	FERM_M	Unknown
00006	100338	JAC22069	Elongation factor 1-alpha	*Amblyomma cajennense*	00E+00	GTP_EFTU, GTP_EFTU_D2, GTP_EFTU_D3	Elongation and nuclear export
15671	94282	BAC31766	Uncharacterized protein	*Mus musculus*	1E-50	None	Unknown
00912	93884	AEO34581	Uncharacterized protein	*Amblyomma maculatum*	00E+00	None	Unknown
04838	93041	KGG51869	Uncharacterized protein	*Microsporidia* sp.	2E-24	None	Unknown
02211	92976	ECC04237	Putative cuticular protein	*Ixodes scapularis*	3E-69	CBM_14	Chitin metabolism
02753	92396	JAA62349	Uncharacterized protein	*Rhipicephalus pulchellus*	8E-124	None	Unknown
00216	89994	JAA60289	Eukaryotic transcription initiation factor 4a2	*Rhipicephalus pulchellus*	00E+00	DEAD	Translation initiation
51752	89524	JAC25392	Putative wings up A	*Amblyomma parvum*	7E-32	None	Skeletal muscle contraction
07344	89354	JAA54211	Putative similar to chymotrypsin-elastase inhibitor ixodidin	*Rhipicephalus pulchellus*	6E-44	TIL	Immune response
00239	89239	AAD17324	Tropomyosin	*Rhipicephalus microplus*	00E+00	None	Skeletal muscle contraction
01350	88374	JAA61741	Putative eukaryotic translation initiation factor 4 gamma 2	*Rhipicephalus pulchellus*	00E+00	None	Translation initiation
74084	86871	Unknown	Unknown	Unknown	3E-63	None	Unknown
52616	85798	EEC03672	Putative gamma-glutamyltransferase	*Ixodes scapularis*	4E-23	None	Antioxidant metabolism
00355	85082	AAL75582	Ferritin	*Dermacentor variabilis*	1E-115	Ferritin	Iron homeostasis
00079	83955	JAC34970	Putative myosin class II heavy chain	*Amblyomma triste*	2E-175	Myosin_head	Skeletal muscle contraction
00043	83765	JAA55363	Acetyl Co-enzyme A oxidase	*Rhipicephalus pulchellus*	00E+00	Acyl_CoA_dh_1, Acyl_CoA_M, Acyl_CoA_ox_N	Metabolism

^a^ A2M_comp, A-macroglobulin complement component; Aminotran_3, aminotransferase class III; Acyl_CoA_dh_1, Acyl-CoA dehydrogenase carboxyl terminal domain; Acyl_CoA_M, Acyl-CoA dehydrogenase middle domain; Acyl_CoA_ox_N, Acyl-enzyme A oxidase amine terminal domain; Cation ATPase_N, cation transporter/ATPase, amino-terminus; CBM_14, chitin binding peritrophin-A domain; COX2, cytochrome C oxidase subunit II periplasmic domain; DEAD, DEAD/DEAH box helicase; FA_desaturase, fatty acid desaturase; FERM_M, 4.1 protein, ezrin, radixin, moesin central domain; Fn3, fibronectin type III; I-set, immunoglobulin intermediate-set; GTP_EFTU, elongation factor Tu GTP binding; GTP_EFTU_D2, elongation factor Tu domain 2; GTP_EFTU_D3, elongation factor Tu carboxyl-terminus; I-set, immunoglobulin intermediate-set; Ins145_P3_rec, inositol 1,4,5-triphosphate/ryanodine receptor; MIR, protein mannosyltransferase; MR_MLE, mandelate racemase/muconate lactonizing enzyme carboxyl terminus; MR_MLE_C, enolase carboxyl terminus; MR_MLE_N, mandelate racemase/muconate lactonizing enzyme amino terminus; Myosin_head, myosin head motor domain; NOGCT, nucleolar GTP-binding protein carboxyl-terminus; Orn_Arg_deC_N, pyridoxal-dependent decarboxylase, pyridoxal binding domain; Orn_DAP_Arg_deC, pyridoxal-dependent decarboxylase carboxyl terminal sheet domain; TBP, TATA-binding protein; Thiol_ester_d, alpha-macro-globulin thiol-ester bond-forming region; TIL, trypsin inhibitor cysteine rich domain; Troponin, troponin.

**Table 3:** Putative transcripts involved in chemoreceptor signal transduction identified exclusively in the Haller’s organ spf transcriptome of unfed, virgin adult male *Dermacentor variabilis* and their respective tick, nematode and insect matches with the lowest expect value (*e*-value). GenBank accession numbers are listed in Appendix A.

Chemoreceptor Signal Transduction & Stimuli Adaption	Protein	Contig (Length, bp)	Top Tick Hit ^a^ %ID & *e*-Value	Top *C. elegans* Hit ^b^ %ID & *e*-Value	Top Insect Hit ^c^ %ID & *e*-Value	Contig Conserved Domain(s) ^d^
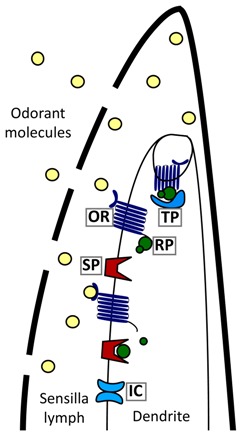	Lipocalin	84287 (279)	*A. triste* 48% & 12.00E-12	No match	No match	None found
	**OR**, Odorant receptor: G-protein coupled receptor	72702 (271)	*I. scapularis* 92% & 2.30E-30	*C. elegans* 25% & 2.00E-3	*F. arisanus* 35% & 8.50E-4	None found
	**RP**, Receptor protein: G_α_ protein	13937 (7348)	*R. pulchellus* 99% & 0.00E+00	*C. elegans* 89% & 0.00E+00	*H. saltator* 75% & 8.00E-166	G_alpha
	**RP**: G_β_ protein	24477 (354)	*I. ricinus* 75% & 4.70E-28	*C. elegans* 73% & 4.00E-25	*D. ponderosae* 94% & 3.40E-36	WD40
	**SP**, Secondary protein: Adenylate/guanylate cyclase	77721 (242)	*I. scapularis* 70% & 1.90E-30	No match	*D. plexippus* 58% & 4.60E-7	None found
		37845 (534)	*I. scapularis* 92% & 1.60E-105	*C. elegans* 36% & 2.00E-19	*Z. nevadensis* 61% & 6.20E-67	Guanylate_cyc, HNOBA
	**IC**, Ion channel: Cyclic nucleotide-gated ion channel	82720 (266)	*I. scapularis* 99% & 3.00E-51	*C. elegans* 61% & 8.00E-26	*A. echinatior* 93% & 3.00E-49	cNMP_binding
	**TP**, Terminator protein: β-Arrestin	1853 (3390)	*A. cajennense* 93% & 0.00E+00	*C. elegans* 63% & 5.80E-160	*L. hesperus* 83% & 0.00E+00	Arrestin_C, Arrestin_N

^a^ *Amblyomma cajennense* the Cayenne tick; *Amblyomma triste*, the hard tick; *Ixodes scapularis*, the blacklegged tick; *Ixodes ricinus*, the castor bean tick; *Rhipicephalus pulchellus*, the zebra tick; ^b^ *Caenorhabditis elegans*, the roundworm; ^c^ *Acromyrmex echinatior*, the Panama leaf cutting ant; *Danaus plexippus*, the Monarch butterfly; *Dendroctonus ponderosae*, the mountain pine bark beetle; *Fopius arisanus*, the solitary endoparasitoid; *Harpegnathos saltator*, the Indian jumping ant; *Lygus hesperus*, the Western tarnished plant bug; *Zootermopsis nevadensis*, the dampwood termite; ^d^ Arrestin_C, arrestin C-terminal domain; Arrestin_N, arrestin N-terminal domain; cNMP_binding, cyclic nucleotide binding domain; G_alpha, G-protein alpha subunit; Guanylate_cyc, adenylate and guanylate cyclase catalytic domain; HNOBA, heme no binding associated domain; WD40, β-transducin repeat domain.

**Table 4:** Putative transcripts involved in chemoreceptor signal modulation/termination and xenobiotic metabolism identified exclusively in the Haller’s organ spf transcriptome of unfed, virgin adult male *Dermacentor variabilis* and their respective tick, nematode and insect matches with the lowest expect value (*e*-value). GenBank accession numbers are listed in Appendix A.

Odorant Degradation Enzymes, ODE	Protein	Contig (Length, bp)	Top Tick Hit ^a^ %Identity & *e*-Value	Top *C. elegans* Hit ^b^ %Identity & *e*-Value	Top Insect Hit ^c^ %Identity & *e*-Value	Contig Conserved Domain(s) ^d^
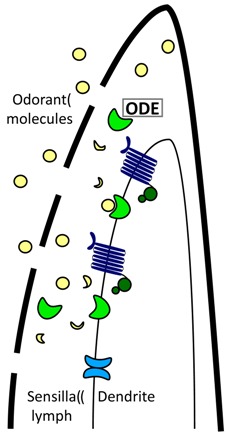	**ODE**, Cytochrome P450	69591 (575)	*I. scapularis* 57% & 1.30E-69	*C. elegans* 43% & 2.00E-39	*D. mojavensis* 42% & 1.30E-48	p450
		1691 (1001)	*R. pulchellus* 90% & 4.80E-79	*C. elegans* 30% & 7.00E-7	*C. biroi* 32% & 2.70E-9	p450
		6898 (1170)	*I. scapularis* 64% & 6.10E-123	*C. elegans* 33% & 3.00E-30	*L. bostrychophila* 40% & 3.0E-54	p450
		14383 (1167)	*A. triste* 69% & 2.30E-157	*C. elegans* 31% & 2.00E-27	*B. tabaci* 33% & 2.70E-41	p450
	**ODE**, Glutathione S-transferase	12057 (902)	*A. triste* 74% & 5.00E-110	*C. elegans* 31% & 6.00E-7	*A. glabripennis* 34% & 7.80E-38	GST_C_3, GST_N
		4931 (2250)	*A. triste* 88% & 3.30E-140	*C. elegans* 30% & 7.00E-18	*A. rosae* 31% & 1.00E-13	GST_C, GST_N
	**ODE**, Superoxide dismutase	83534 (332)	*R. pulchellus* 70%, 2.20E-38	*C. elegans* 55%, 3.00E-26	*P. cochleariae* 65% & 4.90E-36	Sod_Cu

^a^ *Amblyomma triste*, the hard tick; *Ixodes scapularis*, the blacklegged tick; *Rhipicephalus pulchellus*, the zebra tick; ^b^ *Caenorhabditis elegans*, the roundworm; ^c^ *Anoplophora glabripennis*, the Asian long-horned beetle; *Athalia rosae*, the turnip sawfly; *Bemisia tabaci*, the silverleaf whitefly; *Cerapachys biroi*, the clonal raider ant; *Drosophila mojavensis*, the fruit fly; *Liposcelis bostrychophila*, the booklouse; *Phaedon cochleariae*, the mustard beetle; ^d^ GST_C, glutathione S-transferase C-terminal domain; GST_C_3, glutathione S-transferase C-terminal domain; GST_N, glutathione S-transferase N-terminal domain; P450, cytochrome p450 domain; Sod_Cu, copper/zinc superoxide dismutase.

## Supplementary Materials

Supplementary materials can be found at www.mdpi.com/1422-0067/18/7/1563/s1.

## Appendix A

**Table A1:** Comprehensive list of the acronyms presented in alignments and phylogenetic trees, with the identifying species and GenBank accession numbers, and proteins used during tBLASTn searches of the Illumina 1st and 4th leg BLAST databases.

Acronym	Species	Accession No.
AaGA12/13	*Anopheles aquasalis*	JAA99692.1
AcGPCRA	*Amblyomma cajennense*	JAC21379.1
Adenlyate/guanylate cyclase: contig 37845	*Ixodes scapularis*	EEC13610.1
Adenlyate/guanylate cyclase: contig 37845	*Caenorhabditis elegans*	CCD67191.1
Adenlyate/guanylate cyclase: contig 37845	*Zootermopsis nevadensis*	KDR07447.1
Adenlyate/guanylate cyclase: contig 77721	*Ixodes scapularis*	EEC01411.1
Adenlyate/guanylate cyclase: contig 77721	*Danaus plexippus*	EH772322.1
AgGAI	*Anopheles gambiae*	ABA56308.1
AgGAO	*Anopheles gambiae*	AAW50310.1
AgGAQ	*Anopheles gambiae*	ABA56307.1
AgGAS	*Anopheles gambiae*	ABA56309.1
AgGPB1	*Aedes aegypti*	EAT33735.1
AgGPB2	*Aedes aegypti*	EAT40134.1
AgGPB5	*Aedes aegypti*	EAT38001.1
AtGPCRA	*Amblyomma triste*	JAC28848.1
BmGPB1	*Apis mellifera*	XM_006562979.1
BmGPB2	*Apis mellifera*	XM_624332.4
BmGPB5	*Apis mellifera*	XM_006568128.1
CeGA12/13	*Caenorhabditis elegans*	CCD61930.1
CeGAO	*Caenorhabditis elegans*	CAA96595.1
CeGAQ	*Caenorhabditis elegans*	CCD67955.1
CeGAS	*Caenorhabditis elegans*	CCD73235.2
CeGPB1	*Caenorhabditis elegans*	CAA35532.1
CeGPB5	*Caenorhabditis elegans*	CAA95824.1
Cyclic nucleotide-gated ion channel: contig 82720	*Ixodes scapularis*	EEC03664.1
Cyclic nucleotide-gated ion channel: contig 82720	*Caenorhabditis elegans*	CAB63418.2
Cyclic nucleotide-gated ion channel: contig 82720	*Acromyrmex echinatior*	EGI67156.1
Cytochrome P450: contig 01691	*Rhipicephalus pulchellus*	JAA56317.1
Cytochrome P450: contig 01691	*Caenorhabditis elegans*	CAB60436.1
Cytochrome P450: contig 01691	*Cerapachys biroi*	EZA58513.1
Cytochrome P450: contig 06898	*Ixodes scapularis*	EEC19065.1
Cytochrome P450: contig 06898	*Caenorhabditis elegans*	CAB07222.1
Cytochrome P450: contig 06898	*Liposcelis bostrychophila*	ABN80241.2
Cytochrome P450: contig 14383	*Amblyomma triste*	JAC34536.1
Cytochrome P450: contig 14383	*Caenorhabditis elegans*	CAA91268.1
Cytochrome P450: contig 14383	*Bemisia tabaci*	AEK21811.1
Cytochrome P450: contig 69591	*Ixodes scapularis*	EEC03681.1
Cytochrome P450: contig 69591	*Caenorhabditis elegans*	CCD68717.1
Cytochrome P450: contig 69591	*Drosophila mojavensis*	EDW16611.1
DmGA12/13	*Drosophila melanogaster*	AAA28569.1
DmGAI	*Drosophila melanogaster*	AAA28565.1
DmGAO	*Drosophila melanogaster*	AAA28586.1
DmGAQ	*Drosophila melanogaster*	AAA28460.1
DmGAS	*Drosophila melanogaster*	AAA28579.1
DmGPB1	*Drosophila melanogaster*	AAB59247.1
DmGPB2	*Drosophila melanogaster*	AAA73103.1
DmGPB5	*Drosophila melanogaster*	AAF46336.1
Dust mite antigen EST	*Amblyomma americanum*	JZ171890
Dust mite antigen EST	*Amblyomma americanum*	JZ173259
Glutathione-S transferase: contig 12057	*Amblyomma triste*	JAC32911.1
Glutathione-S transferase: contig 04931	*Amblyomma triste*	JAC32978.1
Glutathione-S transferase: contig 12057	*Caenorhabditis elegans*	CCD73730.1
Glutathione-S transferase: contig 04931	*Caenorhabditis elegans*	CCD62297.1
Glutathione-S transferase: contig 12057	*Anoplophora glabripennis*	JAB67323.1
Glutathione-S transferase: contig 04931	*Athalia rosae*	XP_012268124
GPCR: contig 72702	*Ixodes scapularis*	EEC06829.1
GPCR: contig 72702	*Caenorhabditis elegans*	CCF23345.1
GPCR: contig 72702	*Fopius arisanus*	JAG78630.1
GPCR: contig 83622	*Amblyomma sculptum*	JAT99189.1
GPCR: contig 83622	*Caenorhabditis elegans*	CCD63420.1
GPCR: contig 83622	*Drosophila melanogaster*	AAF49949.2
Gα protein: contig 13937	*Rhipicephalus pulchellus*	JAA58325.1
Gα protein: contig 13937	*Caenorhabditis elegans*	CAA96595.1
Gα protein: contig 13937	*Harpegnathos saltator*	EFN90078.1
Gβ protein: contig 24477	*Ixodes ricinus*	JAB79904.1
Gβ protein: contig 24477	*Caenorhabditis elegans*	CAA93514.1
Gβ protein: contig 24477	*Dendroctonus ponderosae*	AEE61583.1
Ionotropic receptor 100a	*Anopheles gambiae*	EAA06777.4
Ionotropic receptor 141	*Anopheles gambiae*	EGK96642.1
Ionotropic receptor 21a	*Drosophila melanogaster*	AAF51569.2
Ionotropic receptor 25a	*Drosophila melanogaster*	AGB92585.1
Ionotropic receptor 25a	*Anopheles gambiae*	EAA13931.5
Ionotropic receptor 40a	*Drosophila melanogaster*	ACD99552.1
Ionotropic receptor 40a	*Anopheles gambiae*	EAA13593.5
Ionotropic receptor 68a	*Anopheles gambiae*	EAA12883.4
Ionotropic receptor 8a	*Anopheles gambiae*	EAA07123.4
Ionotropic receptor 93a	*Anopheles gambiae*	EAA06694.5
IR Ionotropic receptor 93a	*Drosophila melanogaster*	AAN71127.1
IrGPCRB	*Ixodes ricinus*	JAB73169.1
IrGPCRD1	*Ixodes scapularis*	JAB74122.1
IrGPCRD2	*Ixodes scapularis*	JAB69073.1
IsCYP21	*Ixodes scapularis*	EEC15754.1
IsCYP22	*Ixodes scapularis*	EEC15758.1
IsCYP23	*Ixodes scapularis*	EEC02779.1
IsCYP31	*Ixodes scapularis*	EEC08584.1
IsCYP32	*Ixodes scapularis*	EEC17346.1
IsCYP33	*Ixodes scapularis*	EEC05868.1
IsCYP41	*Ixodes scapularis*	EEC15441.1
IsCYP42	*Ixodes scapularis*	EEC16313.1
IsCYP43	*Ixodes scapularis*	ECC15440.1
IsCYPMit1	*Ixodes scapularis*	EEC14261.1
IsCYPMit2	*Ixodes scapularis*	EEC01694.1
IsCYPMit3	*Ixodes scapularis*	EEC13863.1
IsGPCRD	*Ixodes ricinus*	EEC20264.1
IsGSTD1	*Ixodes scapularis*	EEC06462.1
IsGSTD2	*Ixodes scapularis*	EEC09120.1
IsGSTD3	*Ixodes scapularis*	EEC15339.1
IsGSTE1	*Ixodes scapularis*	EEC09121.1
IsGSTE2	*Ixodes scapularis*	EEC09122.1
IsGSTE3	*Ixodes scapularis*	EEC10833.1
IsGSTM1	*Ixodes scapularis*	EEC05606.1
IsGSTM2	*Ixodes scapularis*	EEC05607.1
IsGSTM3	*Ixodes scapularis*	EEC12982.1
IsGSTO1	*Ixodes scapularis*	EEC06527.1
IsGSTO2	*Ixodes scapularis*	EEC08493.1
IsGSTO3	*Ixodes scapularis*	EEC08494.1
IsGSTZ1	*Ixodes scapularis*	EEC03386.1
IsGSTZ2	*Ixodes scapularis*	EEC17074.1
IsGSTZ3	*Ixodes scapularis*	EEC20054.1
Lipocalin: contig 84287	*Amblyomma triste*	JAC30054.1
LUSH	*Drosophila melanogaster*	AAB58940.1
Microplusin EST	*Amblyomma americanum*	JZ182754
Microplusin EST	*Amblyomma americanum*	BF008203
Microplusin EST	*Amblyomma americanum*	JZ181776
Neto-like EST	*Amblyomma americanum*	JZ169554
Niemann-Pick C2 EST	*Amblyomma americanum*	JZ171494
Niemann-Pick C2 EST	*Amblyomma americanum*	BF008372
OBP	*Aedes aegypti*	AAA29347.1
OBP19a	*Drosophila melanogaster*	AAF50910.2
OBP19d	*Drosophila melanogaster*	AAC46475.1
OBP28a	*Drosophila melanogaster*	AAC46478.1
OBP45	*Aedes aegypti*	EAT37277.1
OBP56a	*Drosophila melanogaster*	AAL49345.1
OBP56d	*Drosophila melanogaster*	AAF57520.1
OBP56h	*Drosophila melanogaster*	AAY55796.1
OBP57a	*Drosophila melanogaster*	AAM69286.1
OBP57b	*Drosophila melanogaster*	AAM69285.1
OBP57c	*Drosophila melanogaster*	AAF57467.1
OBP57d	*Drosophila melanogaster*	AAM69287.1
OBP57e	*Drosophila melanogaster*	AAF57460.2
OBP66	*Anopheles gambiae*	EAU77642.1
OBP67	*Anopheles gambiae*	EAL42143.2
OBP68	*Anopheles gambiae*	EAU77839.1
OBP69	*Anopheles gambiae*	EGK96890.1
OBP69a	*Drosophila melanogaster*	AAC46474.1
OBP70	*Anopheles gambiae*	EAA11535.2
OBP71	*Anopheles gambiae*	EAL42442.3
OBP72	*Anopheles gambiae*	EDO64833.1
OBP83a	*Aedes aegypti*	CAF02084.1
OBP83a	*Drosophila melanogaster*	AAC46476.1
OBP84a	*Drosophila melanogaster*	AAC46477.1
OBP99a	*Drosophila melanogaster*	AAM29630.1
OBP99b	*Drosophila melanogaster*	AAM50904.1
OR83b	*Anopheles gambiae*	AAR14938.1
OR83b	*Drosophila melanogaster*	AAT71306.1
PBP6	*Drosophila melanogaster*	AAA21356.1
RmGPCRC	*Rhipicephalus microplus*	ACV07675.1
RnOBP1	*Rattus norvegicus*	AAA41736.2
RnOBP1	*Rattus norvegicus*	ABG24236.1
RnOBP1	*Rattus norvegicus*	AAH86942.1
RpGPCRA	*Rhipicephalus pulchellus*	JAA57151.1
RpGPCRB	*Rhipicephalus pulchellus*	JAA63917.1
RpGPCRC	*Rhipicephalus pulchellus*	JAA54919.1
Str-2	*Caenorhabditis elegans*	AAF23508.1
Superoxide dismutase	*Rhipicephalus pulchellus*	JAA58838.1
Superoxide dismutase	*Caenorhabditis elegans*	CAR97839.1
Superoxide dismutase	*Phaedon cochleariae*	AEY77316.1
TRPA	*Ixodes scapularis*	EEC13968.1
TRPA1	*Heliocoverpa armigera*	AHV83756.1
TRPA5_M4	*Cydia pomonella*	AOR16348.1
TRPA5_M43	*Cydia pomonella*	AOR16350.1
β-Arrestin: contig 01853	*Amblyomma cajennense*	JAC21990.1
β-Arrestin: contig 01853	*Caenorhabditis elegans*	CCD67242.1
β-Arrestin: contig 01853	*Lygus hesperus*	JAG24601.1

## References

[1] Sonenshine D.E., Roe R.M. (2014). Biology of Ticks.

[2] Lin L., Decker C.F. (2012). Rocky Mountain spotted fever. Dis. Mon..

[3] Liu X.Y., Bonnet S.I. (2014). Hard tick factors implicated in pathogen transmission. PLoS Neglect. Trop. Dis..

[4] Brunner J.L., Cheney L., Keesing F., Killilea M., Logiudice K., Previtali A., Ostfeld R.S. (2011). Molting success of *Ixodes scapularis* varies among individual blood meal hosts and species. J. Med. Entomol..

[5] Leonovich S.A. (2006). Sensory Systems of Parasitic Ticks and Mites.

[6] Hart A.C., Chao M.Y. (2010). From odors to behavior in *Caenorhabditis*. The Neurobiology of Olfaction.

[7] Nakagawa T., Vosshall L.B. (2009). Controversy and consensus: Non-canonical signaling mechanism in the insect olfactory system. Curr. Opin. Neurobiol..

[8] Shoura S.M. (1989). Fine structure of muscles in the tick *Hyalomma* (*Hyalomma*) *dromedarii* (Ixodoidea: Ixodidae). Exp. Appl. Acarol..

[9] Nave R., Weber K. (1990). A myofibrillar protein of insect muscle related to vertebrate titin connects Z band and A band: Purification and molecular characterization of invertebrate mini-titin. J. Cell Sci..

[10] Gao J., Luo J., Fan R., Guan G., Fingerle V., Sugimoto C., Inoue N., Yin H. (2008). Cloning and characterization of a cDNA clone encoding troponin T from tick *Haemaphysalis qinghaiensis* (Acari: Ixodidae). Comp. Biochem. Phys. B.

[11] Sonenshine D.E., Roe R.M. (2014). Biology of Ticks.

[12] Bissinger B.W., Donohue K.V., Khalil S.M., Grozinger C.M., Sonenshine D.E., Roe R.M. (2011). Synganglion transcriptome and developmental global gene expression in adult females of the American dog tick *Dermacentor variabilis* (Acari: Ixodidae). PLoS ONE.

[13] Gulia-Nuss M., Nuss A.B., Meyer J.M., Sonenshine D.E., Roe R.M., Waterhouse R.M., Sattelle D.B., de la Fuente J., Ribeiro J.M., Megy K. (2016). Genoimc insights into the *Ixodes scapularis* tick vector of Lyme Disease. Nature Commun..

[14] Renthal R., Manghnani L., Bernal S., Qu Y., Griffith W.P., Lohmeyer K., Guerrero F.D., Borges L.M., Pérez de León A. (2016). The chemosensory appendage proteome of *Amblyomma americanum* (Acari: Ixodidae) reveals putative odorant-binding and other chemoreception-related proteins. Insect Sci..

[15] Nespoulous C., Briand L., Delage M.M., Tran V., Pernollet J.C. (2004). Odorant binding and conformation changes of a rat odorant-binding protein. Chem. Senses.

[16] Egekwu N., Sonenshine D.E., Bissinger B.W., Roe R.M. (2014). Transcriptome of the female synganglion of the Black-legged tick *Ixodes scapularis* (Acari: Ixodidae) with comparison between Illumina and 454 systems. PLoS ONE.

[17] Liu C., Pitts R.J., Bohbot J.D., Jones P.L., Wang G., Zwiebel J. (2010). Distinct olfactory signaling mechanism in the malaria vector mosquito *Anopheles gambiae*. PLoS Biol..

[18] Chen C., Buhl E., Xu M., Croset V., Ress J.S., Lilley K.S., Benton R., Hodge J.J., Stanewsky R. (2015). *Drosophila* ionotropic receptor 25a mediates circadian clock resetting by temperature. Nature.

[19] Fowler M.A., Montell C. (2014). *Drosophila* TRP channels and animal behavior. Life Sci..

[20] Cattaneo A.M., Bengtsson J.M., Montagné N., Jacquin-Joly E., Rota-Stabelli O., Salvagnin U., Bassoli A., Witzgall P., Anfora G. (2016). TRPA5, an Ankyrin subfamily insect TRP channel, is expressed in antennae of *Cydia pomonella* (Lepidoptera: Tortricidae) in multiple splice variants. J. Insect Sci..

[21] Wei J.J., Fu T., Yang T., Liu Y., Wang G.R. A TRPA1 channel that senses thermal stimulus and irritating chemicals in *Heliocoverpa armigera*. Insect Mol. Biol..

[22] Hill C.A. (2002). G Protein-coupled receptors in *Anopheles gambiae*. Science.

[23] Kaupp U.B. (2010). Olfactory signaling in vertebrates and insects: Differences and commonalities. Nat. Rev. Neurosci..

[24] Broeck J.V. (2001). Insect G protein-coupled receptors and signal transduction. Arch. Insect Biochem..

[25] Munoz S., Guerrero F.D., Kellogg A., Heekin A.M., Leung M. (2017). Bioinformatic prediction of G protein- coupled recptor encoding sequences from the transcriptome of the foreleg, including the Haller’ organ of the cattle tick, *Rhipicephalus australis*. PLoS ONE.

[26] Merrill C.E., Riesgo-Escovar J., Pitts R.J., Kafatos F.C., Carlson J.R., Zwiebel L.J. (2001). Visual arrestins in olfactory pathways of *Drosophila* and the malaria vector mosquito *Anopheles gambiae*. Proc. Natl. Acad. Sci. USA.

[27] Boto T., Gomez-Diaz C., Alcorta E. (2010). Expression analysis of the 3 G-protein subunits Gα, Gβ, and Gγ in the olfactory receptor organs of adult *Drosophila melanogaster*. Chem. Sens..

[28] Tu H., Qin Y. (2014). Cloning and expression analysis of G-protein G_αq_ subunit and Gβ1 subunit from *Bemisia tabaci* Gennadius (Homoptera: Aleyrodidae). Arch. Insect Biochem..

[29] Hull J.J., Wan M. (2015). Molecular cloning and characterization of Gα proteins from the western tarnished plant bug, *Lygus Hesperus*. Insects.

[30] Yau D.M., Yokoyama N., Goshima Y., Siddiqui Z.K., Siddiqui S.S., Kozasa T. (2003). Identification and molecular characterization of the Gα12-ρ guanine nucleotide exchange factor pathway in *Caenorhabditis elegans*. Proc. Natl. Acad. Sci. USA.

[31] Rützler M., Lu T., Zwiebel L.J. (2006). G_α_ encoding gene family of the malaria vector mosquito *Anopheles gambiae*: Expression analysis and immunolocalization of AG_αq_ and AG_αo_ in female antennae. J. Comp. Neurol..

[32] Chatterjee A., Roman G., Hardin P.E. (2009). G_o_ contributes to olfactory reception in *Drosophila melanogaster*. BMC Physiol..

[33] Khan S.M., Sleno R., Gora S., Zylbergold P., Laverdure J.P., Labbé J.C., Miller G.J., Hérbert T.E. (2013). The expanding roles of Gβγ subunits in G protein-coupled receptor signaling and drug action. Pharmacol. Rev..

[34] Komatsu H., Mori I., Rhee J. (1996). Mutations in a cyclic nucleotide-gated channel lead to abnormal thermosensation and chemosensation in *C. elegans*. Neuron.

[35] Finn J.T., Grunwald M.E., Yau K. (1996). Cyclic nucleotide-gated ion channels: An extended family with diverse functions. Annu. Rev. Physiol..

[36] Baumann A., Frings S., Godde M., Seifert R., Kaupp U.B. (1994). Primary structure and functional expression of a *Drosophila* cyclic nucleotide-gated channel present in eyes and antennae. EMBO J..

[37] Fukuto H.S., Ferkey D.M., Apicella A.J., Lans H., Sharmeen T., Chen W., Lefkowitz R.J., Jansen G., Schafer W.R., Hart A.C. (2004). G protein-coupled receptor kinase function is essential for chemosensation in *C. elegans*. Neuron.

[38] Ge H., Krishnan P., Liu L., Krishnan B., Davis R.L., Hardin P.E., Roman G. (2006). A *Drosophila* nonvisual arrestin is required for the maintenance of olfactory sensitivity. Chem. Senses.

[39] Linblom T.H., Dodd A.K. (2006). Xenobiotic detoxification in the nematode *Caenorhabditis elegans*. J. Exp. Zool..

[40] Liu B., Jiang G., Zhang Y., Li J., Li X., Yue J., Chen F., Liu H., Li H., Zhu S., Wang J., Ran C. (2011). Analysis of transcriptome differences between resistant and susceptible strains of the citrus red mite *Panonychus citri* (Acari: Tetranychidae). PLoS ONE.

[41] Feyereisen R., Gilbert L.I. (2012). Insect *CYP* genes and P450 enzymes. Insect Molecular Biology and Biochemistry.

[42] Younus F., Chertemps T., Pearce S.L., Pandey G., Bozzolan F., Coppin C.W., Russell R.J., Maïbèche-Coisne M., Oakeshott J.G. (2014). Identification of candidate odorant degrading gene/enzyme systems in the antennal transcriptome of *Drosophila melanogaster*. Insect Biochem. Mol..

[43] Karlgren M., Miura S., Ingelman-Sundberg M. (2005). Novel extrahepactic cytochrome P450s. Toxicol. Appl. Pharm..

[44] Rogers M.E., Jani M.K., Vogt R.G. (1999). An olfactory-specific glutathione S-transferase in the sphinx moth *Manduca sexta*. J. Exp. Biol..

[45] Liao C.Y., Zhang K., Niu J.Z., Ding T.B., Zhong R., Xia W.K., Dou W., Wang J.J. (2013). Identification and characterization of seven glutathione S-transferase genes from citrus red mite, *Panonychus citri* (McGregor). Int. J. Mol. Sci..

[46] Tan X., Hu X., Zhong X., Chen Q., Xia Q., Zhao P. (2014). Antenna-specific glutathione S-transferase in male silkmoth *Bombyx mori*. Int. J. Mol. Sci..

[47] Reddy B.P., Prasad G.B.K.S., Raghavendra K. (2011). In silico analysis of glutathione S-transferase supergene family revealed hitherto unreported insect specific δ- and ε-GSTs and mammalian specific μ-GSTs in *Ixodes scapularis* (Acari: Ixodidae). Comput. Biol. Chem..

[48] Lumjuan N., McCarroll L., Prapanthadara L.A., Hemingway J., Ranson H. (2005). Elevated activity of an epsilon class glutathione transferase confers DDT resistance in the dengue vector, *Aedes aegypti*. Insect Biochem. Mol..

[49] Krishna N.S.R., Getchell T.V., Getchell M.L. (1994). Differential expression of α-class, μ-class, and π-class of glutathione S-transferases in chemosensory mucosae of rats during development. Cell Tissue Res..

[50] Feng Y.C., Liao C.Y., Xia W.K., Jiang X.Z., Shang F., Yuan G.R., Wang J.J. (2015). Regulation of three isoforms of *SOD* gene by environmental stresses in citrus red mite, *Panonychus citri*. Exp. Appl. Acarol..

[51] Ogoma S.B., Moore S.J., Maia M.F.A. (2012). Systematic review of mosquito coils and passive emanators: Defining recommendations for spatial repellency testing methodologies. Parasite Vector.

[52] Rinker D.C., Pitts R.J., Zhou X., Suh E., Rokas A., Zwiebel L.J. (2013). Blood meal-induced changes to antennal transcriptome profiles reveal shifts in odor sensitivities in *Anopheles gambiae*. Proc. Natl. Acad. Sci. USA.

[53] Falda M., Toppo S., Pescarolo A., Lavezzo E., Di Camillo B., Facchinetti A., Cilia E., Velasco R., Fontana P. (2012). Argot^2^: A large scale function prediction tool relying on semantic similarity of weighted Gene Ontology terms. BMC Bioinf..

[54] Katoh K., Standley D.M. (2013). MAFFT Multple sequence alignment software version 7: Improvements in performance and usability. Mol. Biol. Evol..

[55] Waterhouse A.M., Procter J.B., Martin D.M., Clamp M., Barton G.J. (2009). Jalview version 2: A multiple sequence alignment editor and analysis workbench. Bioinformatics.

[56] Kriventseva E.V., Tegenfeldt F., Petty T.J., Waterhouse R.M., Simão F.A., Pozdnyakov I.A., Ioannidis P., Zdobnov E.M. (2015). OrthoDB v8: Update of the hierarchical catalog of orthologs and the underlying free software. Nucleic Acids Res..

[57] Donohue K.V., Khalil S., Rosee E., Roe R.M. (2010). Neuropeptide signaling sequences identified by pyrosequencing of the American dog tick synganglion transcriptome during blood feeding and reproduction. Insect Biochem. Mol. Biol..

[58] Untergasser A., Nijveen H., Rao X., Bisseling T., Geurts R., Leunissen J.A. (2007). Primer3Plus, an enhanced web interface to Primer3. Nucleic Acids Res..

[59] Carr A.L., Roe R.M. (2016). Acarine attractants: Chemosensation, bioassay, chemistry and control. Pestic. Biochem. Phyiol..

[60] Bingman V.P., Graving J.M., Hebets E.A., Wiegmann D.D. (2016). Importance of the antenniform legs, but not vision, for homing by the neotropical whip spider, *Paraphrynus laevifrons*. J. Exp. Biol..

