# Supplementary material for: Tick Haller’s Organ, a New Paradigm for Arthropod Olfaction: How Ticks Differ from Insects
Source: Int J Mol Sci. 2017 Jul 18;18(7):1563. doi: 10.3390/ijms18071563 (PMC5536051; doi:10.3390/ijms18071563)
Supplement: Supplementary file 1 [file ijms-18-01563-s001.pdf]

Tick Haller's Organ, a New Paradigm for Arthropod Olfaction: How Ticks Differ from Insects

Supplementary Materials

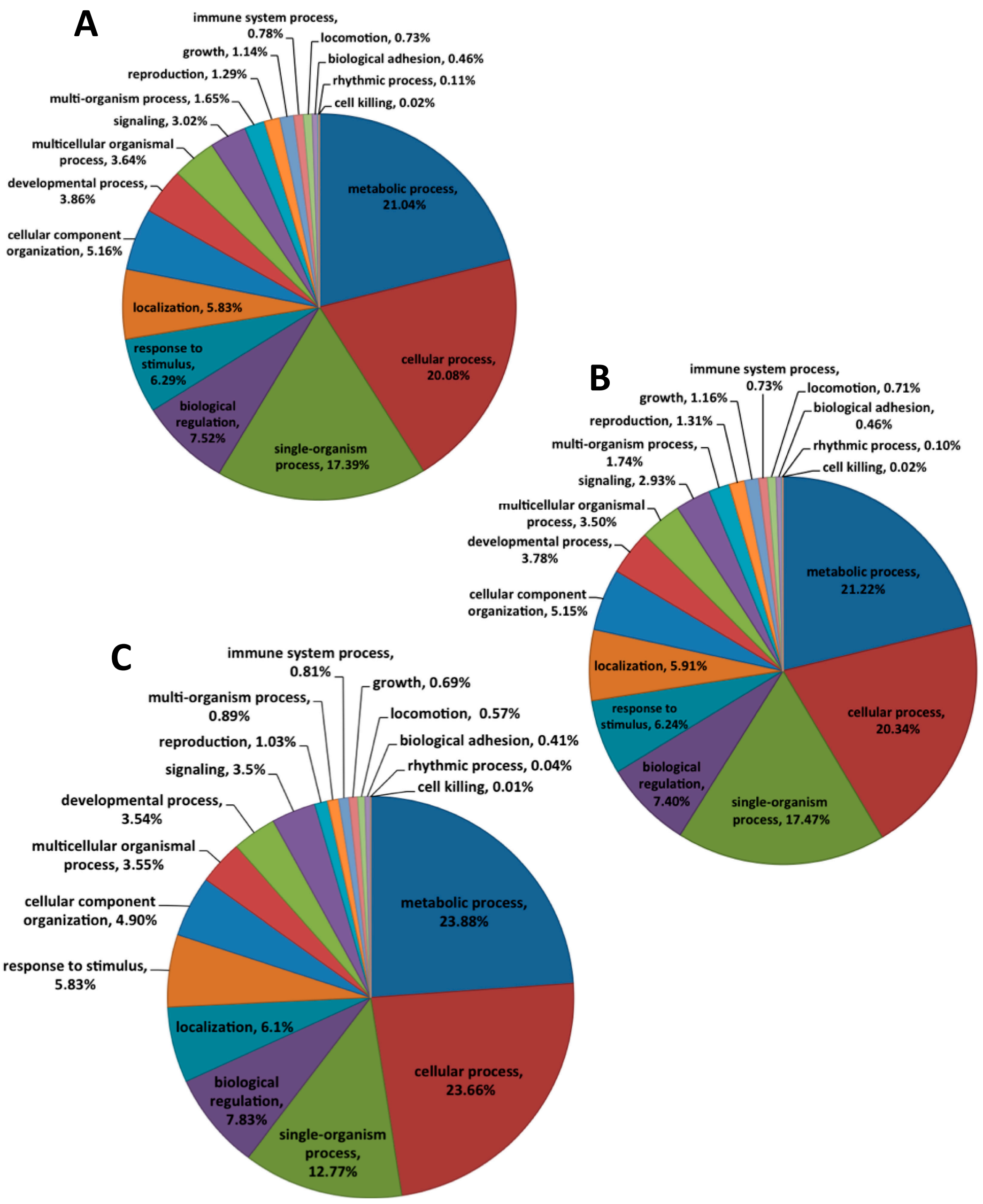

Figure S1. Distribution of transcripts annotated at the gene ontology level 2 and their putative involvement in biological functions for **A**, the unfed virgin adult male

Dermacentor variabilis illumina 1st leg transcriptome; **B**, the unfed virgin adult male *D. variabilis* illumina 4th leg transcriptome; **C**, the unfed virgin adult male and female *D. variabilis* 454 1st leg transcriptome.

```
Contig_73        MCDDEVAALVVDNGSGMCKAGFAGDDAPRAVFPSIVGRPRHQGVMVGMGQKDSYVGDEAQ
Contig_146       MCDDEVAALVVDNGSGMCKAGFAGDDAPRAVFPSIVGRPRHQGVMVGMGQKDSYVGDEAQ
RmAAP79880.1     MCDDEVAALVVDNGSGMCKAGFAGDDAPRAVFPSIVGRPRHQGVMVGMGQKDSYVGDEAQ
                 ************************************************************

Contig_73        SKRGILTLKYPIEHGIVTNWDDMEKIWHHTFYNELRVAPEEHPVLLTEAPLNPKANREKM
Contig_146       SKRGILTLKYPIEHGIVTNWDDMEKIWHHTFYNELRVAPEEHPVLLTEAPLNPKANREKM
RmAAP79880.1     SKRGILTLKYPIEHGIVTNWDDMEKIWHHTFYNELRVAPEEHPVLLTEAPLNPKANREKM
                 ************************************************************

Contig_73        TQIMFETFNTPAMYVAIQAVLSLYASGRTTGIVLDSGDGVSHTVPIYEGYALPHAILRLD
Contig_146       TQIMFETFNTPAMYVAIQAVLSLYASGRTTGIVLDSGDGVSHTVPIYEGYALPHAILRLD
RmAAP79880.1     TQIMFETFNTPAMYVAIQAVLSLYASGRTTGIVLDSGDGVSHTVPIYEGYALPHAILRLD
                 ************************************************************

Contig_73        LAGRDLTDYLMKILTERGYSFTTTAEREIVRDIKEKLCYVALDFEQEMATAASSSSLEKS
Contig_146       LAGRDLTDYLMKILTERGYSFTTTAEREIVRDIKEKLCYVALDFEQEMATAASSSSLEKS
RmAAP79880.1     LAGRDLTDYLMKILTERGYSFTTTAEREIVRDIKEKLCYVALDFEQEMATAASSSSLEKS
                 ************************************************************

Contig_73        YELPDGQVITIGNERFRCPEALFQPSFLGMESCGIHETTYNSIMKCDVDIRKDLYANTVL
Contig_146       YELPDGQVITIGNERFRCPEALFQPSFLGMESCGIHETTYNSIMKCDVDIRKDLYANTVL
RmAAP79880.1     YELPDGQVITIGNERFRCPEALFQPSFLGMESCGIHETTYNSIMKCDVDIRKDLYANTVL
                 ************************************************************

Contig_73        SGGTTMYPGIADRMQKEITALAPSTMKIKIIAPPERKYSVWIGGSILASLSTFQQMWISK
Contig_146       SGGTTMYPGIADRMQKEITALAPSTMKIKIIAPPERKYSVWIGGSILASLSTFQQMWISK
RmAAP79880.1     SGGTTMYPGIADRMQKEITALAPSTMKIKIIAPPERKYSVWIGGSILASLSTFQQMWISK
                 ************************************************************

Contig_73        QEYDESGPSIVHRKCF
Contig_146       QEYDESGPSIVHRKCF
RmAAP79880.1     QEYDESGPSIVHRKCF
                 ****************
```

Figure S2. Multiple sequence alignment (Clustal Ω) of the deduced amino acid sequence for actin (contig 73, 1st legs; contig 146, 4th legs) identified in the top 50 most abundant transcripts of the illumina 1st and 4th leg transcriptomes of unfed, virgin adult male *Dermacentor variabilis* versus the top GenBank BLAST hit (lowest expect value; Table 1, Table 2). Asterisks (*) denote conserved residues. The acronym consists of the first letter of genus and species (*Rhipicephalus microplus*, Rm) followed by the GenBank accession number for the protein BLAST hit (AAP79880.1).

```
AaJAT93369.1      LQSLLDHSNKSQANAEKQVKQLEVQLADAQFKLDETNRTLNDLDGSKKKMGVENSELQRQ
Contig_86         ----------------------EVQLADAQFKLDETNRTLNDMDGSKKKMGVENSELQRQ
Contig_36         --------------AEKQVKQLEVQLADAQFKLDETNRTLNDMDGSKKKMGVENSELQRQ
                                        *********************:****************

AaJAT93369.1      LEEAESQVAQLNKIKASLATQLEEAKRQADEEARERAAILGKYRNLEHDLDNLRESVEEE
Contig_86         LEEAESQVAQLNKIKASLATQLEEAKRQADEEARERAAILGKYRNLEHDLDNLRESIEEE
Contig_36         LEEAESQVAQLNKIKASLATQLEEAKRQADEEARERAAILGKYRNLEHDLDNLRESIEEE
                  ********************************************************:***

AaJAT93369.1      QEAKADFQRQLSKANAEAQLWRSKYESEGLARLEELEEAKRKLHGKLQEAEEAMEQLNAK
Contig_86         QEAKADFQRQLSKANAEAQLWRSKYESEGLARLEELEEAKRKLHAKLQEAEEAMEQLNAK
Contig_36         QEAKADFQRQLSKANAEAQLWRSKYESEGLARLEELEEAKRKLHAKLQEAEEAMEQLNAK
                  ********************************************.***************

AaJAT93369.1      CSGLEKTKAHLQGELEDMSIEVDKANALAASLEKRQKSFDKVIAEWKAKVDDLAAELDAS
Contig_86         CSGLEKTKAHLQGELEDMSIEVDKANALAASLEKRQKSFDKVIAEWKAKVDDLAAELDAS
Contig_36         CSGLEKTKAHLQGELEDMSIEVDKANALAASLEKRQKSFDKVIAEWKAKVDDLAAELDAS
                  ************************************************************

AaJAT93369.1      QKECRNYSTEVFKLRAAYEESQEHYEAVKRENKNLQDEIKDLMDQLGEGGRSVHELEKSR
Contig_86         QKECRNYSTEVFKLRAAYEESQEHYESVKRENKNLQDEIKDLMDQLGEGGRSVHELEKSR
Contig_36         QKECRNYSTEVFKLRAAYEESQEHYESVKRENKNLQDEIKDLMDQLGEGGRSVHELEKSR
                  **************************:*********************************

AaJAT93369.1      KRLEMEKEELQAALEEAEAALEQEENKVLRAQLELSQVRQEIDRRIQEKEEEFENTRKNH
Contig_86         KRLEMEKEELQAALEEAEAALEQEENKVLRAQLELSQVRQEIDRRIQEKEEEFENTRKNH
Contig_36         KRLEMEKEELQAALEEAEAALEQEENKVLRAQLELSQVRQEIDRRIQEKEEEFENTRKNH
                  ************************************************************

AaJAT93369.1      QRALDSMQASLEAEAKGKAEALRLKKKLESDINELEIALDHANKANAEAQKNLKKYQQNV
Contig_86         QRALDSMQASLEAEAKGKAEALRLKKKLESDINELEIALDHANKANAEAQKNLKKYQQNV
Contig_36         QRALDSMQASLEAEAKGKAEALRLKKKLESDINELEIALDHANKANAEAQKNLKKYQQNV
                  ************************************************************

AaJAT93369.1      KDLQTALEEEQRARDEAREQYASAERRCNALHGELEESRQLLEQSDRARRAGEAELSEMH
Contig_86         KDLQTALEEEQRARDEAREQYASAERRCNALHGELEESRQLLEQSDRARRAGEAELSEMH
Contig_36         KDLQTALEEEQRARDEAREQYASAERRCNALHGELEESRQLLEQSDRARRAGEAELSEMH
                  ************************************************************

AaJAT93369.1      ETVNELSAQTASLSVAKRKLEGEMQALQADLDEVLNEAKQSEEKAKKAMVDAARLADELR
Contig_86         ETVNELSAQTASLSVAKRKLEGEMQALQADLDEVLNEAKQSEEKAKKAMVDAARLADELR
Contig_36         ETVNELSAQTASLSVAKRKLEGEMQALQADLDEVLNEAXXXXXXXXKAMVDAARLADELR
                  **************************************        **************

AaJAT93369.1      AEQDHALQQEKLRKALEQQMKELQVRLDEAEAAALKGGKKIIQKLEQKVRELENELENEQ
Contig_86         AEQDHALQQEKLRKALEQQMKELQVRLDEAEAAALKGGKKIIQ-----------------
Contig_36         AEQDHALQQEKLRKALEQQMKELQVRLDEAEAAALKGGKKIIQKLEQKVRELENELENEQ
                  *******************************************

AaJAT93369.1      RRHGDAAKNFRKGERRIKELQFQAEEDRKNHERMQDLVDKLQQKIKTYKRQIEEAEEIAA
Contig_86         -----------KSERRIKELQFQAEEDRKNHERMQDLVDKLQQKIKTYKRQIEEAEEIAA
Contig_36         RRHGDAAKNFRKSERRIKELQFQAEEDRKNHERMQDLVDKLQQKIKTYKRQIEEAEEIAA
                             *.***********************************************

AaJAT93369.1      LNLAKFRKVQQELEDAEERADMAENTLAKLRAKNRSSASAGRAMSPGLASAPLRT-
Contig_86         LNLAKFRKVQQELEDAEERADMAENTLAKLRAKNRSSASAGRAMSPGLSSAPPLRT
Contig_36         LNLAKFRKVQQELEDAEERADMAENTLAKLRAKNRSSASAGRAMSPGLSSAPPLRT
                  *************************************************:***
```

Figure S3. Multiple sequence alignment (Clustal Ω) of the deduced amino acid sequence for myosin (contig 36, 1st legs; contig 86, 4th legs) identified in the top 50 most abundant transcripts of the illumina 1st and 4th leg transcriptomes of unfed virgin adult male *Dermacentor variabilis* versus the top GenBank BLAST hit (lowest expect value; Table 1, Table 2). Asterisks (*) denote conserved residues, colons (:) indicate conservation between groups of strongly similar properties scoring >0.5 in the Gonnet PAM 250 matrix, and periods (.) indicate conservation between groups of weakly similar properties scoring ≤0.5 on the Gonnet PAM 250 matrix. The acronym consists of

the first letter of genus and species (*Amblyomma aureolatum*, Aa) followed by the GenBank accession number for the protein BLAST hit (JAT93369.1).

```
RaJAP88178.1        SPKMTEAEMLMEEKLRKKKEEEEEMWAEYLEQRKKQRAKEEEELRKLKERQMKRKAQRAE
Contig_22           ---------------------------------------------------------RAE
Contig_42           STKMTEAEMLMEEKLRKKKEEEEEMWAEYLEQRKKQRAKEEEELRKLKERQAKRKAQRAE
                                                                             ***

RaJAP88178.1        QEAKLMEFKKKQEEQRIREMEEKKAREAEAKRKRLEEAEKKRQAMLAAKEKREQEPVKPN
Contig_22           QEAKLMEFKKKQEEQRIKEMEEKKAREAEAKRKRLEEAERKRQAMLAAKEKREQEPVKPN
Contig_42           QEAKLMEFKKKQEEQRIKEMEEKKAREAEAKRKRLEEAERKRQAMLAAKEKREQEPVKPN
                    *****************:*********************:********************

RaJAP88178.1        FVITKREGEDMA-SALGSSGFDKFTNIMYARGEMGKTKEQMEEDKKAILSFRIKPLEIEG
Contig_22           FVITKREGEDMAGSALGSSGFDKFANIMYARGEMGKTKEQMEEDKKAILSFRIKPLEIEG
Contig_42           FVITKREGEDMAGSALGSSGFDKFANIMYARGEMGKTKEQMEEDKKAILSFRIKPLEIEG
                    ************ ***********:***********************************

RaJAP88178.1        LTVEQLREKAKQLWERVVSLESEKYDLEERQKRQDYDLKELAERQRQINRSRALKKGLDP
Contig_22           LTVEQLREKAKELWEKVVNLESEKYDLEERQKRQDYDLKELAERQRQINRSRALKKGLDP
Contig_42           LTVEQLREKAKELWEKVVNLESEKYDLEERQKRQDYDLKELAERQRQINRSRALKKGLDP
                    ***********:***:**.*****************************************

RaJAP88178.1        EALQGKHPPKIQVASKYERRTDRRTFTDKRELFSGGLEAMIDAESEKQWEEKMLSFKESA
Contig_22           EALQGKHPPKIQVASKYERRTDRRTFVDKRELFSGGLEAMLDAESEKQWEEKMVNFKESA
Contig_42           EALQGKHPPKIQVASKYERRTDRRTFVDKRELFSGGLEAMLDAESEKQWEEKMVNFKESA
                    **************************.*************:************:.*****

RaJAP88178.1        RSGLPKWDPENPKNKQVSRTRRMEEEDEDEELEPVFEPPKPVETPKPAPPAPKPAPPPQQ
Contig_22           RGGLPKWDPENPKNKQVMRSRREVDEEEDDELEPVFEPPKPIE-----------------
Contig_42           RGGLPKWDPENPKNKQVMRSRREVDEEEDDELEPVFEPPKPIETPKPAPPAPKPAPVQQ-
                    *.*************** *:**  :*:**:***********:*

RaJAP88178.1        EEEEEEEEEEEEEEEDEEEEEE
Contig_22           ----------------------
Contig_42           ----------------------
```

Figure S4. Multiple sequence alignment (Clustal Ω) of the deduced amino acid sequence for troponin T (contig 22, 1st legs; contig 42, 4th legs) identified in the top 50 most abundant transcripts of the illumina 1st and 4th leg transcriptomes of unfed virgin adult male *Dermacentor variabilis* versus the top GenBank BLAST hit (lowest expect value; Table 1, Table 2). Asterisks (*) denote conserved residues, colons (:) indicate conservation between groups of strongly similar properties scoring >0.5 in the Gonnet PAM 250 matrix, and periods (.) indicate conservation between groups of weakly similar properties scoring ≤0.5 on the Gonnet PAM 250 matrix. The black bar shows the troponin domain. The acronym consists of the first letter of genus and species (*Rhipicephalus appendiculatus*, Ra) followed by the GenBank accession number for the protein BLAST hit (JAP88178.1).

```
Contig_17          MEAIKKKMQAMKLEKDNAVDRAETAEGQSREAALRAEKAEEEVRSLQKKIQQIENELDQV
Contig_239         MEAIKKKMQAMKLEKDNAVDRAETAEGQSREAALRAEKAEEEVRSLQKKIQQIENELDQV
RmAMB19056.1       MEAIKKKMQAMKLEKDNAVDRAETAEQQSREAALRAEKAEEEVRSLQKKIQQIENELDQV
                   ************************** *********************************

Contig_17          QEQLSQANSKLEEKDKALQAAEAEVAAHNRRIQLLEEDLERSEERLKIATQKLEEASQAA
Contig_239         QEQLSQANSKLEEKDKALQAAEAEVAAHNRRIQLLEEDLERSEERLKIATQKLEEASQAA
RmAMB19056.1       QEQLSQANSKLEEKDKALQAAEAEVAAHNRRIQLLEEDLERSEERLKIATQKLEEASQAA
                   ************************************************************

Contig_17          DESERMRKMLEHRSITDEERMDGLEGQLKEARTMAEDADRKYDEVARKLAMVEADLERAE
Contig_239         DESERMRKMLEHRSITDEERMDGLEGQLKEARTMAEDADRKYDEVARKLAMVEADLERAE
RmAMB19056.1       DESERMRKMLEHRSITDEERMDGLEGQLKEARTMAEDADRKYDEVARKLAMVEADLERAE
                   ************************************************************

Contig_17          ERAETGETKIVELEEELRVVGNNLKSLEVSEEKALQKEETYEMQIRQMTNRLQEAEARAE
Contig_239         ERAETGETKIVELEEELRVVGNNLKSLEVSEEKALQKEETYEMQIRQMTNRLQEAEARAE
RmAMB19056.1       ERAETGETKIVELEEELRVVGNNLKSLEVSEEKALQKEETYEMQIRQMTNRLQEAEARAE
                   ************************************************************

Contig_17          FAERSVQKLQKEVDRLEDELVQEKEKYKAISDELDQTFSELTGY
Contig_239         FAERSVQKLQKEVDRLEDELVQEKEKYKAISDELDQTFSELTGY
RmAMB19056.1       FAERSVQKLQKEVDRLEDELVQEKEKYKAISDELDQTFSELTGY
                   ********************************************
```

Figure S5. Multiple sequence alignment (Clustal Ω) of the deduced amino acid sequence for tropomyosin (contig 17, 1st legs; contig 239, 4th legs) identified in the top 50 most abundant transcripts of the illumina 1st and 4th leg transcriptomes of unfed virgin adult male *Dermacentor variabilis* versus the top GenBank BLAST hit (lowest expect value; Table 1, Table 2). Asterisks (*) denote conserved residues. The acronym consists of the first letter of genus and species (*Rhipicephalus microplus*, Rm) followed by the GenBank accession number for the protein BLAST hit (AMB19056.1).

```
Contig_6805       --------QYQDLVRAMISFFDYFAQLEKKLQETHEYEALLAEAKQRAEHLATLVDKTEP
Contig_4832       --------QYQDLVRAMISFFDYFAQLEKKLQETHEYEALLAEAKQRAEHLATLVDKTEP
IsEEC05627.1      QSFQENISKYQDLVKAMINFFNYFSQLEKQLQDRQDDEALLDEAKRQADHLAALVDRTEP
                          :*****:***.**:**:****:**: ::  **** ***::*:***:***:***

Contig_6805       SEAAVLDRDKIHSLVDNQGALLVASLRKSHQDSQMSKFNDELHLINQQVRELTDSLKRDS
Contig_4832       SEAAVLDRDKIHSLVDNQGALLVASLRKSHQDSQMSKFNDELHLINQQVRELTDSLKRDS
IsEEC05627.1      SEAAVLDRDKIHFLVDNRRQMLAASLRKSQQDSQMSKFNEELQQINHQVRELTDSLKRDS
                  ************ ****:  :*.******:*********:**: **:*************

Contig_6805       SKCHVVEFQQRIQSLERRIEVLSTSSRLQTDSARMELEELTSRWRNFHKEAMAAPPQPQP
Contig_4832       SKCHVVEFQQRIQSLERRIEVLSTSSRLQTDSARMELEELTSRWRNFHKEAMAAPPQPQP
IsEEC05627.1      SKCHM--------TLEKRIEILSTSSRTQTDSARMELEQLTSRWRAFHKEAMAPPPQPLD
                  ****:        :**:***:****** **********:****** ******* ****

Contig_6805       IDKKMQEVKEVCGPSFVVPLDDVEVQEGAKCSLVCVLAGHPPPTVQWYKDGVSIRGNSDY
Contig_4832       IDKKMQEVKEVCGPSFVVPLDDVEVQEGAKCSLVCVLAGHPPPTVQWYKDGVSIRGNSDY
IsEEC05627.1      ----RKEANQICGPSFVVPLDDVEVQDGSKCSLVCVLAGQPAPTVQWYKDGVSVRGNSDY
                       :*.:::***************:*:**********:* ***********:******

Contig_6805       VTTSQQLEDGTTEQRLTIEETLADDSAKFCCKAINAAGLAETSCRLSVREQPRDSALEEV
Contig_4832       VTTSQQLEDGTTEQRLTIEETLADDSAKFCCKAINAAGLAETSCRLSVREQPRDSALEEV
IsEEC05627.1      VTASQPLEDGSSEQKLTIEETLADDSAKFTCKAINAAGMAETSCRLSVREQPRQQELEEV
                  **:** ****::**:************** ********:**************:. ****

Contig_6805       FIEALEEHTTQTVGGVLTEPTFVQPLPAIAQLRPGEELRLECTVQGRPEPRVSWSKDLLP
Contig_4832       FIEALEEHTTQTVGGVLTEPTFVQPLPAIAQLRPGEELRLECTVQGRPEPRVSWSKDLLP
IsEEC05627.1      FIEALEQSV---ETGTFQEPTFVKPLPATARFRPGDQLRLECRVEGQPEPRVSWSKDLLP
                  ******: .     *.: *****:**** *::***::***** *:*:*************

Contig_6805       VRDAAKPTQEQGRARLLLAGATEGDSGTYTAVAKNKAGETACSCQVKVAEDAPPQEPPRV
Contig_4832       VRDAAKPTQEQGRARLLLAGATEGDSGTYTAVAKNKAGETACSCQVKVAEDAPPQEPPRV
IsEEC05627.1      VRDAAKSSLERGTAVLVLNDAVQSDSGTYTAVAQNKAGQTACSSQVKVAEDVPPAEPPRV
                  ****** : *:* * *:* .*.:.*********:****:****.*******.** *****

Contig_6805       LKALEDLEVKPGPDPVTLECVIVGRPEPEVIWYHNTQPIKESERVRLLFRGDKCSLLFNG
Contig_4832       LKALEDLEVKPGPDPVTLECVIVGRPEPEVIWYHNTQPIKESERVRLLFRGDKCSLLFNG
IsEEC05627.1      LKGLVDLEVKPGPEPVTLECIIVGEPEPEVIWYHDDQPIKESERVRLLFQGDKCSLVLNG
                  **.* ********:******:***.*********: *************:******::**

Contig_6805       IGAQNAGTYRCSAVNPMGSCYTECKLRVPLSAPVFLEPLRDVTTDEGCRVVLTAKLWAPE
Contig_4832       IGAQNAGTYRCSAVNPMGSCYTECKLRVPLSAPVFLEPLRDVTTDEGCRVVLTAKLWAPE
IsEEC05627.1      VSAQNAGTYRCSAVNTMGSCFTQCRLRVPLAAPVFLEPLRDVNVAEGARVDLSAKLWAPE
                  :.************* ****:*:*:*****:***********.. **.** *:*******

Contig_6805       PPFVHWFKDGRELLPSPDFQVSHDPDGTVKLLIPKVAGNNSGHYEVEASNPGGRTRTGCK
Contig_4832       PPFVHWFKDGRELLPSPDFQVSHDPDGTVKLLIPKVAGNNSGHYEVEASNPGGRTRTGCK
IsEEC05627.1      PPFVRWFKDGNEVVPGSDYQIKHDTDGAVRLTIPEAFNEDGGHYELEASNAGGRTRTGCR
                  ****:*****.*::*. *:*:.** **:*:* **:. .::.****:**** ********:

Contig_6805       MHV-----------------------------------------
Contig_4832       MHV-----------------------------------------
IsEEC05627.1      VHVERVEERFRISREKWRHYATHHERTTIVLTSRKMRSMVLAAGV
                  :**
```

Figure S6. Multiple sequence alignment (Clustal Ω) of the deduced amino acid sequence for titin (contig 4832, 1st legs; contig 6805, 4th legs) identified in the top 50 most abundant transcripts of the illumina 1st and 4th leg transcriptomes of unfed virgin adult male *Dermacentor variabilis* versus the top GenBank BLAST hit (lowest expect value; Table 1, Table 2). Asterisks (*) denote conserved residues, colons (:) indicate conservation between groups of strongly similar properties scoring >0.5 in the Gonnet PAM 250 matrix, and periods (.) indicate conservation between groups of weakly similar properties scoring ≤0.5 on the Gonnet PAM 250 matrix. The black bar shows the immunoglobulin intermediate-set domain. The acronym consists of the first letter of

genus and species (*Ixodes scapularis,* Is) followed by the GenBank accession number for the protein BLAST hit (EEC05627.1).

```
contig_343          ----------------------------------------------GCCAAGGTC----G
AaJZ183505.1        CTGGAGTTGTTGGGTCTATACCACACTGTTCCCTTTGTCCTTTCTGGTCGGAGTCGAGGA
                                                                  * *...***    .

contig_343          TCTGATATGAAGACTGCCGTGGTGTTTGTGCTGGCCGCCTGCCTTGTGGCTGTGGCGATG
AaJZ183505.1        TTTGTGATGAAGGCTGCTGTGGTGTTTCTGCTGACAGCCTGCCTCCTGGCCAGGGCC---
                    * **: ******.**** ********* *****.*.********  **** . ***

contig_343          AGTGCTGGAGGCCGGGCTTCGCGCCGT---GAAGAATTCAACAGATGGAGGGACTGTATG
AaJZ183505.1        AGCGCTGGCAGCCGGGCTGAGAGCCGCCGGGCCCAGTTTGACCAATGGAGGCAGTGCATG
                    ** *****..******** .*.****    *.. *.** .**..******* * ** ***

contig_343          GTTGGAAAGCTACCGACTGATAAAATACAGGAGTACCAAACCTGCCGCAACCAGTCCCGT
AaJZ183505.1        GTGGATAAGCTTCCCACTGACAAAGCCCCAGTGTTTCAGGGCTGCCACAACACAGCGGCT
                    ** *.:*****:** ***** ***. .*..*:**: **.. *****.****... *   *

contig_343          GGTACTGAGATGCGCAGGTTCAGACAGGGCCTGGAGTGCGTTCTGAGAAGCTACAACCTC
AaJZ183505.1        GGAACAGAGATGCGCAAGTTCAGACAGGGCCTGGAATGTGTCCTGGACAGCTACCAACTC
                    **:**:**********.******************.** ** ***...******.*.***

contig_343          GTTGAAAAGAACGATGTCAACTTGAGCCGCATGAGGGAGCTGGCTCAGAATGTCACAAAG
AaJZ183505.1        GTGGAAAGGAACAATGTGAACTTGGCCCGGATGACTGAGGTGGCTCCCACCATCGCCAAG
                    ** ****.****.**** ******. *** ****  *** ******. *. .**.*.***

contig_343          CCGGAACTGAGGGCTGCTTTTGAAGAGTGCCCCCAGGACGAACACAACAGCAG-AGTCGC
AaJZ183505.1        GAGGAGCTGAAGAAAGCCTTTGAAGACTGCCCCAGGGACGAAGGCAACAAAAGGGGTGGC
                     .***.****.*..:** ******** ******..******* .*****..** .** **

contig_343          AAAGGCGGTGAAGTGCGTCATCGACCGCCTCGAAACGTCCTGCCCTGTACCTGCTGGTGA
AaJZ183505.1        AAAGGCTGTCAAGTGCGTCATCGACCACCTTGAAACCACGTGCCCTGTACCAAGCGGC--
                    ****** ** ****************.*** ***** :* ***********:.  **

contig_343          GGACAGGGAGTAAAGACTGACATCGTTGCGACGTCAGACAGCCCACTGGATAACCACTAT
AaJZ183505.1        GGACAGAGCT-AAATGCTGCCATCACCGCCA-----------------------------
                    ******.*.  *** .***.****.  ** *
```

Figure S7. Multiple sequence alignment (Clustal Ω) of the nucleic acid sequence for a putative *Amblyomma americanum* odorant-binding protein coding expressed sequence tag (EST; JZ183505.1) versus a homologous transcript (contig 343) identified in the 4th leg transcriptome of unfed virgin adult male *Dermacentor variabilis*. Asterisks (*) denote conserved residues, colons (:) indicate conservation between groups of strongly similar properties scoring >0.5 in the Gonnet PAM 250 matrix, and periods (.) indicate conservation between groups of weakly similar properties scoring ≤0.5 on the Gonnet PAM 250 matrix. The acronym consists of the first letter of genus and species (*Amblyomma americanum,* Aa) followed by the GenBank accession number for the protein EST (JZ183505.1).

```
contig_114           ---------------------CACGACGGAA-GTCTGTATC--GCGCGTAACAAGCGGCT
AaJZ172282.1         GCAGCTTCTCTCCACTATCAGCCCAGCGGAGCGACTG-AGCGTGCGCAAACATGGTGGCT
                                          *.*..****. *:*** * *  ****.:*..:.* ****

contig_114           TA-CGGTCGGCCCTCAGGCTGTGGGTGCCCGTACGCACAACGCTCTTAAGGCGTTGTGAA
AaJZ172282.1         TTCAAGGCGGCCCTCCTCCTCTGTGTGCTCGTCCTTCCGGCGGCCTACTGCC----AGTC
                     *: ..* ********.  ** ** **** ***.*  .*..**  **:.:* *    :*:.

contig_114           GGCGTCTTAGCG-ACTCTCTCGGTCACCTCACCGGCCAGGAATGATGCGCTCGCAACAGG
AaJZ172282.1         GGCGGCCGAGCCCCCGCGCCCGGACATC-----AACTGGGGAAA--GTGCCCCCAGCTGC
                     **** *  ***  .* * * ***:** *     ..* .**.*:.  * ** * **.*:*

contig_114           CTC-TACAAATGGGGAAA------TGAATGGCTTCTTTATTGACGACTGTTGAGCTTACA
AaJZ172282.1         AGCCATCTAAGGAGGAAAGACAACAGAAGGCCCTAGTCATTGACACCTGCCTCGAG---A
                     . * ::*:** *.*****      :*** * * *. * ******..***   .*.    *

contig_114           GGGTTTCTGTATTTTTTCTTTCCGGCATAGTGTACTGTCGCTCTTCCTTTGTCCGTATGC
AaJZ172282.1         AGGT---------GCCCCTTCCCGATGTCGAGCACGCTAACGAGACCGTGATCCAGCAGC
                     .***             *** ***. .*.*:* **  *..* . :** * .***. .:**

contig_114           -CCGAGTGGCATTTCCCCTTTGGTTGTGACGCCGAGTAGGAAGAAGGAAGCCGGCGTGCG
AaJZ172282.1         ACCGCGAGGATGTCACCACTTGCGCTCTCCACAGCGAGGGAT------------------
                      ***.*:**.: * .**. ***      .*.*.*.*:.***:

contig_114           AAGTGAGACAAACAGAAAACCCAGAGGACCGTCGCGGTTCAGCAGGAAGTCGGCAGAAAC
AaJZ172282.1         ----GGTTCAACAAGAACGGCCA-------GTATCGCTTCGACCGT-----GCCAGGACC
                         *. :***..****.. ***       **. ** ***..*.*      * ***.*.*

contig_114           GAGGAAATGACTGAAGAGCCCAGGCTGACGATTCACGGGGCTGATTTAGATCACAACGTT
AaJZ172282.1         GAGATCCTGAACA---------------AGAAGCTCGCCGCCGACGTCGAGCCCAA-GGT
                     ***.:..***. .               .**: *:**  ** **  *.** *.*** * *

contig_114           GCTTTGAGAACTCGGAGTCAGTCGGGGGCTCGGTGCGAGACTTAACCATGGGCCGGCGCG
AaJZ172282.1         GC-------TCGCCAAGCACGACGAGTGCAAGAAGGA------GGCCGAGGAGAAGTTCG
                     **       :* * .** ..*:**.* **:.*.:* .      ..**.:**. ..*  **

contig_114           CCAGCCTGTGGCCCGCCCTGGATCTG-------------------GATGCCACAGATCTG
AaJZ172282.1         CCCACCAGTTCGTCGCCCAGGTGCAGCTGTACCAGGCCTGCATGGACTACCACATCTCCC
                     **..**:**    *****:**: *:*                   ..*.***** .**

contig_114           AGAGATGTGGTAATC-----CATGCAAGCC----TGGTACAGCTGCACCTGAGCCACAAA
AaJZ172282.1         AGATCTGTGGCATCCAGATCCAGGGTGCCCAGGGCGGAGCTGCCGCGCCGGCC-C----A
                     *** .***** *: *     ** * :. **     **:.*:** **.** *.  *    *

contig_114           CTGGTGGGCGAACTTCTCCTCGGCCTCCTTCTTGCACTCGTCGTGCTTAGCGAGCACCTT
AaJZ172282.1         CGGCTAAGCCAACGCCGCCG-AGCCAACTTCTCGCTGACTCCGAGTTCTCAAAGCAACGT
                     * * *..** ***  * **  .***:.***** **: :*  **:* * : ..****.* *

contig_114           AGAGCTTCTTGTTCAAGATC-TCAGTCCTGGCACGGTCGAAGCG-GTACTGGCC------
AaJZ172282.1         TGTGATCCAAATCATGAATCGTCAGCCTGGGCTCTGCAGCTTCCAGTCATTTCCTCGTTT
                     :*:*.* *::.* .:..*** **** *  ***:* * .*.: *  **..*  **

contig_114           -----G--TTCTTGTTGAACCAT-CCTTCGCTGTGGAGAGCGCAGGTGGTGACGTCCTCA
AaJZ172282.1         CTTCCGACTTCCTGCTGAACAGACCCCCCGCTGAAA------------------------
                          *  *** ** *****..: **  *****:..
```

Figure S8. Multiple sequence alignment (Clustal Ω) of the nucleic acid sequence for a putative *Amblyomma americanum* odorant-binding protein coding expressed sequence tag (EST; JZ172282.1) versus a homologous transcript (contig 114) identified in the 4th leg transcriptome of unfed virgin adult male *Dermacentor variabilis*. Asterisks (*) denote conserved residues, colons (:) indicate conservation between groups of strongly similar properties scoring >0.5 in the Gonnet PAM 250 matrix, and periods (.) indicate conservation between groups of weakly similar properties scoring ≤0.5 on the Gonnet

PAM 250 matrix. The acronym consists of the first letter of genus and species (*Amblyomma americanum,* Aa) followed by the GenBank accession number for the protein EST (JZ172282.1).

```
contig_466         ARRWHGHGTSLKSSFQARARGVSRRSFNTETIMLPSVCLALLVVSFVDRNGVYANPKTMK
contig_39297       -----------------RARGVSRRSFNTETIMLPSVCLALLVVSFVDRNGVYANPKTMK
AaJZ171538.1       --------------------------PAR*TTMLPLSLIALIASALTA*NGVQASPKTLM
                                                 * ***   :**:. ::.  *** *.***:

contig_466         DDFNPLKFKGKWWVDSFNQAIYGDADRCAHFTIHKDHDDVYKIKAEYIDTDNELVEMSVE
contig_39297       DDFNPLKFKGKWWVDSFNQAIYGDADRCAHFTIHKDHDDVYKIKAEYIDTDNELVEMSVE
AaJZ171538.1       DNFIPSKFEGKWWVDSFSQVIYGDADRCAHFTIKKNHDNVYKIQAEYVDSDNELVEMTVD
                   *:* * **:********.*.*************:*:**:****:***:*:*******:*:

contig_466         VKEDDRHPSRFILKINADVIMETAIIDTDYDNWAVVWAKSGTAAAYHVVTRKPNAEDQFL
contig_39297       VKEDDRHPSRFILKINADVIMETAIIDTDYDNWAVVWAKSGTAAAYHVVTRKPNAEDQFL
AaJZ171538.1       VNEDDRHPSRFILKIDADNIFETAIIDTDYDNWAIVWAKSGTAAAYHVVTRKPNAEDQFL
                   *:*************:** *:*************:*************************

contig_466         PAIQAALDKEGLKKDDFKKVPNMDCSKKDTI*LTESK*ICX--
contig_39297       PAIQAALDKEGLKKDDFKKVPNMDCSKKDTI*LTESK*ICNKX
AaJZ171538.1       PAIQKALDKEGLKKDAFRKVPNMDCTKKDKHLGVKEINECYX-
                   **** ********** *:*******:***.   .:.   *
```

Figure S9. Multiple sequence alignment (Clustal Ω) of the deduced amino acid sequence for a lipocalin (contig 39297, 1st legs; contig 466, 4th legs) identified in the illumina 1st and 4th leg transcriptomes of unfed virgin adult male *Dermacentor variabilis* versus the putative *Amblyomma americanum* chemosensory lipocalin coding expressed sequence tag (EST; JZ171538.1). Asterisks (*) denote conserved residues, colons (:) indicate conservation between groups of strongly similar properties scoring >0.5 in the Gonnet PAM 250 matrix, and periods (.) indicate conservation between groups of weakly similar properties scoring ≤0.5 on the Gonnet PAM 250 matrix. The acronym consists of the first letter of genus and species (*Amblyomma americanum,* Aa) followed by the GenBank accession number for the protein EST (JZ171538.1).

```
contig_84287     -----------------------------------------TVALHKVMYLDKKGGCII
AtJAC30054.1     TLRARQYKIVNDLISMKVIFKVSKTGVHKDYNAVRYQHGLDQPWRERKLMYISPEKTCAI
                                          :*:**:. :  * *

contig_84287     LVQDIGDGKKGCQLLRTEAYIETDVPEECHRVYKQNCKGTVLKMYERKCKRYNQVLIPRT
AtJAC30054.1     LVEELGKGRKGCQLVQPESAIDDGIPEECHKIYKASCGKTSVQVYEHACRSLPDASPRHR
                 **:..* .*.*****:: *: *: .:*****..**  .*  * :..**: *:   :.   :

contig_84287     L*THFICIAYHTARX
AtJAC30054.1     EL-------------
```

Figure S10. Multiple sequence alignment (Clustal Ω) of the deduced amino acid sequence for the putative lipocalin (contig 84287) identified exclusively in the Haller's organ spf transcriptome of unfed virgin adult male *Dermacentor variabilis* versus the top BLAST GenBank hit (lowest expect value; Table 3). Asterisks (*) denote conserved residues, colons (:) indicate conservation between groups of strongly similar properties scoring >0.5 in the Gonnet PAM 250 matrix, and periods (.) indicate conservation between groups of weakly similar properties scoring ≤0.5 on the Gonnet PAM 250 matrix. The acronym consists of the first letter of genus and species (*Amblyomma triste*, At) followed by the GenBank accession number for the protein BLAST hit (JAC30054.1).

```
contig_4943      VSRLLLLAQADPNARTNCLNRAPLLVLSAYEGFT--------------------------
contig_8166      VSRLLLLAQADPNARTNCLNRAPLLVLSAYEGFT--------------------------
IsEEC13968.1     ------------------------------------------------------------
HaAHV83756.1     VPLHSAVHGGDIKAVELCLKSGAKISTQQYDLSTPVHLACAQGALEIVKLMFTMQPTEKL
CpAOR16348.1     TVLQAAIEAEWVPGVCVALEAGADVTLRANDGETPIHSAAAIGNIEVLSEILSLAKQSKF
CpAAOR16350.1    TVLQAAIEAEWVPGVCVALEAGADVTLRANDGETPIHSAAAIGNIEVLSEILSLAKQSKF

contig_4943      -----------------------DMVTSLVEFGADVNVASDVGRTALSMACERGHFEIAK
contig_8166      -----------------------DMVTSLVEFGADVNVASDVGRTALSMACERGHFEIAK
IsEEC13968.1     --------------------------------GASMTATDKEGRSVILLAAARGAWKTVT
HaAHV83756.1     ACLTSCDVQKMTPVHCAAMFDHPDIVNYLISEGSDINPLDKERRSPLLLAASRAGWRTVH
CpAOR16348.1     -----------------------------------IDCQNDEGETPLFKAITNGHVACVR
CpAAOR16350.1    -----------------------------------IDCQNDEGETPLFKAITNGHVACVR
                                                   :   ..  .: :  *  ..    .

contig_4943      YLLSCGARIDHVDSEGLSALA-YAASGHLDLVTHLVQCKWPDDFGIALGLHRAAQQALI
contig_8166      YLLSCGARIDHVDSEGLSALA-YAASGHLDLVTHLVQCKWPDDFGIALGLHRAAQQALI
IsEEC13968.1     TLLTLGADLTVKDNLRRNLLHHIVLSGGCLD------------DF---------------
HaAHV83756.1     TLIRLGADIELKDINSRNVLHLVVMNGGRLE------------DF---------------
CpAOR16348.1     ALLKDGAAIDKTLPGDINVLH-VAADHGHLE-----------------------------
CpAAOR16350.1    ALLKDGAAIDKTLPGDINVLH-VAADHGHLE-----------------------------
                  *:  ** :         . *    . . * *:

contig_4943      AAAKSGHTEVCEFLLD---MMEVQVNKQDDFSGHTALTAASLAGHRDVCALLMRRGASAL
contig_8166      AAAKSGHTEVCEFLLD---MMEVQVNKQDDFSGHTALTAASLAGHRDVCALLMRRGASAL
IsEEC13968.1     ------YNEINERLQD-----FALLLNERDMQGCTAMHYASRNGQLKTIEGLL-------
HaAHV83756.1     ------AATCKLTLQNRCDKSLAQLLNEKDSAGCSPLHYASREGHIRSLENLIRLGACIN
CpAOR16348.1     --------ILKCLLEYDRSYQMLNVLTAADRRGMGPIHFAVSGNHYECVKFLLEKNADIR
CpAAOR16350.1    --------ILKCLLEYDRSYQMLNVLTAADRRGMGPIHFAVSGNHYECVKFLLEKNADIR
                           *           : .  *  *  .:  *   .:      *:

contig_4943      VRSN----AGEPPLCCAVREGHWAVTELLLCHARA---LEQADALGRSPLMLAAAEGHLG
contig_8166      VRSN----AGEPPLCCAVREGHWAVTELLLCHARA---LEQADALGRSPLMLAAAEGHLG
IsEEC13968.1     ------------------QYGRYNTVRHLLESKKGHLIINEMDGEGKTPLHIASQNGHTR
HaAHV83756.1     LKNS----NNESPLHFAARYGRYHTACQLLDSDKGTFIINESDGEGLTPLHIASREGHTR
CpAOR16348.1     LRTTCSPHKSSTPLHIAAAKNYSEVAKVILVMDKTT--IHEVNSMGWFPLHTAAHHSSRE
CpAAOR16350.1    LRTTCSPHKSSTPLHIAAAKNYSEVAKVILVMDKTT--IHEVNSMGWFPLHTAAHHSSRE
                                .    ..  :*    :    :.: :. *   **  *: ..

contig_4943      VLELLLAKGANLLWQDKDGLSALSWSCSRGQLQAAQCLLT-HGADINHSDKRGRTPLDMA
contig_8166      VLELLLAKGANLLWQDKDGLSALSWSCSRGQLQAAQCLLT-HGADINHSDKRGRTPLDMA
IsEEC13968.1     VVHLLLVKGA-LLHRDHRGRTPLHYAVTNGFTHTMEQLLAVHSHLLDQSDRDG-------
HaAHV83756.1     VVHLLLNRGA-LLHRDHNGRNPLHLAAMSGYTQTIELLHSVHSHLLDQSDKDGNTPLHLA
CpAOR16348.1     VIALLLKDGA-NL----------------AGYT------------------DGPKKF---
CpAAOR16350.1    VIALLLKDGA-NL----------------AGYT------------------DGPKKF---
                 *: ***  **  *                *                    *

contig_4943      ATQGNAEVIKLLLNNGAEIEHVDLNGTRALDRAIG-----------TGNLDAINCFLQAG
contig_8166      ATQGNAEVIKLLLNNGAEIEHVDLNGTRALDRAIG-----------TGNLDAINCFLQAG
IsEEC13968.1     ------------------------------------------------------------
HaAHV83756.1     TMENKPNSIALLLSMGCRLSYNNLDMS-AIDYAIYYKFPEAALAMVTHEHRAKEVMALRS
CpAOR16348.1     ----RRTAIDMLL--------NNLSK------------PTEYLEEVLDTYITSNTQNLQ-
CpAAOR16350.1    ----RRTAIDMLL--------NNLSK------------PTEYLEEVLDTYITSNTQNLQ-

contig_4943      AKIGSQTWEMAAGKPDVVHLLLNKLHQDGALLYRKGHMKDAVHRY-QYALKKFPSSVVE-
contig_8166      AKIGSQTWEMAAGKPDVVHLLLNKLHQDGALLYRKGHMKDAVHRY-QYALKKFPSSVVE-
IsEEC13968.1     ------------------------------------------------------------
HaAHV83756.1     DRHPCVTLALIAYMPRVFEAVQDK------CITKANCKKDSKSFYIKYSFKFYQRSKLEV
CpAOR16348.1     DNDSVVTVDFAVLIPNVCEMEQMK------VIE---------------------------
CpAAOR16350.1    DNDSVVTVDFAVLIPNVCEMEQMK------VIE---------------------------
```

Figure S11. Multiple sequence alignment (MAFFT, E-INS) of the deduced amino acid sequence for the putative TRPA transcripts (contig 4943 and 8166) identified in the illumina 1st and 4th leg transcriptomes of unfed virgin adult male *Dermacentor variabilis* versus the *Ixodes scapularis* TRPA homolog and putative insect olfactory TRPA splice variants. Asterisks (*) denote conserved residues, colons (:) indicate conservation between groups of strongly similar properties scoring >0.5 in the Gonnet PAM 250 matrix, and periods (.) indicate conservation between groups of weakly similar properties scoring ≤0.5 on the Gonnet PAM 250 matrix. The black bar shows the 3 copies of Ankyrin repeats. The acronym consists of the first letter of genus and species (*Ixodes scapularis*, Is; *Helicoverpa armigera*, Ha; *Cydia pomonella*, Cp) followed by the GenBank accession number (EEC13968.1; AOR16348.1l AOR16350.1; AHV83756.1).

```
contig_72702      WKHSSRSAKMGQQTSSSLPRAKVKTLKMTAVVFGAFLVTNVPYMVQEAILAFGNPGILDA
IsEEC06829.1      ---------------------MVKTLKMTVVVFGAFLVTNVPYMVQEMILAFGNPGILDH
                                        *******.***************** ***********

contig_72702      NLVALFGVISASNSAINPYIFL--------------------------------------
IsEEC06829.1      NVVALFGVISASNSAINPYIYFFFQRSKRQCGKFCSALLKDAKTWFSCRRLRNLCNSQSA
                  *:******************::

contig_72702      ------------------------------------
IsEEC06829.1      SSPVTMTSLHLNGDYAMTSWNTGTQVEIRSPNKVDV
```

Figure S12. Multiple sequence alignment (Clustal Ω) of the deduced amino acid sequence for the putative G-protein coupled receptor (contig 72702) identified exclusively in the Haller's organ spf transcriptome of unfed virgin adult male *Dermacentor variabilis* versus the top BLAST GenBank hit (lowest expect value; Table 3). Asterisks (*) denote conserved residues, colons (:) indicate conservation between groups of strongly similar properties scoring >0.5 in the Gonnet PAM 250 matrix, and periods (.) indicate conservation between groups of weakly similar properties scoring ≤0.5 on the Gonnet PAM 250 matrix. The acronym consists of the first letter of genus and species (*Ixodes scapularis*, Is) followed by the GenBank accession number for the protein BLAST hit (EEC06829.1).

```
contig_83622      ------------------------------------------------------------
AsJAT99189.1      ARYGLEPSFQSCTIDWRHNDASYKSFTVVYFVLGFLVPACIVVVCYRTSAAHIRVPKPTV


contig_83622      ----DMNDDFWANQDSVTMMVVLIVATFFAAWTPYAVLCLWAVFGKASAVPHLVAVVPPL
AsJAT99189.1      VRRTDMNDDFWANQESVTTMVVLIVAAFFAAWTPYAVLCLWAVFGNVASVPHLVAVLPPL
                      **********:*** *******:******************:.::*******:***


contig_83622      FCKTASAINPFIYFFSNPRIRTDIYALLTCRCKTLGRRSCSIQEDYC
AsJAT99189.1      FCKTASAINPFIYFFSNPRIRTDIYALLTCRCQAMGRRSCSIEEDYC
                  ********************************:::*******:****
```

Figure S13. Multiple sequence alignment (Clustal Ω) of the deduced amino acid sequence for the putative G-protein coupled receptor (contig 83622) identified exclusively in the Haller's organ spf transcriptome of unfed virgin adult male *Dermacentor variabilis* versus the top BLAST GenBank hit (lowest expect value; Table 3). Asterisks (*) denote conserved residues, colons (:) indicate conservation between groups of strongly similar properties scoring >0.5 in the Gonnet PAM 250 matrix, and periods (.) indicate conservation between groups of weakly similar properties scoring ≤0.5 on the Gonnet PAM 250 matrix. The acronym consists of the first letter of genus and species (*Amblyomma sculptum*, As) followed by the GenBank accession number for the protein BLAST hit (JAT99189.1).

```
contig_13937      MGCAMSAEERAALARSKQIEKNLKEDGIQAAKDIKLLLLGAGESGKSTIVKQMKIIHDSG
RpJAA58325.1      ----MSAEERAALARSKQIEKNLKEDGIQAAKDIKLLLLGAGESGKSTIVKQMKIIHDSG
                      ********************************************************

contig_13937      FTQEDFKQYKPVVYSNTIQSMVAILRAMPNLGISFGNNEREADAKMVFDVVARMEDTEPF
RpJAA58325.1      FTQEDFKQYKPVVYSNTIQSMVAILRAMPNLGISFGNNEREADAKMVFDVVARMEDTEPF
                  ************************************************************

contig_13937      SEELLSAMKRLWTDSGVQECFGRSNEYQLNDSAKYFLDDLDRLGKKDYMPTEQDILRTRV
RpJAA58325.1      SEELLSAMKRLWTDSGVQECFGRSNEYQLNDSAKYFLDDLDRLGKKEYMPTEQDILRTRV
                  **********************************************:*************

contig_13937      KTTGIVEVHFSFKNLNFKLFDVGGQRSERKKWIHCFEDVTAIIFCVAMSEYDQVLHEDET
RpJAA58325.1      KTTGIVEVHFSFKNLNFKLFDVGGQRSERKKWIHCFEDVTAIIFCVAMSEYDQVLHEDET
                  ************************************************************

contig_13937      TNRMQESLKLFDSICNNKWFTDTSIILFLNKKDLFEEKIKKSPLTICFPEYTGAQEYGEA
RpJAA58325.1      TNRMQESLKLFDSICNNKWFTDTSIILFLNKKDLFEEKIKKSPLTICFPEYTGAQEYGEA
                  ************************************************************

contig_13937      AAYIQAQFEAKNKSTTKEIYCHMTCATDTTNIQFVFDAVTDVIIANNLRGCGLY
RpJAA58325.1      AAYIQAQFEAKNKSTTKEIYCHMTCATDTTNIQFVFDAVTDVIIANNLRGCGLY
                  ******************************************************
```

Figure S14. Multiple sequence alignment (Clustal Ω) of the deduced amino acid sequence for the putative G-protein α_o subunit (contig 13937) identified exclusively in the Haller's organ spf transcriptome of unfed virgin adult male *Dermacentor variabilis* versus the top BLAST GenBank hit (lowest expect value; Table 3). Asterisks (*) denote conserved residues and colons (:) indicate conservation between groups of strongly similar properties scoring >0.5 in the Gonnet PAM 250 matrix. The black bar shows the Gα subunit domain. The acronym consists of the first letter of genus and species (*Rhipicephalus pulchellus*, Rp) followed by the GenBank accession number for the protein BLAST hit (JAA58325.1).

```
Contig_24477      ---------------------------------------------------ISSDGAYAL
IrJAB79904.1      QIATNPKFPDTVLSSSRDKTLILWRLTRDDASYGVATRRLRGHGHFVTDVVLSSDGQYAL
                                                                     :**** ***

Contig_24477      SASWDKSLRLWELETGNTTRTFVGHTNDVLSVSFSADNRQIVSGSRDRTIKLWNTLGDC-
IrJAB79904.1      SCSWDKTLRLWDLAVGSSTRRFEGHEKDVLSVAFSADNRQIVSGSRDKTIKLWNTLAECK
                  *.****:****:* .*.:** * ** :*****:**************:********.:*
```

Figure S15. Multiple sequence alignment (Clustal Ω) of the deduced amino acid sequence for the putative G-protein β subunit (contig 24477) identified exclusively in the Haller's organ spf transcriptome of unfed virgin adult male *Dermacentor variabilis* versus the top BLAST GenBank hit (lowest expect value; Table 3). Asterisks (*) denote conserved residues, colons (:) indicate conservation between groups of strongly similar properties scoring >0.5 in the Gonnet PAM 250 matrix, and periods (.) indicate conservation between groups of weakly similar properties scoring ≤0.5 on the Gonnet PAM 250 matrix. The black bar shows the WD Gβ repeat domain. The acronym consists of the first letter of genus and species (*Ixodes ricinus*, Ir) followed by the GenBank accession number for the protein BLAST hit (JAB79904.1).

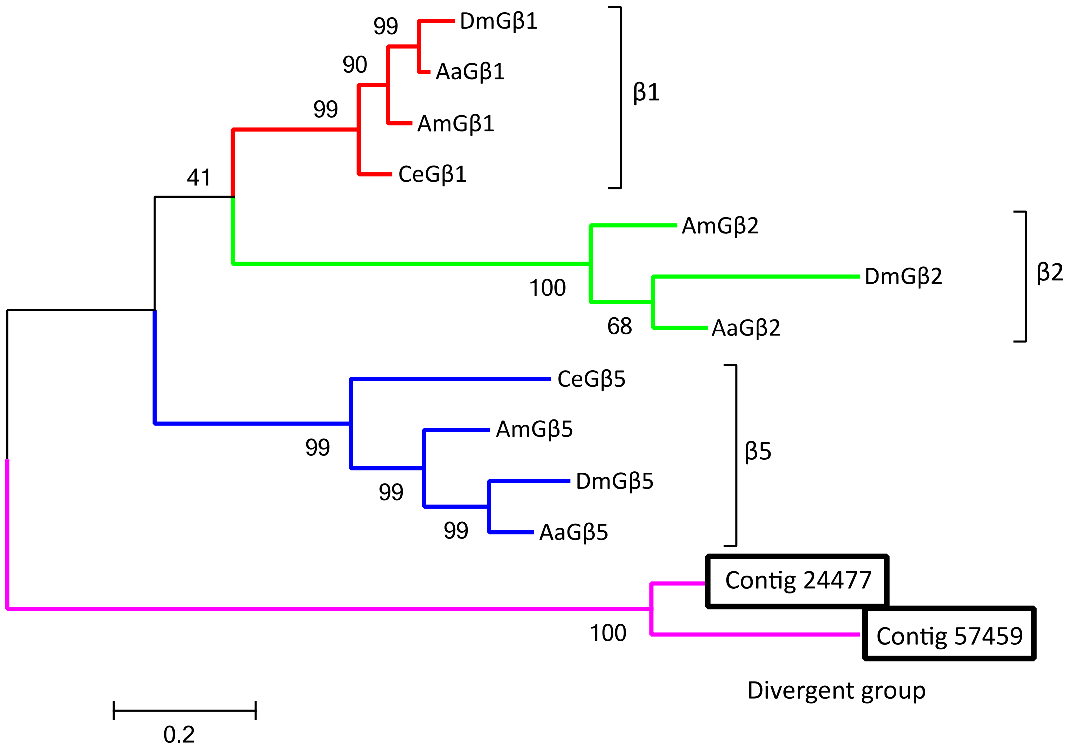

Figure S16. Phylogenetic relationship of a transcript putatively encoding G-protein β subunits (Gβ) identified in the Haller's organ spf transcriptome (contig 24477) and a transcript found common in both the illumina 1st and 4th leg transcriptomes (contig 57459, 1st legs) of unfed, virgin adult male *Dermacentor variabilis* with Gβ subunits of known clade annotation from *Caenorhabditis elegans* and insects. The phylogenetic tree shows four clades, each represented by a branch color as follows: red = β1 clade; green = β2 clade; blue = β5 clade; purple = novel divergent clade. Acronyms are as follows: first letter of the genus and species (*Aedes aegypti*, Aa; *Apis mellifera*, Am; *Drosophila melanogaster*, Dm; *Caenorhabditis elegans*, Ce) followed by the protein name (Gβ) and the number of the clade number. Putative Gβ subunit transcripts are boxed. The tree was constructed using Maximum likelihood phylogenetic analysis and bootstrapping set to 500 iterations. Branch values listed are bootstrap percentages (percent confidence), scale set to 20%. A comprehensive list of acronyms and associated GenBank accession numbers are listed in Appendix A.

```
contig_3088         SWLQSARPHR*TSASGVLDTTPVAQAVSALFSR*FSLRSGSARLKFSGPREAYPSYDSGA
RpJAA63082.1        --------------------------------PTRQTRHGP--GSQSSRGKAYPSLHEGA
                                                       . * *    . *.  :**** ..**

contig_3088         TTAADAMDKESIKKQIENLRYQAQMERWPLSKSIQALREYIEENERTDPLIHAPDKKSNP
RpJAA63082.1        TTAYTTMDKESIKKQIENLRYQAQMERWPLSKSIQALREYIEENERTDPLIHAPDKKSNP
                    ***  :******************************************************

contig_3088         WAEKGKCLIM*TEDISALLTPPAGLLVLLLVLFWPYYYFA*NQQAGRDSPDETP*LLQQS
RpJAA63082.1        WAEKGKCLIM--------------------------------------------------
                    **********
```

Figure S17. Multiple sequence alignment (Clustal Ω) of the deduced amino acid sequence for the putative G-protein γ subunit (contig 3088) identified in the illumina 4th leg transcriptome of unfed virgin adult male *Dermacentor variabilis* versus the top BLAST GenBank hit (lowest expect value; Table 3). Asterisks (*) denote conserved residues, colons (:) indicate conservation between groups of strongly similar properties scoring >0.5 in the Gonnet PAM 250 matrix, and periods (.) indicate conservation between groups of weakly similar properties scoring ≤0.5 on the Gonnet PAM 250 matrix. The black bar shows the WD Gβ repeat domain. The acronym consists of the first letter of genus and species (*Rhipicephalus pulchellus*, Rp) followed by the GenBank accession number for the protein BLAST hit (JAA63082.1).

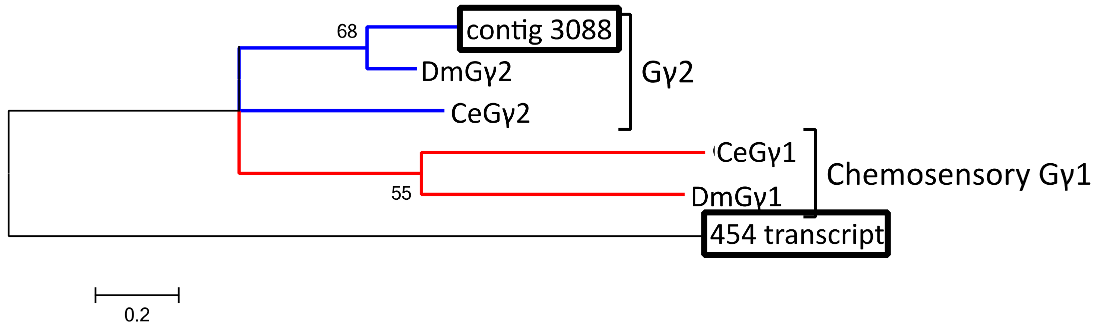

Figure S18. Phylogenetic relationship of transcripts putatively encoding G-protein γ subunits (Gγ) identified in the illumina 4th leg transcriptome (contig 3088) and the 454 1st leg transcriptome (454 transcript) of unfed, virgin adult male *Dermacentor variabilis* with Gγ subunits of known clade annotation from *Caenorhabditis elegans* and *Drosophila melanogaster*. The phylogenetic tree shows two clades, each represented by a branch color as follows: red = γ1, chemosensory clade; blue = γ2 clade. Acronyms are as follows: first letter of the genus and species (*Drosophila melanogaster*, Dm; *Caenorhabditis elegans*, Ce) followed by the protein name (Gγ) and the number of the clade number. Putative Gγ subunit transcripts are boxed. The tree was constructed using Maximum likelihood phylogenetic analysis and bootstrapping set to 500 iterations. Branch values listed are bootstrap percentages (percent confidence), scale set to 20%. A comprehensive list of acronyms and associated GenBank accession numbers are listed in Appendix A.

```
contig_77721      -----------------------------------------AFFAGGWGNDPEITHCALL
IsEEC01411.1      TNSFKDDKLELAYQGYSHRQRQTALVIVNLIDVFLKVAMLVAFFVAGELDEPR-LTCALL
                                                           ***..*  ::*.   ****

contig_77721      RYLPWVLVNVLLSLLTCWRFFANNYLHWGAALIWIALNAQGNGNFGVTSGELRFEPGGDV
IsEEC01411.1      RNLPWIAVNLLLCLLTYWKFFASNYLHWGALLIWIALNAEGNGTFGVTWGYLQFEPGGDV
                  * ***: **:**.*** *:***.******* ********:***.**** * *:*******

contig_77721      SX----------------------------------------------------------
IsEEC01411.1      AGDGSWHVMFTVFVTYAMLPLPLKWCIVCGVLASLGHLLVCCLYRAHLDRPEFERMILTN
                  :
```

Figure S19. Multiple sequence alignment (Clustal Ω) of the deduced amino acid sequence for the putative adenylate/guanylate cyclase (contig 77721) identified exclusively in the Haller’s organ spf transcriptome of unfed virgin adult male *Dermacentor variabilis* versus the top BLAST GenBank hit (lowest expect value; Table 3). Asterisks (*) denote conserved residues, colons (:) indicate conservation between groups of strongly similar properties scoring >0.5 in the Gonnet PAM 250 matrix, and periods (.) indicate conservation between groups of weakly similar properties scoring ≤0.5 on the Gonnet PAM 250 matrix. The acronym consists of the first letter of genus and species (*Ixodes scapularis*, Is) followed by the GenBank accession number for the protein BLAST hit (EEC01411.1).

```
contig_37845      ---------------------------------------------------------RIE
IsEEC13610.1      ASAADLAVSVSTFCRAFPFHFMCDRQLRLTQLGRGLARIFGGRGSAVPSLFVFLEPELLE
                                                                            :*

contig_37845      MRFDHVVANINLPFLLQVRDDAIKHERYKGMEIKGQMVHCPESRALLFLGSPVVDGGLSA
IsEEC13610.1      MRFDHVVAATNLPFLLQVRDDAIKHQRYKGMEVKGQMVHCPESETLLFLGSPVVDGGLSA
                  ********  ***************:******:**********.:***************

contig_37845      MLRRGLYISDVPVHDATRDILLVEEQSRAQDGLKRRMDKIRASIQEANLAVEEERQKNVD
IsEEC13610.1      MLRRGLYISDVPVHDATRDILLVEEQARAQDGLKRRMDKIRSSIQEANLAVEEERQKNVD
                  **************************:**************:******************

contig_37845      LLHLIFPPRVARKLWLGESMEAQQHDQATLLFSDIVGFTAICSTATPMMVINMLX-----
IsEEC13610.1      LLHLIFPPSVARKLWLGESVEAQQHDQVTLLFSDIVGFTAICSTATPMMIETVGDAYCVA
                  ******** **********:*******.*********************: .:
```

Figure S20. Multiple sequence alignment (Clustal Ω) of the deduced amino acid sequence for the putative adenylate/guanylate cyclase (contig 37845) identified exclusively in the Haller's organ spf transcriptome of unfed virgin adult male *Dermacentor variabilis* versus the top BLAST GenBank hit (lowest expect value; Table 3). Asterisks (*) denote conserved residues, colons (:) indicate conservation between groups of strongly similar properties scoring >0.5 in the Gonnet PAM 250 matrix, and periods (.) indicate conservation between groups of weakly similar properties scoring ≤0.5 on the Gonnet PAM 250 matrix. The solid black bar shows the heme-NO binding domain, and the dashed black bar shows the guanylate cyclase catalytic domain. The acronym consists of the first letter of genus and species (*Ixodes scapularis*, Is) followed by the GenBank accession number for the protein BLAST hit (EEC13610.1).

```
contig_82720      ---------------------------------------QKCSDEERSVGCLPDKLKAEI
IsEEC03664.1      MKTHQISKLDAVKTYMRMRHVPDHLQNKVIRWFDYLWLTQKSSDEERSVGCLPDKLKAEI
                                                         **.******************

contig_82720      AIHVHLDTLKRVEIFQNTEAGFLCELVLRLRPVLFSPGDYICRKGEVGKEMYIVNRGRLQ
IsEEC03664.1      AIHVHLDTLKRVEIFQNTEAGFLCELVLRLRPVLFSPGDYICRKGEVGKEMYIVNRGRLQ
                  ************************************************************

contig_82720      VVTDNGK-----------------------------------------------------
IsEEC03664.1      VVTDNGKTVLATLRAGSYFGEISILNMGTAGNRRTASVRSVGYSDLFCLYKQDMWDVLKD
                  *******
```

Figure S21. Multiple sequence alignment (Clustal Ω) of the deduced amino acid sequence for the putative cyclic nucleotide-gated ion channel (contig 82720) identified exclusively in the Haller's organ spf transcriptome of unfed virgin adult male *Dermacentor variabilis* versus the top BLAST GenBank hit (lowest expect value; Table 3). Asterisks (*) denote conserved residues, colons (:) indicate conservation between groups of strongly similar properties scoring >0.5 in the Gonnet PAM 250 matrix, and periods (.) indicate conservation between groups of weakly similar properties scoring ≤0.5 on the Gonnet PAM 250 matrix. The black bar shows the cyclic nucleotide binding domain. The acronym consists of the first letter of genus and species (*Ixodes scapularis*, Is) followed by the GenBank accession number for the protein BLAST hit (EEC03664.1).

```
CeCGNG          MSAARTEFQNKMDGIKQYMELRKVSKQLEIRVIKWFDYLWTNKQSLSDQQVLKVLPDKLQ
Contig_82720    ------------------------------------------QKCSDEERSVGCLPDKLK
DmCNG           MNVARVEFQNRMDGVKQYMAFRRVGHELEARVIRWFAYTWSQSGALDEERVLAALPDKLK
                                                          . . .::: :  *****:

                                                    ________________________
CeCGNG          AEIAMQVHFETLRKVRIFQDCEAGLLAELVLKLQLQVFSPGDFICKKGDIGREMYIVKRG
Contig_82720    AEIAIHVHLDTLKRVEIFQNTEAGFLCELVLRLRPVLFSPGDYICRKGEVGKEMYIVNRG
DmCNG           AEIAIQVHMDTLKQVRIFHDTEPGLLEALVLKLKLQVFSPGDYICRKGDVGKEMYIVKRG
                ****::**::**::*.**:: * *:*  ***:*:  :*****:**:**::*:*****:**

                __________
CeCGNG          RLQVVDDDGKKVFVTLQEGSVFGELSILNIAGSKNGNRRTANVRSVGYTDLFVLSKTDLW
Contig_82720    RLQVVTDNGK--------------------------------------------------
DmCNG           KLSVVGDDGITVLATLGAGSVFGEVSVLEIAGNRTGNRRTANVRSLGYSDLFCLAKRDLW
                :*.** *:*
```

Figure S22. Multiple sequence alignment (Clustal Ω) of the deduced amino acid sequence for the putative cyclic nucleotide-gated ion channel (CNG) α- subunit (contig 82720) identified exclusively in the Haller's organ spf transcriptome of unfed virgin adult male *Dermacentor variabilis* versus *Caenorhabditis elegans* and *Drosophila melanogaster* chemosensory CNG α- subunits. Asterisks (*) denote conserved residues, colons (:) indicate conservation between groups of strongly similar properties scoring >0.5 in the Gonnet PAM 250 matrix, and periods (.) indicate conservation between groups of weakly similar properties scoring ≤0.5 on the Gonnet PAM 250 matrix. The black bar shows the cyclic nucleotide binding domain. The acronym consists of the first letter of genus and species (*Caenorhabditis elegans*, Ce; *Drosophila melanogaster*, Dm) followed by the protein name (CNG).

```
contig_01853      TIGTEAAAAEESAKRQGGTRVFKKSSPNGKITMYLGKRDFVDHITSVDPIDGVVLIDPDY
IsEEC07926.1      --------------IVIPFRVFKKSSPNSKITMYLGKRDFVDHITSVDPIDGVVLIDPDY
                                     *********.*******************************

contig_01853      VKERKVFGHVLAAFRYGREDLDVLGLTFRKDLYLASEQIYPPLAE--TAGRPLTRLQERL
IsEEC07926.1      VKDRKVFGHVLAAFRYGREDLDVLGLTFRKDLYLASEQIYPRLTTGENSNRPLTRLQVSS
                  **:************************************** *:   .: *******

contig_01853      LRKLGPNAYPFYFEL-PPHCPASVTLQPAPGDTGKPCGVDYELKGYVADS---PEDKPHK
IsEEC07926.1      ESSFFFFFLSFFYYLFVRNLGWGGTTFPATRLPDGFCNP-QRVNGHCSTAEVTQPRFCSY
                    .:      *:: *   .   . *  **       *    .::*: : :

contig_01853      RNSVRLAIRKIMYAPSRQGEQPSVEVSKEFVMSPNKLHLEASLDKELYHHGEDIAVNVHI
IsEEC07926.1      RNSVRLAIRKIMYAPSRQGEQPSVEVSKEFVMSPNKLHLEASLDKELYHHGEDIAVNVHI
                  ************************************************************

contig_01853      ANNSNRTVKKVKVSVRQFADICLFSTAQYKCTVAEIDSEEGCPVSPGFTLSKVYYLRPLL
IsEEC07926.1      ANNSNRTVKKVKVSVRQFADICLFSTAQYKCTVAEIDSEEGCPVSPGFTLSKVHYLRPLL
                  *****************************************************:******

contig_01853      ANNKDKRGLALDGQLKHEDTNLASSTIITDPAQKENLGIIVQYKVKVKLCLGPLGGDLVA
IsEEC07926.1      ANNKDKRGLALDGQLKHEDTNLASSTM---------------------------------
                  **************************:

contig_01853      ELPFILMHPKPEESSPIRVVSEPKAPGPVPLDTNLIELDTDAAASLDDDDIIFEDFARLR
IsEEC07926.1      ------------------------------------------------------------
```

Figure S23. Multiple sequence alignment (Clustal Ω) of the deduced amino acid sequence for the putative β-arrestin (contig 1853) identified exclusively in the Haller's organ spf transcriptome of unfed virgin adult male *Dermacentor variabilis* versus the top BLAST GenBank hit (lowest expect value; Table 3). Asterisks (*) denote conserved residues, colons (:) indicate conservation between groups of strongly similar properties scoring >0.5 in the Gonnet PAM 250 matrix, and periods (.) indicate conservation between groups of weakly similar properties scoring ≤0.5 on the Gonnet PAM 250 matrix. The solid black bar shows the arrestin amino terminal domain, and the dashed black bar shows the arrestin carboxyl terminal domain. The acronym consists of the first letter of genus and species (*Ixodes scapularis*, Is) followed by the GenBank accession number for the protein BLAST hit (EEC07926.1).

```
contig_69591      ----SAQTLTWTLFALAINPGVQRRVHDELDRVFGKRAACNITKSHVSKLTYLDRVLKET
IsEEC03681.1      GHDTVTQSLTWTLFVLGIYPDVQSKVHEELDLIFAHDMTRGITRADIADLSYLDRVIKVS
                       :*:******.*.* * ** :**:*** :*.:  :  **::.::.*:*****:* :

contig_69591      MRIFTIVPWVGRSLTEPLKIGNCTIPEGCTCYVFTYGIHRDPTHYTDPEVFDPDRFLPEK
IsEEC03681.1      RAFFYHITVSNAS--RVRIFRNYQIPKGTTCFVFTYGLHRDPDHYRDPETFDPDRFLPEN
                    :*  :     *  .   : *  **:* **:*****:**** ** ***.*********:

contig_69591      CSRNHPFAFVPFSAGPRNCIGQKFAMLELKVLLAKVLTNFSVSSCNHRDDLLFDADILLR
IsEEC03681.1      CSGRHPFAFVPFSAGPRNCVGQKFALMELKVTLAKLLRRYQVKSCHQRDDLLLMADMLLR
                  ** .***************:*****::**** ***:* .:.*.**.:*****: **:***

contig_69591      TKRPIRIRLQPRHDTX
IsEEC03681.1      TRNPIKFQLTERLAPQ
                  *:.**:::*  *
```

Figure S24. Multiple sequence alignment (Clustal Ω) of the deduced amino acid sequence for the putative cytochrome p450 (contig 69591) identified exclusively in the Haller's organ spf transcriptome of unfed virgin adult male *Dermacentor variabilis* versus the top BLAST GenBank hit (lowest expect value; Table 4). Asterisks (*) denote conserved residues, colons (:) indicate conservation between groups of strongly similar properties scoring >0.5 in the Gonnet PAM 250 matrix, and periods (.) indicate conservation between groups of weakly similar properties scoring ≤0.5 on the Gonnet PAM 250 matrix. The black bar shows the cytochrome p450 domain. The acronym consists of the first letter of genus and species (*Ixodes scapularis,* Is) followed by the GenBank accession number for the protein BLAST hit (EEC03681.1).

```
contig_01691      ------------------GNAPQKWSLQNTIKETLRTATVEPWTARCQDVDIDIAGHIIP
RpJAA56317.1      TIAKEVKETVDKTKGLSCQALLSMKSLQNSIKETLRTATIEPWAARCQDVDIDIAGHIIP
                                        .  ****:*********:***:****************

contig_01691      KKTPVIQALGVVLHEQENWKVPQRFDASRFKDSPQDQLAYCPFGFAGKRQCPGQDFSVLV
RpJAA56317.1      KKTPVIQALGVVLHEQENWKVPQRFDASRFEDAPEDQLAFCPFGFAGKRQCPGQDLSILV
                  ******************************:*:*:****:***************:*:**

contig_01691      MATFLATLCPVLTLHSVSDQVVTPTSALITRPQDEVWVTLKKR*SGLASRRDSLPCPTQL
RpJAA56317.1      MGVFLATLCPVLKLHFVPDQVVTPMSALITRPQDEVWVTLKKR-----------------
                  *..*********.** * ****** ******************
```

Figure S25. Multiple sequence alignment (Clustal Ω) of the deduced amino acid sequence for the putative cytochrome p450 (contig 1691) identified exclusively in the Haller's organ spf transcriptome of unfed virgin adult male *Dermacentor variabilis* versus the top BLAST GenBank hit (lowest expect value; Table 4). Asterisks (*) denote conserved residues, colons (:) indicate conservation between groups of strongly similar properties scoring >0.5 in the Gonnet PAM 250 matrix, and periods (.) indicate conservation between groups of weakly similar properties scoring ≤0.5 on the Gonnet PAM 250 matrix. The black bar shows the cytochrome p450 domain. The acronym consists of the first letter of genus and species (*Rhipicephalus pulchellus*, Rp) followed by the GenBank accession number for the protein BLAST hit (JAA56317.1).

```
contig_06898      DFSVSLIVHFLLPGVAKFFRLKFFNPDTLEYFRSLCQRVIKGRIDTKIRQDDFLQHMIDC
IsEEC19065.1      ---------------------------------------METRRKTKTKQDDFLQIMIDA
                                                         :: * .** :****** ***.

contig_06898      QQGTYSGDTSKEVADTEERIFDVDSKLADTEDVPSNALSEEEAMAQCFMFLIAGQGTTST
IsEEC19065.1      QERNRTLDVSQGGEEDAVKLFDIDSKLTDEAPLSSKTLSEEEALSQCMMFILAGHGTTSS
                  *: . : *.*:   :   ::**:****:*   : *::******::**:**::**:****:

contig_06898      LVAFTLYMLALNPDVQEKLREEVDLCVKNHGEYPAMEVVAKLEYLHGVISEMLRMFPPAS
IsEEC19065.1      VIAFSLYLLALNPEAQNKLRKEVDVCVKENGPKPSMDAIDKLQYLHGVVSEALRIFPPAS
                  ::**:**:*****:.*:***:***:***:.*  *:*:.: **:*****:** **:*****

contig_06898      RLERETTQDYVLGDTGIKIPKGCVIAVPLYAMHHDPEYFPDPYVFRPERFMGENAANIRP
IsEEC19065.1      RLERETTEDYVLGNTGIKVPKGCVVAVPVWALHHDPQYFPDPHSFKPERFSKENVDSIPP
                  *******:*****:****:*****:***::*:****:*****: *:****  **. .* *

contig_06898      YTYLPFGAGPRNCVGMRLGLHAAKMAVLHAVRIAQFVRTDKTKVPLEFFKGFGVISSSDI
IsEEC19065.1      YVYLPFGAGPRNCIGVRLGLRAVKMALFHSICNVEFVRTAKTKVPLELFKGFGVISSSDI
                  *.***********:*:****:*.***::*::  .:**** *******:************

contig_06898      TVGVRKRAATSK*NIHIDLKAI*TPTPLLIYLDFFA**FRKVTSDFHSEDNTMTLTESAV
IsEEC19065.1      TVGVRKRTS---------------------------------------------------
                  *******::
```

Figure S26. Multiple sequence alignment (Clustal Ω) of the deduced amino acid sequence for the putative cytochrome p450 (contig 6898) identified exclusively in the Haller's organ spf transcriptome of unfed virgin adult male *Dermacentor variabilis* versus the top BLAST GenBank hit (lowest expect value; Table 4). Asterisks (*) denote conserved residues, colons (:) indicate conservation between groups of strongly similar properties scoring >0.5 in the Gonnet PAM 250 matrix, and periods (.) indicate conservation between groups of weakly similar properties scoring ≤0.5 on the Gonnet PAM 250 matrix. The black bar shows the cytochrome p450 domain. The acronym consists of the first letter of genus and species (*Ixodes scapularis*, Is) followed by the GenBank accession number for the protein BLAST hit (EEC19065.1).

```
contig_14383      ------ARTNIQRGDASSLALYKAARKSVGQFGGSKLFFLNLLPPSRLLHKIIFAVQSIF
AtJAC34536.1      LQFFAGALTDVQRNDAAAVALSEAARQSVGQFGGAVLFLLNLLPDSPLLHKTLLGVRSLF
                        * *::** **:::** :***:*******: **:***** * **** ::.*:*:*

contig_14383      TQLPSDEVIERMLPIINHRRENPDPTKEDLLQLLLNSEKEDRKNNGKIEGLESSSIMMSH
AtJAC34536.1      TQLPSDEMMDRMTPIINHRREHPDPTKEDVLQLLLNSEQEELSSNGKAEGRQLSSTMMSH
                  *******:::** ********.*******:********:*: ..*** ** : ** ****

contig_14383      PLELRTASNTAICVIAGMDNIASPLAFASYLLSEHQEVQDKVRAEVQALLKKEGELTYDG
AtJAC34536.1      PLTLRTASNTCIFVIASIDAVASPLAFTSYLLSEHPDIQEKVRTEVQAILKKEGKFTYEN
                  ** *******.* ***.:* :******:******* ::*:***:****:*****::**:

contig_14383      LGELTYLGQVLSETLRLYPALPGWVPRVCDEDYEYNGVRILKGMSVSVLPLDVHYDPVLW
AtJAC34536.1      IMELTYLGQVLSESLRLYPSLPGSIRRICDEDYEHNGVRILKGMNVSVPTLDLHYDPELW
                  : ***********:*****:*** : *:******:*********.***  **:**** **

contig_14383      PEPKKFDPERFSKANKDRIHPFSYFPYGIGPRTCMATLLSRVEFLVTLSLLVMRYRLLPS
AtJAC34536.1      PQPKKFDPERFSKANKDNIRPMSYFPFGFGPRRCIASALSQMELTLVLAMLVARYRILPS
                  *:***************.*:*:****:*:*** *:*: **::*: :.*::** ***:***

contig_14383      GKYKNEPPKYFTAALAGFPKEGVFVKLQKLQNP*KHL*QSEHVMVIITDYFFHVYNNNCF
AtJAC34536.1      GRYEKEPPAYASSSLLGFPKHGIWVKLEKL------------------------------
                  *:*::*** * :::* ****.*::***:**
```

Figure S27. Multiple sequence alignment (Clustal Ω) of the deduced amino acid sequence for the putative cytochrome p450 (contig 14383) identified exclusively in the Haller's organ spf transcriptome of unfed virgin adult male *Dermacentor variabilis* versus the top BLAST GenBank hit (lowest expect value; Table 4). Asterisks (*) denote conserved residues, colons (:) indicate conservation between groups of strongly similar properties scoring >0.5 in the Gonnet PAM 250 matrix, and periods (.) indicate conservation between groups of weakly similar properties scoring ≤0.5 on the Gonnet PAM 250 matrix. The black bar shows the cytochrome p450 domain. The acronym consists of the first letter of genus and species (*Amblyomma triste*, At) followed by the GenBank accession number for the protein BLAST hit (JAC34536.1).

```
contig_12057      PAVAARAGGPGLNDAPSSALLATATTRQTRNQQSLSRGLSFSVSGSQKGAASSSAITMPV
AtJAC32978.1      ---------------------------------------------------------MPI
                                                                           **:

contig_12057      VLYNLVGSPPCGFIRCLAKHIGVELNLNNLDFAKGEHRTEQFLKVNPFHKVPAIDDDGFI
AtJAC32978.1      VLYNLNGSPPCGFIRSLAKEIGVELSVKTLDFAKKEHLSEDFLKVNPFHKVPTIDDDGFI
                  ***** *********.***.*****.::.***** ** :*:***********:*******

contig_12057      VYESNAIAYYLLRKYSPESDLYPACIETRTRIDQVLAAASSNIHPQLGAFFRPRYFQSTK
AtJAC32978.1      VYESNAIAYYLLRKYAPESDLYPNCLRGRTRIDQVLAAASSNIQASLGAFFRPRFFQHTK
                  ***************:******* *:. ***************: .********:** **

contig_12057      PSAEEVKAFEENVVKNLENLIGDSKFAVGDKLTAADFCLIGHVTVCLEFPCVDKAKYPKL
AtJAC32978.1      PTDEEVSAFEQNVCKGLENLIGDKKFAVGDKITLADLCLIGHVTLVIEFGYVDKAKYPKL
                  *: ***.***:** * *******.*******:* **:*******: :**  *********

contig_12057      TAYYELVRNTLPYYQEIFGPFTAQTKQLWDRLK*PLFAPLPQRN*D*ARREMGAVGVLK*
AtJAC32978.1      SGYYELVKSELPYFDEVYGPAVSALKEALAKLK---------------------------
                  :.*****:. ***::*::** .:  *:   :**
```

Figure S28. Multiple sequence alignment (Clustal Ω) of the deduced amino acid sequence for the putative glutathione S-transferase (contig 12057) identified exclusively in the Haller's organ spf transcriptome of unfed virgin adult male *Dermacentor variabilis* versus the top BLAST GenBank hit (lowest expect value; Table 4). Asterisks (*) denote conserved residues, colons (:) indicate conservation between groups of strongly similar properties scoring >0.5 in the Gonnet PAM 250 matrix, and periods (.) indicate conservation between groups of weakly similar properties scoring ≤0.5 on the Gonnet PAM 250 matrix. The solid black bar shows the glutathione S-transferase amino terminal domain, and the dashed black bar shows the glutathione S-transferase carboxyl terminal domain. The acronym consists of the first letter of genus and species (*Amblyomma triste*, At) followed by the GenBank accession number for the protein BLAST hit (JAC32911.1).

```
contig_04931      AARSGPGMPRRPVVGYWNVRGLGQYIRNLLVYKGVAFEDKLYRFGPPPDFDRSHWHGEKF
AtJAC32978.1      -------MPRRPVVGYWNVRALGQHIRNLLIYKGVAFEDKLYRFGPAPDFDRSHWLGEKF
                         *************.***:*****:*************** ******** ****

contig_04931      SLGLQFPNLPYYIDGDVKITQSLAIMRYLARKHDLGARNDEETLQLDFLEQQARDLAWGL
AtJAC32978.1      SLGLQFPNLPYYIDGDVKITQSLAILRYLARKHDLAARNEQEMLQMDLLEQQAKDLAWGL
                  *************************:*********.***::* **:*:*****:******

contig_04931      AMTAFNPTFDEARKKYEENLVNVLKPWANHMRDCTWALGDRLTYVDFLLYEALDWNHEFN
AtJAC32978.1      AMTAFNPTFDEARKKYEENLVTVLKPWSDLMRDRVWVLGDRLTYVDFLLYEALDWNHEFN
                  *********************.*****:: *** .*.***********************

contig_04931      ADAFAGYPELQQYLMRFEELPNIKEYFASENYSKWPILGPMVKWGHFKE*LRDFAS*YRP
AtJAC32978.1      PDAFSGYPVLLEYLRRFEELPNIKEYFASENYSKWPILGPMAKWGHFKE-----------
                   ***:*** * :** **************************.*******
```

Figure S29. Multiple sequence alignment (Clustal Ω) of the deduced amino acid sequence for the putative glutathione S-transferase (contig 4931) identified exclusively in the Haller's organ spf transcriptome of unfed virgin adult male *Dermacentor variabilis* versus the top BLAST GenBank hit (lowest expect value; Table 4). Asterisks (*) denote conserved residues, colons (:) indicate conservation between groups of strongly similar properties scoring >0.5 in the Gonnet PAM 250 matrix, and periods (.) indicate conservation between groups of weakly similar properties scoring ≤0.5 on the Gonnet PAM 250 matrix. The solid black bar shows the glutathione S-transferase amino terminal domain, and the dashed black bar shows the glutathione S-transferase carboxyl terminal domain. The acronym consists of the first letter of genus and species (*Amblyomma triste*, At) followed by the GenBank accession number for the protein BLAST hit (JAC32978.1).

```
contig_83534      --------------------GCLSTGSHYNPNNKNHGAPNAEDRHVGDLGNIVADC-GIA
RpJAA58838.1      TGLQPGAHGLHVHSYGDLTNGCNSTKGHFNPMHKDHGGPEDRERHVGDLGNIKAEADGKA
                                      ** ** .*:** .*:**.*: .:********* *:. * *

contig_83534      VVNLTDHLLTLNGENSIIGRAVVVHADEDDLGLGSHNDSKTTGHAGSRLTCCVIGIARNS
RpJAA58838.1      RVYITDSMISLVGHHNIIGRAMVVHANPDDLGKGGTNESKTTGSAGPRLACCVIGFVSGS
                   * :** :::* *...*****:****: **** *. *:***** ** **:*****:.  *
```

Figure S30. Multiple sequence alignment (Clustal Ω) of the deduced amino acid sequence for the putative superoxide dismutase (contig 83534) identified exclusively in the Haller's organ spf transcriptome of unfed virgin adult male *Dermacentor variabilis* versus the top BLAST GenBank hit (lowest expect value; Table 4). Asterisks (*) denote conserved residues, colons (:) indicate conservation between groups of strongly similar properties scoring >0.5 in the Gonnet PAM 250 matrix, and periods (.) indicate conservation between groups of weakly similar properties scoring ≤0.5 on the Gonnet PAM 250 matrix. The black bar shows the copper/zinc superoxide dismutase domain. The acronym consists of the first letter of genus and species (*Rhipicephalus pulchellus,* Rp) followed by the GenBank accession number for the protein BLAST hit (JAA58838.1).
